# The dynamic side of the Warburg effect: glycolytic intermediate storage as buffer for fluctuating glucose and O _2_ supply in tumor cells

**DOI:** 10.12688/f1000research.15635.2

**Published:** 2018-12-28

**Authors:** Johannes H.G.M. van Beek

**Affiliations:** 1 Experimental Vascular Medicine, Amsterdam University Medical Centers, location AMC, Amsterdam, 1105 AZ, The Netherlands; 2 Clinical Genetics, Amsterdam University Medical Centers, location VUmc, Amsterdam, 1081 BT, The Netherlands

**Keywords:** glycolysis, cancer, hypoxia, cycling hypoxia, nutrient shortage, computational model, cancer metabolism, oxidative phosphorylation, nutrient fluctuation

## Abstract

**Background**: Tumor cells often show altered metabolism which supports uncontrolled proliferation. A classic example is the Warburg effect: high glucose uptake and lactate production despite sufficient oxygen supply. Remarkably, tumor cells can transiently take up glucose even an order of magnitude faster when glucose is reintroduced after depletion. Regulation and significance of this high glucose uptake are investigated here.

**Methods**: A new computational model was developed which reproduces two types of experimental data on Ehrlich ascites tumor cells: measurements by Otto Warburg of the average aerobic glycolytic rate during one hour (Warburg effect), and fast metabolic responses measured by others during the first minutes after reintroducing glucose. The model is subsequently extended with equations for glucose and O _2_ transport to predict the role of metabolism during fluctuations of blood flow in tumor tissue.

**Results**: Model analysis reveals dynamic regulation of the head section of glycolysis where glucose uptake and phosphorylation occur. The head section is disinhibited slowly when concentrations of glycolytic intermediates fall, causing glucose uptake rate to considerably exceed that found by Warburg. The head section is partially inhibited in about a minute when sufficient glucose has been taken up. Simulations predict that tumors greedily take up glucose when blood flow resumes after periods of low flow. The cells then store glucose as fructose 1,6-bisphosphate and other glycolytic intermediates. During subsequent periods of low flow that cause O _2_ and glucose depletion these stores are used for ATP production and biomass.

**Conclusions**: The powerful glycolytic system in tumors not only synthesizes ATP at high steady rates, but can also store glycolytic intermediates to buffer temporary oxygen and nutrient shortages for up to 10 minutes. The head section of glycolysis in tumor cells, disinhibited during glucose shortages, becomes very efficient at stealing glucose from other cells, even at low glucose concentrations.

## Introduction

Altered metabolism is an important characteristic of cancer cells and supports uncontrolled cell proliferation. For that reason cancer metabolism provides targets for therapeutic interventions. Cancer cells often show high lactate production despite sufficient oxygen supply, a phenomenon discovered by Otto Warburg and termed the Warburg effect after him ^1^. This forms the classic example of the widespread metabolic reprogramming in cancer ^2– 4^. Warburg’s favorite experimental system to study this aerobic glycolysis were suspensions of mouse Ehrlich ascites tumor cells (EATC) ^1, 5, 6^, which showed high aerobic glycolytic rates persisting for hours as long as glucose concentration remained high. These EATC were later also used by Warburg’s contemporaries to study the kinetics of metabolic responses in the first seconds and minutes after glucose addition to cells previously depleted of glucose ^7, 8^. They found that glucose uptake is much higher in the first minute after glucose reintroduction than averaged over one hour. The results of these experiments were explained by Chance and Hess with a mathematical model, which may have been the first digital computer model of a metabolic system ^7, 9^. Their model, although ingenious, contained some biochemical assumptions that are now considered untenable. In the present study, a small computational model is developed that economically reproduces the experimental results of the kinetic as well as the steady-state experiments on EATC, and at the same time provides a testable model of the dynamic regulation of energy metabolism in the ascites tumor cells. Analysis of the new model in the present paper suggests that the head section of glycolysis can sequester glucose at very high capacity, but is downregulated quickly if glucose is taken up, falling to the still high steady-state levels of the Warburg effect. The glycolytic head section is disinhibited slowly if glycolytic intermediates levels in the cells fall, preparing the tumor cells to take up glucose with very high capacity if it becomes available after a period of low supply.

Because the new metabolic model reproduces the behavior of the ascites tumor cells well for conditions with variable glucose levels, it is subsequently used to investigate the possible physiological role of this dynamic metabolic regulation in the tumor cells. Blood flow and the supply of oxygen and nutrients is often fluctuating in tumor tissue, a phenomenon referred to as cycling hypoxia ^10– 12^. To investigate the role of the dynamic behavior and regulation of metabolism in the tissue environment, the computational model of intracellular metabolism is extended with equations for oxygen and glucose transport in tumors with cycling blood flow. The simulations reported here suggest that tumor cells can store glucose-derived metabolites to maintain ATP and carbon substrate levels during periodic oxygen and glucose shortages, which are commonly found in tumor tissue ^11, 13^. As a result, cells with lower glycolytic capacity than tumor cells can have sufficient energy supply at constant blood flow, but their energy supply fails in conditions with fluctuating blood flow while tumor cells with high glycolytic capacity still do well. Stored glycolytic intermediates may be able to maintain energy supply at a level sufficient to maintain cell viability for up to 10 minutes.

## Methods

### Development of the computational model

**Figure 1.**
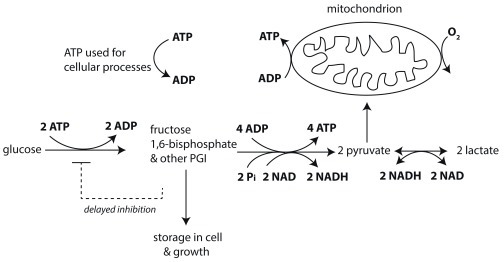
Scheme of computational model of tumor cell metabolism. In the head section of glycolysis, 2 ATP are spent to phosphorylate glucose, resulting in phosphorylated glycolytic intermediates (PGI) with fructose 1,6-bisphosphate (FBP) as major species. In the tail section of glycolysis four ATP, two reduced nicotinamide adenine dinucleotide (NADH) and two pyruvate molecules are produced per metabolized FBP and two inorganic phosphate (P _i_) molecules are taken up. Pyruvate molecules can be converted to lactate while producing oxidized NAD. Pyruvate and NADH are also substrates for mitochondrial oxidative metabolism. ATP is used for growth, proliferation and maintenance tasks such as ion pumping. Increased NADH concentration reduces flux in the tail section. Signals from the PGI pool inhibit the head section with a time delay.

The simplified computational model developed and applied in this study comprises glycolysis, oxidative phosphorylation, ATP consumption and their interactions in the tumor cell ( Figure 1). The goal of the model is to reconstruct the glucose uptake behavior and the dynamic balance of ATP, phosphorylated metabolites, glucose-derived metabolites, lactate production and NADH/NAD redox status in the cell, especially in the first minutes after resupply of glucose. In addition, it also reproduces three effects which persist on the order of an hour or longer: i) the Warburg effect ^1, 6^: high glycolytic rate despite abundant oxygen availability; ii) the Pasteur effect ^7^: increase in glycolytic rate when oxygen is depleted; and iii) the Crabtree effect ^6, 14^: decrease in oxygen uptake after addition of glucose. Existing detailed models of glycolysis containing equations for each enzymatic step in glycolysis ^15– 17^ were tried, but did not reproduce the available experimental results satisfactorily. A major problem was also that these models contain too many parameters to estimate from the available data (see Supplementary Text). Therefore a coarse-grained model was developed which could be parameterized using available metabolic data for the key nodes in the model. The computational model consists of rate equations for the head and tail part of glycolysis, oxidative phosphorylation, ATP consumption and the lactate dehydrogenase reaction which together determine the rate of change of key metabolite concentrations in a system of ordinary differential equations. The model is not meant to be a detailed reconstruction of the enzyme reactions involved and their regulatory mechanisms, but focuses on reproduction of the metabolic responses of the cell which were measured experimentally. The model also retains the overall structure of the metabolic system and key regulatory loops. Despite its simplification, this small model reproduces the three steady-state effects as well as a range of kinetic data with satisfactory quantitative approximation.

Chance and Hess ^7, 9^ already had developed a digital computer model to explain measurements of transients in glucose metabolism and mitochondrial respiration in EATC. This was probably the first digital model of a biochemical system ever published. Although this was an ingenious pioneering effort, the model’s assumptions are not compatible with present biochemical knowledge: oxidative phosphorylation, for instance, was assumed to occur via a phosphorylated high energy intermediate and not via a chemiosmotic mechanism, and mitochondria were assumed to retain the ATP they synthesized until an uncoupling agent was applied. Therefore a new model was developed here.

Although glycolysis has been extensively studied, it is presently still difficult to construct a fully detailed accurate model of this pathway ^18^. Attempts to simulate the response of EATC using existing detailed equations for all enzymatic steps in glycolysis failed to reproduce essential features of the metabolic responses (see Supplementary Text). Therefore, a simplified representation of glycolysis by a head and tail section is used, similar to that in earlier conceptual models ^19^. This approach is also taken in recent computational ^20, 21^ models for yeast glycolysis to investigate robustness, efficiency, oscillations, and failure to start up. Consequently, the new model incorporates a parsimonious description capturing the essential kinetic properties of the glycolytic system in mammalian cells. Two kinetic equations represent the head and tail sections of glycolysis upstream and downstream of fructose 1,6-bisphosphate (FBP). These two equations make it possible to calculate the time course of the FBP pool, which can be directly compared with measurements in the experimental data sets. FBP usually also is by far the most abundant species of the phosphorylated glycolytic intermediates (PGI). The new model presented here further incorporates a simple description of oxidative phosphorylation in the mitochondria, which responds to ADP, inorganic phosphate (P _i_) and oxygen concentrations. The equations are discussed in detail in the Supplementary Material. The state variables of the model are given in Supplementary Table 1 and the metabolic fluxes in Supplementary Table 2.

The head section of glycolysis comprises glucose transport across the cell membrane and the hexokinase, glucose 6-phosphate isomerase and phosphofructokinase enzymes, which catalyze the double phosphorylation of hexose to FBP. The rate equation for the glycolytic head section depends on glucose and ATP concentrations, the two substrates used in this section. The interaction of glucose and ATP in determining the rate of the head section is modelled as in kinetic equations for mammalian hexokinase ^15, 16^, the first enzyme in the head section and an important control point of glycolytic rate limitation in cancer cells ^22^. The good fit to glucose uptake and fructose 1,6-bisphosphate measurements (see below) depends directly on this equation because they are the input and output of the head section, suggesting that this simplified representation of the head section is adequate for the purpose of this study.

In tumor cells there is strong negative feedback of glucose 6-phosphate (G6P) on hexokinase, the first enzyme of the head section of glycolysis ^22^. In addition to feedback by G6P, feedback by FBP has also been reported in ascites tumor cells ^23^. The feedback control on the head section of glycolysis by downstream intermediates shows a clear time delay and affects the glycolytic rate in the head section with a half time >10 s ^24, 25^. Binding of G6P to hexokinase also may lead to translocation of this enzyme with a similar time course ^26^. The delayed negative feedback from the PGI pool on hexokinase is represented in the model by a second order reaction of PGI with the head section, governed by a second order forward rate constant and a first order backward rate constant (see Eq. 22 in Supplementary Text). The forward reaction inactivates the head section and the backward reaction reactivates the inactivated head section. The activation state of the head section is represented by the active fraction, F _active_. Representation in this simple form adequately describes the time delay of activation and reactivation. The delay in inhibition of the head section reproduces the decrease in glucose uptake and overshoot in FBP concentration after glucose addition to the cell suspension, whereas previously ATP trapping in the mitochondria ^7, 9^ or complex regulatory interactions between two compartmentalized glycolytic systems had to be hypothesized ^19^ to account for this time course.

The product of the head section is FBP, which is by far the most abundant phosphorylated glycolytic intermediate (PGI) and is therefore directly represented in the model. However, the other PGIs, consisting of glucose 6-phosphate, fructose 6-phosphate, dihydroxyacetone phosphate, 3-phosphoglycerate, etc., are also taken into account in the storage of glucose-derived metabolites. They are lumped with FBP in the total PGI pool with a model parameter representing the fixed ratio between the sum of all phosphorylated glycolytic intermediates and FBP. In this way the total PGI content is taken into account in the time-dependent mass balance calculations.

The tail section of glycolysis in the model is downstream of the FBP pool. It consists of the glycolytic enzymes aldolase, triose phosphate isomerase, glyceraldehyde 3-phosphate dehydrogenase (GAPDH), phosphoglycerate kinase, phosphoglycerate mutase, enolase and pyruvate kinase. Input reactants for the tail section are FBP, NAD ^+^, ADP and inorganic phosphate (P _i_), while its products are pyruvate, NADH and ATP. Equation 2 in the Supplementary Text represents the lumped tail section in a simplified way. The rate increases with the concentrations of the reactants FBP, NAD and ADP in a saturable way and is decreased by the product NADH, governed by an inhibition constant ^27^. The mathematical expression for stimulation of the flux in the tail section of glycolysis by FBP reflects not only its role as a direct substrate, but also its potential role in activation of pyruvate kinase in the micromolar range ^28^. More detailed considerations are given in the Supplementary Text.

The equations for flux in the head and tail section of glycolysis incorporate the assumption that the reverse fluxes in the direction pyruvate-to-glucose are negligible. Simulations with model equations for cancer cell glycolysis ^16^ and with model equations for normal mammalian cells ^15, 17^ indicate that in at least one enzyme in both the glycolytic head and the tail section the reverse fluxes are at least two orders of magnitude smaller than the forward flux (see Supplementary Text). EATC do not contain appreciable amounts of glucose 6-phosphatase, in contrast to liver which can synthesize glucose ^29^. Reverse fluxes are therefore considered negligible in the model. A limitation of the present model is therefore that it is unsuitable to quantitate reverse fluxes.

**Figure 2.**
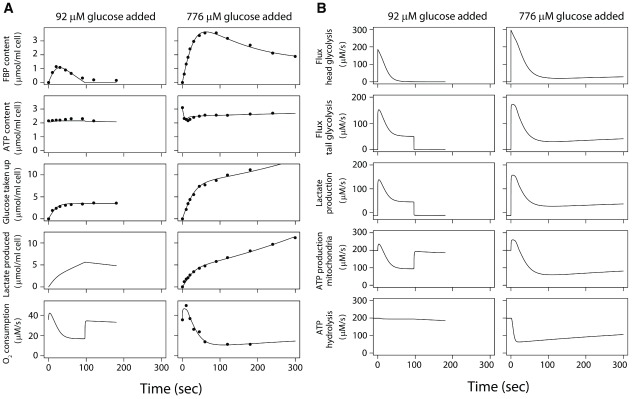
Response of Ehrlich ascites tumor cells *in vitro* to addition of different amounts of glucose. ( **A**) Glucose concentration was zero at t<0, and the cells respired on endogenous substrates, such as lactate. Glucose was added at t=0. Data for experiments (dots) and model fit (lines). Left hand column: low initial glucose concentration (92 µM) was added at t=0 to a suspension of 2.2 volume percent tumor cells. Right hand column: a higher glucose concentration (776 µM) was added at t=0 to a suspension of 2.9 volume percent tumor cells. Contents of fructose 1,6-bisphosphate (FBP), ATP, total glucose taken up and total lactate produced since t=0 are given in µmol/ml cell volume. The rate of O _2_ consumption is given in µmol/liter intracellular water/sec. ( **B**) Response of metabolic fluxes in Ehrlich ascites tumor cells *in vitro* to addition of different amounts of glucose. Same simulation as in Figure 2A. The fluxes are given in µmol/liter intracellular water/sec (µM/s). The fluxes shown here result in the changes in metabolite concentrations, oxygen consumption, glucose uptake and lactate production seen in Figure 2A. The flux in the glycolytic head section is given in 6-carbon units and the flux in the tail section in 3-carbon units. The tail section of glycolysis synthesizes 2 ATP per 3-carbon unit. Note that for t<0 lactate is taken up and used for mitochondrial ATP production in the absence of glucose.

The equation for the lactate dehydrogenase reaction, pyruvate + NADH ⇌ lactate + NAD ^+^, accounting for forward and reverse fluxes was taken from Lambeth and Kushmerick ^17^. This allows for the uptake of lactate for mitochondrial metabolism when glucose is absent as well as lactate production when glucose is abundant. ATP consumption for maintenance, growth and cell function correlates linearly with the fall in adenine nucleotide concentration (ATP+ADP) in the experimental data, as found in the experiments of Figure 2A. This relation was incorporated in the model and reproduces the steep decline in ATP hydrolysis which was found after acutely giving glucose to cells after these had been deprived of glucose for some time (see detailed description in the Supplementary Text, Equations 5 and 6). The model further contains a simple description of oxidative phosphorylation in the mitochondria, which responds to ADP, inorganic phosphate (P _i_) and oxygen concentrations ( Supplementary Text, Eq. 3). This equation is compatible with biochemical knowledge and has been used to investigate the functional significance of the creatine kinase energy buffer system in muscle ^30^.

The ordinary differential equations determining rates of change of metabolite levels represent balances for key players in the model: the balance of phosphoryl groups in the ATP, ADP, FBP and other PGI pools, which play a central role in energy metabolism; the balance of carbon metabolites representing the distribution and storage of glucose; the balance of reduction of NAD to NADH and the reverse oxidation reaction, i.e. the NADH/NAD redox balance. The differential equations are given in detail in the Supplementary Text.

The present model provides only a coarse representation of regulatory mechanisms active *in vivo*, but it fulfills the goal of reproducing a broad range of measurements on EATC, both the average glucose and oxygen uptake measurements during 1 hour in Warburg’s laboratory ^5, 6^, as well as kinetic responses of glucose and oxygen uptake, lactate production, FBP and ATP levels measured in the first seconds and minutes following glucose addition ^7, 31– 34^. The model is subsequently used to investigate what the physiological role is of the high glycolytic capacity for the survival and growth of cancer cells.

The model equations for the metabolic model are all given in the Supplementary Text, where the assumptions underlying the model are discussed further. The computational methods for integrating the system of ordinary differential equations, for parameter estimation and for uncertainty analysis are also given in the Supplementary Text.

To simulate the response of cells represented by the metabolic model to conditions in tissue, the equations for metabolism were extended with well-established equations for transport of oxygen and glucose in the blood and with diffusion equations for transport from blood vessel into tissue in a cylindrical geometry, given in the Supplementary Text. The values for transport parameters are given in Supplementary Table 3. The values for blood flow rate and plasma metabolite concentrations were inspired by experiments on tumors implanted in rats ^35^, while the metabolic parameters for the simulated cells were set as determined in EATC *in vitro* (see above). The maximal diffusion distance in tissue was set to 40 μm from the blood vessel, as found for the supply region of the blood vessels in the experiments on implanted tumors which were used as the example condition for the tissue simulations ^35^.

## Results

### Model analysis of kinetic data on ascites tumor cell suspensions *in vitro*

A data set was assembled consisting of representative experiments from the literature to be used to estimate parameters for the model of metabolic responses of EATC (see Supplementary Text). The data sets are exemplary, but they are representative of results measured in many laboratories ^6– 8, 19, 31– 34, 36– 44^. All selected experiments were done at 37°C on EATC that had been grown in mice. During the experiments, aerated tumor cell suspensions were diluted in buffer solution. Cells and suspension had been depleted of glucose for some time and were respiring on endogenous substrates such as lactate, which was abundantly present. At t=0, glucose was added to the suspension. Two kinetic data sets for the first 3–5 min consist of responses to addition of 92 µM and 776 µM glucose to cells which had been grown in ascites fluid in mice and suspended in media without glucose.

These measured responses of glucose-depleted EATC to addition of low concentrations of glucose are shown in Figure 2A. The model is calibrated ( Supplementary Text) on these data sets ^8, 34^. After adding 92 µM glucose initially ^34^, glucose uptake fell soon because the small amount of glucose was exhausted ( Dataset 1, experiment 1) ^45^. FBP accumulated in the cell briefly, but fell after half a minute, providing the substrate for a major part of the ATP synthesis in the tail section of glycolysis.

After adding 776 µM glucose ^8^, the glucose uptake rate was ~295 µM/s initially and lactate production rose to 157 µM/s in 5 s ( Dataset 1, experiment 2) ^45^. Glucose uptake was subsequently reduced by >90% within 90 s ^7, 8, 32, 42^. In contrast to the experiment with the low glucose concentration, there was still sufficient glucose to support uptake at virtually undiminished rate, but delayed feedback inhibition by glycolytic intermediates on the head section of glycolysis causes the decline in glucose uptake during the first minute according to the model.

During the first 20 s after adding 776 µM glucose mitochondrial respiration is stimulated ( Figure 2A, right); after 30 s respiration is reduced appreciably below the initial value found before glucose addition ^7, 31^. The model shows that the initial stimulation is due to increases in ADP concentration as a consequence of the ADP production in the head section which accompanies the high phosphorylation rate of the newly added glucose. This agrees with the original model analysis of Chance *et al*. ^7, 9^.

Both simulation with the model and direct calculation of the mass balance from the measured phosphate metabolites reveal an ~70% decline in ATP hydrolysis in the first minute after addition of 776 µM glucose, correlating with the amount of ATP plus ADP broken down to AMP, adenosine, inosine etc. This breakdown is reflected in the decreased ATP level after glucose addition ( Figure 2A, right). After the initial breakdown, adenine nucleotide levels recover in 0.5–1 hour ^46, 47^. The reduction of respiration and mitochondrial ATP production which follows the initial stimulation after glucose addition follows the reduced ATP hydrolysis ( Figure 2B, right), mediated by falling ADP levels. Note that ATP is used by distinct processes: phosphorylation of glucose in the glycolytic head section and ATP hydrolysis to ADP by several cellular processes. These processes follow distinct time courses after glucose addition. The reduced ATP hydrolysis in the cell explains the first phase of the Crabtree effect (reduction of respiration after glucose addition), seen after half a minute in Figure 2A. 

Half of the glucose taken up in the first minute after addition of 776 µM glucose is stored as PGI, mainly in the form of FBP ( Dataset 1, experiment 2). Subsequently, FBP declines ( Figure 2A, right), reflecting the delayed negative feedback on the head section of glycolysis and the time course of flux in the glycolytic tail section. FBP settles at still appreciable levels. At 5 minutes after glucose addition, 18% of the total glucose carbon taken up since t=0 is found intracellularly as PGI, 43% has been excreted as lactate and 34% is stored intracellularly in other forms, e.g. glycogen, nucleosides and amino acids.

Model predictions were subsequently compared with experiments not used for parameter estimation ( Supplementary Text): Warburg’s laboratory measured 63±14 (SD) µM/s lactate production and 19±7 µM/s O _2_ consumption in EATC during 1 hour aerobic incubation with glucose ^6^; the simulation predicts 52.5 µM/s lactate production and 19.8 µM/s oxygen consumption ( Dataset 1, exp 3) ^45^. The model simulation predicts that strong transient changes occur during Warburg’s experiment before the rates settle. The values which he reported may therefore reflect averages of rates which varied substantially during the experiment. This could not have been detected at that time because of the low time resolution of Warburg’s ingenious measurement method. Simulation further predicts that lactate production is increased by 61% during anoxia (Pasteur effect; Dataset 2) ^48^; for comparison, in Warburg’s laboratory lactate production increased by 61±32% (SD) when oxidative phosphorylation was blocked ^6^.

Both in experiments where a range of low glucose concentrations were given ^34, 49^ and in the simulations of those experiments, the peak FBP content reached during the transient levels off above 200 μM added glucose concentration, ( Dataset 1, experiment 4) ^45^. This is consistent with the estimated K _m,glucose_ of 51 µM for the head section ( Supplementary Table 3) and K _m,glucose_ values reported for hexokinase, 46–78 µM ^15^. Fast FBP and lactate accumulation measured at 5 and 10 sec ^8, 49^ after glucose addition agree with the simulations: tumor cells store for instance ~700 µM FBP intracellularly in 10 s if the initial extracellular glucose concentration is merely 77 µM ( Dataset 1, experiment 5) ^45^, demonstrating the high capacity of tumor cells to seize glucose.

The simulations reproduce the persistent inhibition of respiration by glucose, known as the Crabtree effect ^14^: the average reduction over 1 hour after adding 11 mM glucose is 44% ( Dataset 1, experiment 3) ^45^, while a 30±12% (SD) reduction of respiration was measured in Warburg’s laboratory ^6^. The decline of respiration in the first minutes after glucose addition ( Figure 2A) is mainly caused by reduced ATP hydrolysis leading to lower demand for ATP synthesis. The high ATP synthesis ^6^ in the tail section of glycolysis persists during the one hour measurement (Warburg effect) and continues to keep ADP concentration and respiration reduced, causing persistent lower stimulation of mitochondrial metabolism ( Dataset 1, exp 3) ^45^ despite the tendency of ATP hydrolysis to return to its original rate after the initial reduction immediately after addition of a high concentration of glucose. The Crabtree effect reported by Warburg’s laboratory may therefore reflect the weighted average of two phases, an early one dominated by reduced ATP hydrolysis and a later one caused mainly by glycolytic ATP production.

Simulations predict that ATP levels decline by 30% after glucose addition at low pyruvate concentrations because of breakdown to AMP, inosine etc. ( Figure 2A), but when 5 mM pyruvate is added simultaneously, the decline of ATP predicted by the simulation is merely 0.1% and the FBP peak decreases by 21% ( Dataset 3) ^50^; a similar pattern is seen experimentally ^46^. The simulation shows that the pyruvate addition causes much higher removal of NADH via the lactate dehydrogenase reaction. As a consequence, NADH levels increase much less after addition of glucose and inhibition of the tail part of glycolysis by NADH is prevented ^27^. Therefore ATP synthesis by the glycolytic tail section is increased, preventing the strong increase of ADP and AMP causing breakdown of adenine nucleotides after glucose addition. The simulation predicts that the brief stimulation of respiration is also blocked. The increased consumption of FBP by the tail section of glycolysis caused by the high pyruvate concentration explains the lower FBP accumulation after glucose addition. This effect of pyruvate underscores that the accumulation of FBP is the result of the balance of the fluxes in the head and tail section of glycolysis.

In short, the present small model economically integrates experimental data and biochemical knowledge, and quantitatively reproduces experimental results on the Warburg effect, Pasteur effect, Crabtree effect and dynamic responses after addition of glucose. The model simulations show that after a period of glucose shortage, glucose uptake is much faster than measured for the steady Warburg effect, and that fructose 1,6-bisphosphate is accumulated and can be quickly taken up in the cell’s biomass and consumed by the tail end of glycolysis where ATP is synthesized. The return to a glycolysis rate at the steady Warburg effect level is the consequence of inhibition of the head section of glycolysis in about 1 minute when glycolytic intermediates accumulate. Slow disinhibition of the glycolytic head section when glycolytic intermediate levels fall following low glucose supply prepares the cell for uptake of glucose at rates exceeding the Warburg effect when glucose supply is restored. A second mechanism for energy homeostasis suggested by the model consists of reduction of ATP hydrolysis which underlies the first phase of the Crabtree effect.

### Prediction of the function of the dynamic metabolic regulation in the tumor cell

The dynamic regulation of glycolysis and glucose uptake in the tumor cells has been captured in the computational model. Next the role is considered that this dynamic regulation plays in tumor cell physiology. ATP synthesis during hypoxia has long been considered a possible role for the glycolytic system which underlies the Warburg effect. The O _2_ saturation of hemoglobin in the red cells in capillaries in tumor tissue is often low or zero ^51^. O _2_ concentrations are low in tumor tissue ^52^ as well as in the ascites fluid in mice where EATC were grown ^5^. Tumor blood flow sometimes stops temporarily ^53^ and many blood vessels are not perfused over extensive periods ^54^. Fluctuations in tumor blood flow may lead to cycling hypoxia ^11, 55^ and periodic glucose shortages (see simulations below). If O _2_ is still available when glucose is depleted, ATP can be synthesized by oxidative phosphorylation, while the cell burns lactate, fatty acids or glutamine ^56^. If glucose is still present, glycolysis can synthesize ATP even if O _2_ is depleted; however, the environment in solid tumors contains glucose concentrations in the order of a few hundred μM, and in many cases even <100 μM ^57^ which means that glucose stores are limited. Cells die when anoxia is combined with glucose depletion for substantial periods of time ^1^. Figure 2A suggests that tumor cells can store FBP and other PGI during periods of sufficient glucose supply by high blood flow in tissue (“times of abundance”). Periods of low blood flow lead to depletion of O _2_ and glucose (“times of famine”), and the cells could then use the stored PGI to synthesize ATP. For each FBP molecule metabolized in the tail part of glycolysis, 4 ATP molecules are synthesized ( Figure 1). Stored FBP can reach ≥5000 µM, with additionally ≥1200 µM 6-carbon units stored as other PGI species ( Dataset 1) ^45^. This enables the synthesis of at least 4 × (5000+1200) μM = 25 mM ATP from PGI, potentially sustaining a high rate of ATP hydrolysis as found in EATC for >2 min, even after glucose and oxygen are depleted. The reduction of ATP consumption in the model, as seen experimentally *in vitro*, provides an additional protective mechanism: protein, DNA and RNA synthesis are presumably reduced first when ATP levels fall, followed by reduction of sodium and calcium ion pumping ^58– 61^. Warburg established experimentally that supply of one-fifth of the normal growth energy preserved the transplantability of tumor cells for 24 hours ^1^. The reduced level of ATP hydrolysis required for maintaining cell viability may therefore be supported much longer than 2 min (probably at least 10 min) by using FBP and other PGI stores.

The functioning of the FPB storage system of tumor cells is difficult to study experimentally *in vivo*. This may require metabolic measurements at a spatial resolution sufficient to distinguish low and high glycolytic cells. High time resolution to resolve the transient metabolic responses and experimental control of fluctuating O _2_ and nutrient supply is probably also needed. While experimental tests are challenging, the functioning of dynamic regulation of glucose metabolism in tissue may be investigated with computational simulation.

### Simulating tumor cell metabolism during oxygen and glucose fluctuations in tissue

**Figure 3.**
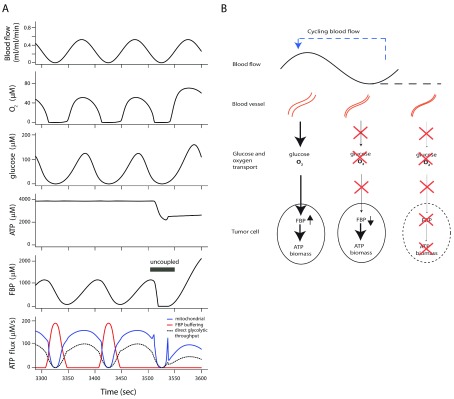
Tumor cell metabolism in tissue during cycling blood flow. ( **A**) Model simulation of tumor cell metabolism in tissue during cycling blood flow, demonstrating ATP synthesis buffered from fructose 1,6-bisphosphate (FBP) stores. All cells have the full tumor glycolytic capacity. ATP synthesis by oxidative phosphorylation (blue line) fails periodically during low blood flow because of low oxygen supply. Glycolytic ATP synthesis by direct throughput of FBP from head to tail section fails because of glucose depletion (dashed black line). A burst of ATP synthesis from the stored fructose 1,6-bisphosphate (FBP) and other phosphorylated glycolytic intermediates (red curve) maintains ATP levels during glucose and O _2_ shortages. In this simulation a steady state had been reached after the transition at t=0 to cycling blood flow. Glycolytic ATP synthesis depending on decreasing levels of FBP was uncoupled between 3505 and 3550 seconds, leading to an immediate fall in ATP level. ( **B**) Scheme of energy and nutrient buffering during fluctuating O _2_ and glucose supply. During high blood flow, FBP and other phosphorylated glycolytic intermediates are stored in the tumor cells. At low blood flow glucose and O _2_ are depleted. Flux in the tail part of glycolysis is maintained by use of previously stored FBP, which is replenished during periods of sufficient blood flow. If blood flow stops for a long time, the intracellular FBP store is depleted and cells perish.

There are limitations to experimental approaches, but the functioning of FBP buffering *in vivo* can be predicted with the present metabolic model, extended with well-known equations for glucose and O _2_ transport by blood flow and diffusion to simulate tumor tissue. The model equations for tissue transport are described in the Supplementary Text. Figure 3 shows a simulation of a hypothetical situation in tissue with blood flow fluctuating around a low average value. Fluctuations in blood flow are a common feature of tumor tissue ^10, 11, 53– 55^. In this simulation a sinusoidal blood flow oscillation at a frequency of one cycle per 100 s is imposed to demonstrate the phenomenon. Blood flow rate, diffusion distance and plasma metabolite concentrations were set to values found in experiments on tumors implanted in rats ^35^, while the metabolic characteristics of the simulated cells are set as determined in EATC *in vitro* (see above). O _2_ and glucose concentrations become virtually zero during the low blood flow phase, and as a consequence flux in the head section of glycolysis ( Figure 3A, dashed curve) and oxidative phosphorylation (blue curve) both stop. Glycolytic ATP synthesis from stored FBP is quickly increased to replace reduced oxidative phosphorylation (red curve). The flux in the tail end of glycolysis responds to the increase of ADP caused by the reduction of oxidative phosphorylation. This regulatory mechanism keeps ATP levels and ATP synthesis virtually constant near the level found at constant high blood flow. Because the flux in the head section is reduced by the fall in glucose concentration, FBP synthesis by the head section is below the level required by the tail section and FBP levels therefore fall. The relative increase in net ATP synthesis by glycolysis is much higher than the relative increase in flux in the glycolytic tail section. This can be understood by considering that ATP synthesis from previously stored FBP does not require simultaneous phosphorylation of glucose in the head section, leading to a more favorable net ATP balance. The complex sequence of events can be examined in the detailed numerical simulation results ( Dataset 4a) ^62^. The FBP store is replenished during the high blood flow phase by the powerful head section of glycolysis ( Figure 3B), although the glucose concentration is initially still low. During this phase the flux in the head section is high, requiring ATP delivered by oxidative phosphorylation and the glycolytic tail section. The effect of ATP synthesis by the FBP buffer mechanism is demonstrated by uncoupling the associated phosphorylation of ADP in the glycolytic tail section. This uncoupling leads to an immediate decrease in adenine nucleotide levels and as a consequence ATP hydrolysis is reduced, owing to the second homeostatic mechanism in the model. This prevents progressive imbalance of ATP hydrolysis and consumption, although both now operate at a lower rate.

**Figure 4.**
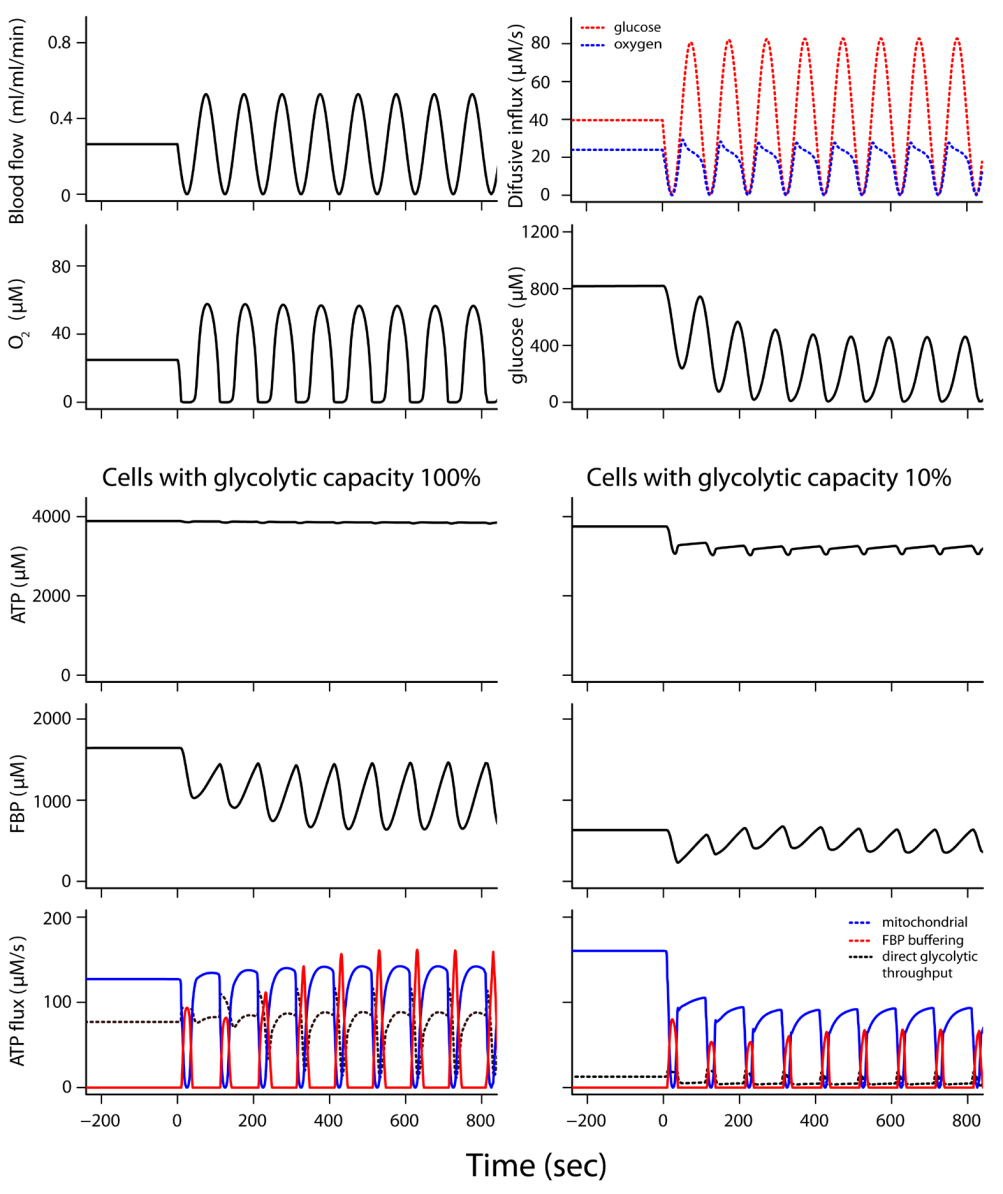
Simulations predicting tumor tissue metabolism during the start of blood flow cycling. In this simulation, 80% of the cell volume had the full tumor glycolytic capacity; 20% of the cell volume had 10% of the full glycolytic capacity. This means that the V _max_ of the head and tail section had 10% of the value for EATC. The top two rows show tissue conditions experienced by both cell types. Blood flow and diffusion flux of glucose and O _2_ from the microvessel into tissue are given (top row). O _2_ and glucose concentrations seen by both cell types are given in the second row. The simulation is for cells at ~18 μm from the microvessel. High ATP consumption, >160 μM/s, was maintained at constant blood flow. See legend to Figure 3 for description of ATP synthesis fluxes. When blood flow started to fluctuate at t=0, ATP synthesis from the stored fructose 1,6-bisphosphate (FBP) and other phosphorylated glycolytic intermediates (red curve) maintained ATP levels and high ATP hydrolysis rates in cells with full tumor glycolytic capacity; however, there was a drop in ATP level and ATP hydrolysis rate in the cells with reduced glycolytic capacity.

The transition from constant to cycling blood flow was simulated ( Figure 4 and Dataset 4b) ^62^, with 80% of the cell volume consisting of tumor cells with full glycolytic capacity as found in the EATC while the remaining 20% of cell volume consists of cells with glycolytic capacity reduced to 10%. As long as blood flow is constant, ATP levels and ATP hydrolysis for cell maintenance and growth are maintained in both cell types. At t=0 blood flow starts to fluctuate, but the average blood flow is still the same. ATP concentration and ATP turnover are now well maintained in the cells with full glycolytic capacity. However, in the cells with 10% of the tumor glycolytic capacity in the same tissue region FBP buffering is appreciably decreased and adenine nucleotide levels and ATP hydrolysis fall quickly after blood flow fluctuations start. The low-capacity glycolytic cells therefore sustain a lower rate of ATP turnover during cycling blood flow. Uncertainty analysis shows that the model predictions are sufficiently constrained ( Supplementary Figure 1, Supplementary Figure 2 and Supplementary Text) ^63^.

**Figure 5.**
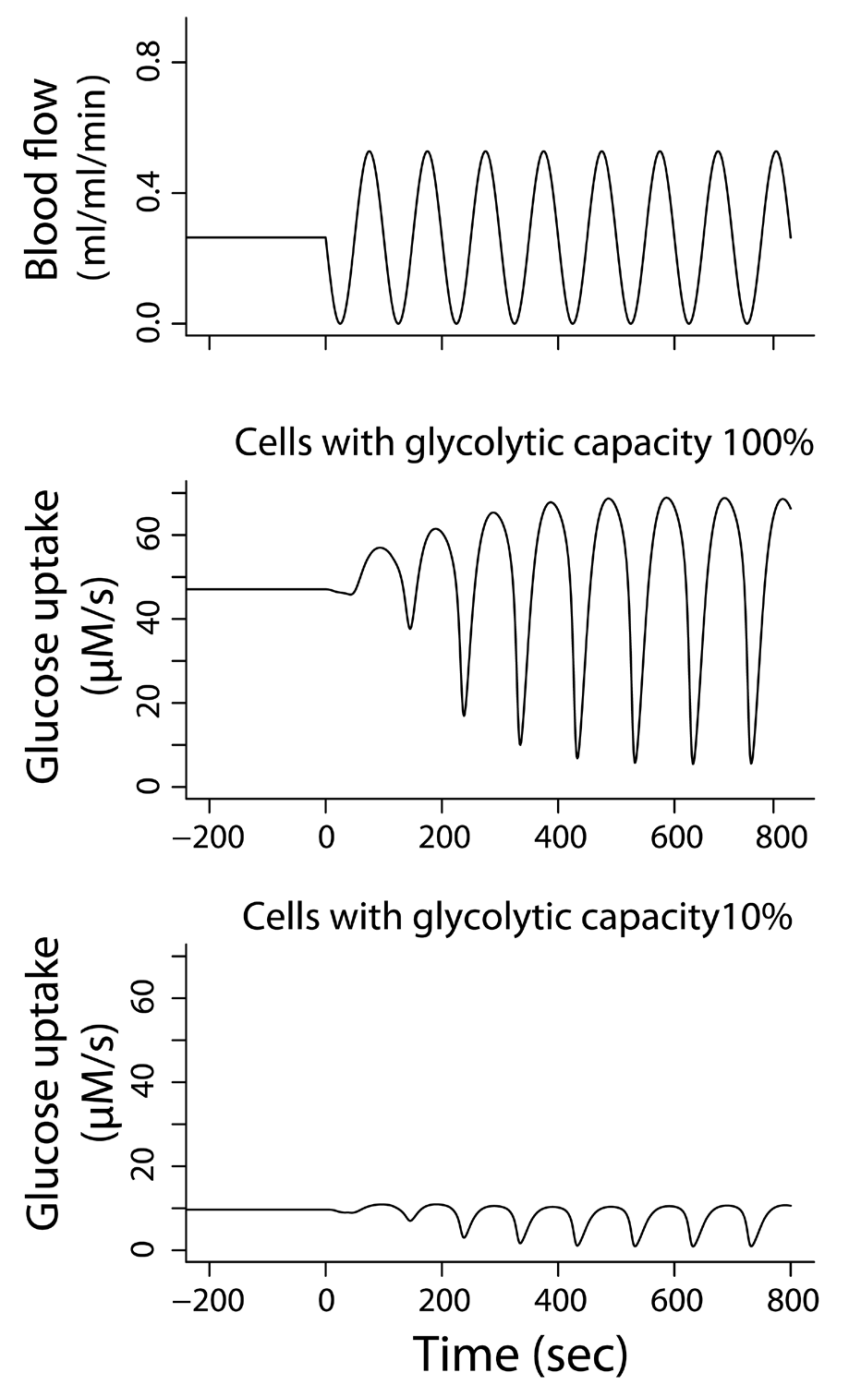
Simulation result of glucose uptake during constant and fluctuating blood flow. Same simulations as in Figure 4. Before t=0, when blood flow is constant, the cells at the full tumor glycolytic capacity show high glucose uptake (the Warburg effect), while cells with glycolytic capacity reduced to 10% show lower glucose uptake although they still excrete lactate at a higher rate than normal cells. When blood flow fluctuations start, the cells with high glycolytic capacity increase glucose uptake during the high blood flow periods and even increase average glucose uptake slightly. The cells with reduced glycolytic capacity show reduced average glucose uptake.

The cells with high glycolytic capacity are much more efficient at taking up the limited supply of glucose that becomes available during the periods of high blood flow. Figure 5 compares the simulated glucose uptake rates for the cells with high and low glycolytic capacities. When blood flow fluctuates, cells with glycolytic capacity at 100% of the EATC level manage to increase their glucose uptake during the high blood flow period to rates above the level during constant blood flow: average glucose uptake increases by about 5%. In contrast, average glucose uptake in cells with the reduced glycolytic capacity falls almost 20% during the blood flow fluctuations. The cells with high glycolytic capacity had a high lactate output while blood flow was still constant, showing the steady Warburg effect. During the blood flow fluctuations they increase their average lactate output by about 18%, and the peak of lactate output during the low flow phase is about twice the lactate excretion during constant blood flow ( Dataset 4b). The cells with low glycolytic capacity increase their lactate output during the periods with low blood flow causing anoxia, but the simulations predict that they show net uptake of lactate during the periods of high blood flow to fuel mitochondrial aerobic ATP synthesis. The high glucose uptake of the highly glycolytic tumor cells keeps the glucose concentration in tissue low for some time when blood flow starts to rise again, which makes it difficult for the cells with lower glycolytic capacity to take up glucose.

The active fraction of the glycolytic head section was ~16% when blood flow was constant in the simulation and increased to an average of ~23% during the fluctuations ( Dataset 4b). This active fraction is in between the 100% found after glucose starvation and the 7–11% when the cells were exposed to 11 mM glucose for some time ( Dataset 1, experiment 3). This illustrates the role played by the regulation of the glycolytic head section.

Although the glycolytic capacity was reduced to 10% in the low capacity glycolytic cells in the simulations in Figure 4 and Figure 5, this capacity was still higher than found in many normal cell types. ATP turnover was even well maintained at constant blood flow in cells at merely 1.5% of the tumor glycolytic capacity, which are more representative of many normal cell types ^1^ ( Supplementary Figure 3 and Dataset 4c) ^62^. However, ATP synthesis from the FBP buffer is very low and ATP turnover is strongly decreased during blood flow cycling. The storage of glucose-derived metabolites for growth is compromised in the cells with reduced glycolytic capacity. Cells at full tumor glycolytic capacity increase glucose uptake by ~8% to 50 μM/s glucose averaged over a flow cycle when blood flow starts to fluctuate in this simulation. They increase glucose uptake above the steady Warburg effect level during the high blood flow phase, in contrast to the cells with reduced glycolytic capacity. Cells at 1.5% glycolytic capacity take up only 2 μM/s glucose on average during flow fluctuations, which is ~22% lower than during constant blood flow. Tumor cells with high glycolytic capacity take much more than their fair share of glucose. The highly glycolytic cells excrete lactate at high rates during the low flow phase, reflecting high glycolytic ATP synthesis during this hypoxic episode. The peak of lactate production during the low flow phase is about twice the lactate excretion found during constant blood flow at the same average rate.

The response to cycling blood flow in Figure 4 is influenced by two homeostatic mechanisms: FBP buffering and adaptation of ATP hydrolysis. The FBP buffering mechanism on its own can still prevent the collapse of ATP during blood flow stops if ATP hydrolysis is made insensitive to the cell’s adenine nucleotide status, making regulation of ATP hydrolysis much less effective. However, even relatively small reductions of glycolytic capacity below the tumor level lead to drops in ATP concentration and ATP hydrolysis during flow stops (see Supplementary Figure 4 and Supplementary Text). PGI stores accumulated in highly glycolytic cells during periods of high blood flow are often several-fold larger than maximal tissue glucose content ( Dataset 5) ^64^, which underscores the importance of glycolytic intermediate storage for energy and nutrient buffering.

The simulations in Figure 3 – Figure 5 address conditions in tumor tissue with cycling hypoxia and nutrient shortages caused by cycling blood flow. In the next section it is considered how hypoxia and low glucose concentrations can also be caused by large diffusion distances, for instance in the ascites fluid in the murine peritoneal cavity in which the ascites cells were grown in the laboratories of Warburg ^6^, Chance ^31^, Coe ^34^ and others.

### Simulating oxygen and glucose diffusion in ascites fluid containing tumor cells

Warburg observed that glucose and O _2_ concentrations were very low in the ascites fluid in the abdomen of mice in which he was growing EATC at a high cell density ^5^; others reported ~300 µM glucose in this environment ^65– 67^. These low glucose concentrations are still sufficient for virtually maximal glucose consumption by EATC; however, glucose diffusion into the ascites fluid measured *in vivo* has limited capacity ^65^, and can provide only a small fraction of this maximal consumption. This creates a paradoxical situation: the prediction based on the average glucose concentration measured in samples of ascites fluid is that the glucose uptake of the suspended cells is high, but it is not possible that diffusion into the ascites fluid provides glucose at this rate. Simulations of EATC in ascites fluid with the present model provide an explanation for this paradox: diffusion gradients over distances of hundreds of μm cause the glucose concentration in most of the ascites fluid in the peritoneal cavity to be far below the average concentration measured in ascites fluid samples ( Dataset 6, Dataset 7) ^68, 69^. It is therefore plausible that most tumor cells in the peritoneal cavity are exposed to low glucose concentrations and consequently have very low metabolic rates. At the same time high glucose concentrations in the parts of the fluid close to the peritoneal blood vessels cause the glucose concentration to be relatively high after equilibration of the diffusion gradients in the fluid sample. Details of the calculation and results are given in the Supplementary Text.

Tumor cells in the ascites fluid shift position because of body movements and intestinal peristalsis ^67^ which leads to quick changes in O _2_ and glucose concentrations. The cells are therefore exposed to fluctuating high and low nutrient concentrations and sometimes may show a similar response as in the experiment of Figure 2 (see Supplementary Text for details). Greedy glucose uptake followed by storage of glucose-derived metabolites used to synthesize ATP in a high-capacity glycolytic system may provide selective advantages to tumor cells proliferating in environments with low and fluctuating glucose supply such as ascites fluid. This may have favored the evolution of a high-capacity dynamically regulated glycolytic system in the ascites tumor cells. Similar considerations may apply to cells in solid tumors which are also often exposed to low and fluctuating oxygen and nutrient supply.

Dataset 1. Model simulation results for 5 experiments on suspensions of Ehrlich ascites tumor cells in vitro http://dx.doi.org/10.5256/f1000research.15635.d212544
Experiment 1: low glucose concentration added (92 µM); Experiment 2: higher glucose concentration added (776 μM); Experiment 3: one hour aerobic incubation with high concentration of glucose (11.1 mM); Experiment 4: maximum FBP content following addition of a range of glucose amounts; Experiment 5: accumulation of lactate and FBP after 5 and 10 s at two low initial glucose levels; Experiments 1–5 are described in Supplementary Text: Calibrating the computational model with experimental data. Click here for additional data file. Copyright: © 2018 van Beek JHGM 2018 Data associated with the article are available under the terms of the Creative Commons Zero "No rights reserved" data waiver (CC0 1.0 Public domain dedication).

Dataset 2. Simulation results of incubation of Ehrlich ascites tumor cells at 11 mM glucose without oxygen, simulating experiments in Warburg’s laboratory http://dx.doi.org/10.5256/f1000research.15635.d212545
See description Experiment 6 in Supplementary Text: Testing the computational model with additional experimental data. Click here for additional data file. Copyright: © 2018 van Beek JHGM 2018 Data associated with the article are available under the terms of the Creative Commons Zero "No rights reserved" data waiver (CC0 1.0 Public domain dedication).

Dataset 3. Simulation results of incubation of Ehrlich ascites tumor cells in vitro with 5 mM pyruvate and 10 mM glucose http://dx.doi.org/10.5256/f1000research.15635.d212546
See description of Experiment 7 in Supplementary Text: Testing the computational model with additional experimental data. Click here for additional data file. Copyright: © 2018 van Beek JHGM 2018 Data associated with the article are available under the terms of the Creative Commons Zero "No rights reserved" data waiver (CC0 1.0 Public domain dedication).

Dataset 4. Simulations of tumor tissue including fluctuating blood flow, diffusion and tumor cell metabolism http://dx.doi.org/10.5256/f1000research.15635.d212547
ATP hydrolysis is high initially and strongly reduced when energy status is compromised. Simulation for tissue with a maximal diffusion distance of 40 µm. Result for the tissue layer at 15–20 μm from the blood vessel is given. Blood flow is constant for t≤0 and starts to fluctuate sinusoidally at t=0, periodically reaching zero for a moment, but not fully stopping.
For t ≤ 0: blood flow = offset.
For t>0: blood flow = offset - amplitude ∙ sin(2πt/Tperiod).
offset = 4.4 ml/l intracellular H2O/s, amplitude = 4.4 ml/l/s, flow ≥ 0.
Worksheet A. Simulations of tumor cells (100% of cell volume at 100% of the glycolytic capacity). From 3505–3550 sec the contribution to ATP synthesis in the tail part of glycolysis derived from falling stores of fructose 1,6-biphosphate (FBP) and other GPI is uncoupled and therefore not contributing to total ATP synthesis.
Worksheet B. Simulations of tumor cells (80% of cell volume) and a second cell type with 10% of tumor glycolytic capacity (20% of volume) in tissue with fluctuating blood flow.
Worksheet C. Simulations of tumor cells (80% of cell volume) and a second cell type with 1.5% of tumor glycolytic capacity (20% of volume) in tissue with fluctuating blood flow.
See Supplementary Text for details. Click here for additional data file. Copyright: © 2018 van Beek JHGM 2018 Data associated with the article are available under the terms of the Creative Commons Zero "No rights reserved" data waiver (CC0 1.0 Public domain dedication).

Dataset 5. Simulations of tumor tissue with metabolism, diffusion and fluctuating low blood flow with long flow stops http://dx.doi.org/10.5256/f1000research.15635.d212548
Maximal ATP hydrolysis 100 µM/s. In the second (“Glycolytic capacity 100%”) and penultimate (“FBP buffering uncoupled”) worksheet all cells had the full glycolytic capacity of tumor cells. In the rest of the simulations, 95% of cell volume is occupied by tumor cells with glycolytic capacity at 100% of tumor cell level. A second cell type with lower glycolytic capacity occupies the remaining 5% of cell volume. ATP hydrolysis responded to a fall in ATP concentration with little sensitivity (see Supplementary Text). Simulation for 8 tissue layers of width 5 μm, resulting in a maximal diffusion distance of 40 µm. Result is given for the tissue layer at 35–40 μm from the blood vessel.
Blood flow is constant for t≤0 and starts to fluctuate sinusoidally at t=0, periodically stopping fully for ~2 min; for t ≤ 0: blood flow = offset; for t>0: blood flow = offset - amplitude ∙ sin(2πt/Tperiod).
offset = 2.2 ml/l intracellular H2O/s, amplitude = 3.5 ml/l/s, flow ≥ 0.
Six different simulations with different glycolytic capacities in the second cell type are given.
Worksheet “Glycolytic capacity 100%”: all cells 100% of tumor cell level; worksheet “Glycolytic capacity 50%”: Second cell type: glycolytic capacity 50% of tumor cell level; worksheet “Glycolytic capacity 30%”: Second cell type: glycolytic capacity 30% of tumor cell level; worksheet “Glycolytic capacity 10%”: Second cell type: glycolytic capacity 10% of tumor cell level; worksheet “Glycolytic capacity 1.5%”: Second cell type: glycolytic capacity 1.5% of tumor cell level; worksheet “FBP buffering uncoupled”: Glycolytic ATP synthesis depending on falling stores of fructose 1,6-bisphosphate (FBP) and other GPI uncoupled, glycolytic capacity 100% of tumor level for all cells; worksheet “Parameters”: the parameters representing the glycolytic capacities in the simulations above. Click here for additional data file. Copyright: © 2018 van Beek JHGM 2018 Data associated with the article are available under the terms of the Creative Commons Zero "No rights reserved" data waiver (CC0 1.0 Public domain dedication).

Dataset 6. Simulation of diffusion of glucose from the peritoneum into ascites fluid not containing cells during 3 min http://dx.doi.org/10.5256/f1000research.15635.d212549
An experiment by Kemp and Mendel is simulated ^62^, see Supplementary Text. Tumor cells and metabolism were absent in this simulation. The injected ascites fluid initially contained 167 µM glucose and the time course of glucose concentrations was simulated in sixty three stacked fluid layers with an increment of 10 µm per layer. Click here for additional data file. Copyright: © 2018 van Beek JHGM 2018 Data associated with the article are available under the terms of the Creative Commons Zero "No rights reserved" data waiver (CC0 1.0 Public domain dedication).

Dataset 7. Simulation of steady-state diffusion gradients in a suspension of Ehrlich ascites tumor cells (25% vol/vol) in ascites fluid in the peritoneal cavity http://dx.doi.org/10.5256/f1000research.15635.d212550
This simulates conditions under which Erhlich ascites cells were grown in Warburg’s laboratory ^5, 6^ with a maximal diffusion distance of 630 μm from blood vessel into ascites fluid. This simulation resolves a paradox discussed by Kemp and Mendel ^62^. Sixty three layers of ascites fluid with a radius increment of 10 µm per layer were simulated. Click here for additional data file. Copyright: © 2018 van Beek JHGM 2018 Data associated with the article are available under the terms of the Creative Commons Zero "No rights reserved" data waiver (CC0 1.0 Public domain dedication).

## Discussion

The small computational model developed in this study reproduces the three effects named after Warburg, Pasteur and Crabtree, respectively, which persist for hours as long as glucose is available; at the same time the model captures the kinetic behavior in the first minutes after glucose addition and it is consistent with biochemical knowledge. This new concise model gives a testable explanation of the dynamic behavior of tumor cell metabolism. Further testing and refinement of the model and better understanding of the differential regulation of the head and tail sections of glycolysis is desirable. This requires further experimental data revealing how the duration and extent of glucose depletion and the concentrations of glycolytic intermediates affect the dynamic regulation of the glycolytic head section. Although the details of the model deserve further investigation, it already reproduces a broad range of experimental responses of ascites tumor metabolism in terms of glucose uptake, lactate production, FBP accumulation, respiration and ATP content with reasonable quantitative approximation.

The model predicts responses of tumor cells *in vitro* that can be used to test it further. One prediction is for instance that there is no initial stimulation of respiration after addition of a high concentration of glucose if a high concentration of pyruvate is simultaneously present. The full time courses of lactate production and respiration during one hour were predicted and can be experimentally checked, with low as well as high levels of pyruvate ( Dataset 3). The detailed time course of respiration to a small amount of glucose is predicted ( Figure 2A, left) but was not yet measured. The same applies to the time course of NADH levels after addition of glucose which is measurable with biochemical assays or using the fluorescent properties of NADH. Fluorescent probes of metabolite concentrations of ATP, ADP, glucose and perhaps glycolytic intermediates may provide more detailed time courses of metabolites to be compared between cell types. Measuring the phosphorylated metabolites with NMR spectroscopy may also be considered. Although the lactate production and oxygen consumption averaged over an hour after addition of a high concentration of glucose was measured in Warburg’s laboratory, different phases in the response were predicted by the model ( Dataset 1, experiment 3) and a detailed time course of the metabolic response to glucose addition can be measured. The results of inhibition of enzymes in the head or tail section of glycolysis can be predicted and measured. Metabolic responses to variations of glucose and/or oxygen concentrations with different time courses (frequencies of fluctuations, different duration of flow stops) and different amplitudes of concentrations may be used to test and refine the model further.

The model analysis indicates that the negative feedback of glycolytic intermediates on the head section of glycolysis is very important for the dynamic regulation of the system. Finding inhibitors of the feedback mechanism(s) may therefore not only be helpful to discover the regulatory pathways, but this may also have therapeutic value, especially in case these mechanisms operate exclusively in tumor cells. Blocking the negative feedback loop may lead to increased glucose uptake and accumulation of FBP and other PGI species that may be harmful to the tumor cell. Discovery of the binding sites of inhibitors may provide clues on the molecular targets and mechanisms. Genetic interventions on potential targets of the inhibitors and of downstream signaling pathways may be helpful to dissect the regulatory feedback mechanisms further.

It is tempting to speculate on the nature of the negative feedback mechanism on the head section of glycolysis. While regulation by posttranslational modification of the enzymes involved is a possibility, allosteric regulation mechanisms of head section enzymes are known and translocation of enzymes or transporters provide other potential mechanisms. Regulation at the level of the glucose transporter, by translocation or otherwise, should be considered. Allosteric inhibition of hexokinase by its product glucose 6-phosphate in tumor cells is well-known ^22, 23, 25^. In the case of EATC an inhibitory effect by fructose 1,6-bisphosphate has also been reported ^23^. Translocation of hexokinase from the mitochondrial membrane to the cytosol where it is more sensitive to inhibition by glucose 6-phosphate has been proposed as a mechanism ^24^, but is contradicted by other data ^25^. Hexokinase isoforms and their translocation between cytosol and mitochondria appear to play an important role in protection of the heart and the regulation of cardiac glycolytic metabolism during ischemia and reperfusion, a situation which shares some characteristics with cycling hypoxia in tumors ^70^. It is conceivable that the molecular mechanisms also share some similarity between these two situations. However, if the regulatory mechanisms are specific for tumor cells, they may provide attractive drug targets.

Experimental interventions in the dynamic regulation of the head section of glycolysis may be employed to test the importance of the dynamic regulatory mechanism for tumor cell proliferation and growth *in vivo*. It is conceivable that such interventions could be beneficial for the treatment of tumors by limiting the competitiveness of tumor cells against normal tissue and immune cells. Interventions may perhaps create a chronic hyperactive state of glycolysis by blocking negative feedback on the head section, accompanied by high levels of glycolytic intermediates which damage the tumor cells. It is also conceivable that blocking the reactivation of the head section when glucose levels are low may impede sufficient uptake of glucose during fluctuating low glucose supply, constituting a therapeutic intervention.

The decrease of ATP consumption, correlating with the change in adenine nucleotide pool status (ATP+ADP), is required to fit the measured data in Figure 2A. It should be noted that the decrease in ATP+ADP corresponds quantitatively with the accumulation of AMP, inosine, adenosine etc. ^46, 47^. The precise mechanism of the decrease of ATP hydrolysis requires further investigation. A useful extension of the model would be to incorporate the breakdown of ADP to AMP, inosine etc. and to analyze how this helps to maintain the free energy of ATP hydrolysis by increasing the ATP/ADP ratio under energetic stress ^46, 71^.

The model analysis suggests that a considerable fraction of glucose added to the cells after starvation is taken up in various intracellular pools within a few minutes. The intracellular destination of the carbon atoms may be determined by following isotope incorporation from labeled glucose in the intracellular pools during the transient. The high uptake of glucose in the early minutes not only serves high ATP synthesis but is also stored in the cellular biomass.

The simulations predict the metabolic responses in the tissue situation and provide a plausible and testable explanation why tumor cells benefit from a dynamically regulated uptake capacity of glucose that exceeds the capacity of the steady Warburg effect. The model predicts that tumor cells in tissue efficiently gobble up glucose, even at low extracellular concentrations, and store it for the dynamic buffering of ATP and nutrients during periods of low blood flow. The glucose uptake which leads to storage of PGI during the high blood flow phase tends to be out of phase with the increase in lactate production during the low blood flow phase which uses the PGI stores and can be upregulated about twofold relative to the steady Warburg effect level. The model suggests that the high glucose uptake capacity is kept on alert for immediate action during times of famine in case glucose becomes suddenly available. The high glycolytic capacity which underlies the glycolytic intermediate storage is partially inhibited with some delay after glucose becomes available, presumably to prevent overloading of the tumor cells with high concentrations of glucose products. Delaying the inhibition for about a minute after glucose reentry provides a time window for high uptake. A remaining question is whether this time window may be optimal for some blood flow cycling frequencies and not for others. The highly active glycolytic system in tumor cells not only provides ATP synthesis capacity to replace oxidative phosphorylation during periods of hypoxia, but additionally forms a dynamic energy and nutrient buffering system which stores glycolytic intermediates during periods of sufficient supply to be used for ATP synthesis and as a nutrient source when supply fails.

The model predictions of metabolism in tissue with fluctuating blood flow may be experimentally tested. Cancer tissue showing fluctuating blood flow may be imaged to measure tissue oxygen concentration, fluorescent probes for metabolite concentrations and NADH autofluorescence in different cell types. An implanted cancer tissue preparation as studied by Vaupel ^35^ that is supplied by a distinct large blood vessel may be perfused with blood flow controlled by a pump. After metabolic measurements *in situ,* markers of cell death or apoptosis can be compared after a period of fluctuating blood flow in comparison with a period of constant blood flow. Such tests may also be possible in cell cultures containing different cell types.

Another test is to remove glucose from the perfusate but retain lactate. The model predicts that this sustains the energy supply of tumor cells as long as flow is constant, but causes an energy crisis during cycling blood flow. A further test is to measure growth and proliferation of tumor tissue with and without inhibition of the feedback loop in case a suitable inhibitor has been identified.

The blood vessel network supplying tumor tissue usually shows a chaotic structure; blood flow and red blood cell flux in the small blood vessels are often fluctuating, often with full flow stops ^72^. It is conceivable that the tumor cells influence the organization of their blood vessel network and vascular regulation to create conditions of nutrient supply fluctuation and cycling hypoxia for which they themselves are well-equipped given their unique metabolic machinery. This would allow them to perform better than normal cells and immune cells in this environment. It is also conceivable that tumor cell metabolism plays a role in regulatory feedback loops which affect the blood vessels and cause blood flow instabilities, for instance via oxygen consumption and metabolite production.

Reperfusion after blood flow arrests and reoxygenation after a period of anoxia are often found to stimulate the generation of reactive oxygen species ^73^, which in turn can modify DNA and create genetic diversity as a basis for evolution of the cancer cells ^74^. The evolving malignant cells might thus progressively modify their environment in a direction which they are uniquely equipped to survive given their special metabolic capabilities. The same environment may be unfavorable to normal host cells and immune cells.

When tumor cells have been deprived of glucose for some time and are subsequently exposed to glucose, they can invest ~600 µM/s ATP for many seconds to sequester glucose ( Dataset 1) ^45^. For comparison, human vastus lateralis muscle consumes ~1000 µM/s for 6 s during maximal sprint performance ^75^. The high glucose uptake capacity of tumor cells tends to keep tissue glucose concentrations low during the early stages of reperfusion, making it difficult for competing cells with a lower glucose uptake capacity to take up sufficient glucose. This may be a driving force for the evolution of EATC and cells in solid tumors to a state with high and dynamically regulated glucose uptake. Cells with higher glycolytic capacity also maintain higher levels of phosphorylated glycolytic intermediates to provide building blocks for macromolecular synthesis and cell growth, in addition to their contribution to dynamic ATP buffering. The hypothesis is therefore put forward here that the nutritional and energetic buffering mediated by high-capacity glycolysis may give tumor cells a selective advantage over (tumor) cells with lower glycolytic capacity under conditions of low and fluctuating oxygen and glucose supply. The powerful glycolytic system in tumors achieves this not only by synthesizing ATP at high steady rates, but also by gobbling up scarce glucose to be stored as glycolytic intermediates to buffer temporary oxygen and nutrient shortages.

## Data availability

The data referenced by this article are under copyright with the following copyright statement: Copyright: © 2018 van Beek JHGM

Data associated with the article are available under the terms of the Creative Commons Zero "No rights reserved" data waiver (CC0 1.0 Public domain dedication). 

**Dataset 1. Model simulation results for 5 experiments on suspensions of Ehrlich ascites tumor cells *in vitro*.** Experiment 1: low glucose concentration added (92 µM); Experiment 2: higher glucose concentration added (776 μM); Experiment 3: one hour aerobic incubation with high concentration of glucose (11.1 mM); Experiment 4: maximum FBP content following addition of a range of glucose amounts; Experiment 5: accumulation of lactate and FBP after 5 and 10 s at two low initial glucose levels; Experiments 1–5 are described in Supplementary Text: Calibrating the computational model with experimental data. DOI: https://doi.org/10.5256/f1000research.15635.d212544 ^45^.

**Dataset 2. Simulation results of incubation of Ehrlich ascites tumor cells at 11 mM glucose without oxygen, simulating experiments in Warburg’s laboratory.** See description Experiment 6 in Supplementary Text: Testing the computational model with additional experimental data. DOI: https://doi.org/10.5256/f1000research.15635.d212545 ^48^.

**Dataset 3. Simulation results of incubation of Ehrlich ascites tumor cells *in vitro* with 5 mM pyruvate and 10 mM glucose**. See description of Experiment 7 in Supplementary Text: Testing the computational model with additional experimental data. DOI: https://doi.org/10.5256/f1000research.15635.d212546 ^50^.

**Dataset 4. Simulations of tumor tissue including fluctuating blood flow, diffusion and tumor cell metabolism.** ATP hydrolysis is high initially and strongly reduced when energy status is compromised. Simulation for tissue with a maximal diffusion distance of 40 µm. Result for the tissue layer at 15–20 μm from the blood vessel is given. Blood flow is constant for t≤0 and starts to fluctuate sinusoidally at t=0, periodically reaching zero for a moment, but not fully stopping.

For t ≤ 0: blood flow = offset.

For t>0: blood flow = offset - amplitude ∙ sin(2πt/T _period_).

offset = 4.4 ml/l intracellular H _2_O/s, amplitude = 4.4 ml/l/s, flow ≥ 0.

Worksheet A. Simulations of tumor cells (100% of cell volume at 100% of the glycolytic capacity). From 3505–3550 sec the contribution to ATP synthesis in the tail part of glycolysis derived from falling stores of fructose 1,6-biphosphate (FBP) and other GPI is uncoupled and therefore not contributing to total ATP synthesis.

Worksheet B. Simulations of tumor cells (80% of cell volume) and a second cell type with 10% of tumor glycolytic capacity (20% of volume) in tissue with fluctuating blood flow.

Worksheet C. Simulations of tumor cells (80% of cell volume) and a second cell type with 1.5% of tumor glycolytic capacity (20% of volume) in tissue with fluctuating blood flow.

See Supplementary Text for details. DOI: https://doi.org/10.5256/f1000research.15635.d212547 ^62^.

**Dataset 5. Simulations of tumor tissue with metabolism, diffusion and fluctuating low blood flow with long flow stops.** Maximal ATP hydrolysis 100 µM/s. In the second (“Glycolytic capacity 100%”) and penultimate (“FBP buffering uncoupled”) worksheet all cells had the full glycolytic capacity of tumor cells. In the rest of the simulations, 95% of cell volume is occupied by tumor cells with glycolytic capacity at 100% of tumor cell level. A second cell type with lower glycolytic capacity occupies the remaining 5% of cell volume. ATP hydrolysis responded to a fall in ATP concentration with little sensitivity (see Supplementary Text). Simulation for 8 tissue layers of width 5 μm, resulting in a maximal diffusion distance of 40 µm. Result is given for the tissue layer at 35–40 μm from the blood vessel.

Blood flow is constant for t≤0 and starts to fluctuate sinusoidally at t=0, periodically stopping fully for ~2 min; for t ≤ 0: blood flow = offset; for t>0: blood flow = offset - amplitude ∙ sin(2πt/Tperiod).

offset = 2.2 ml/l intracellular H _2_O/s, amplitude = 3.5 ml/l/s, flow ≥ 0.

Six different simulations with different glycolytic capacities in the second cell type are given.

Worksheet “Glycolytic capacity 100%”: all cells 100% of tumor cell level; worksheet “Glycolytic capacity 50%”: Second cell type: glycolytic capacity 50% of tumor cell level; worksheet “Glycolytic capacity 30%”: Second cell type: glycolytic capacity 30% of tumor cell level; worksheet “Glycolytic capacity 10%”: Second cell type: glycolytic capacity 10% of tumor cell level; worksheet “Glycolytic capacity 1.5%”: Second cell type: glycolytic capacity 1.5% of tumor cell level; worksheet “FBP buffering uncoupled”: Glycolytic ATP synthesis depending on falling stores of fructose 1,6-biphosphate (FBP) and other GPI uncoupled, glycolytic capacity 100% of tumor level for all cells; worksheet “Parameters”: the parameters representing the glycolytic capacities in the simulations above. DOI: https://doi.org/10.5256/f1000research.15635.d212548 ^64^.

**Dataset 6. Simulation of diffusion of glucose from the peritoneum into ascites fluid not containing cells during 3 min.** An experiment by Kemp and Mendel is simulated ^65^, see Supplementary Text. Tumor cells and metabolism were absent in this simulation. The injected ascites fluid initially contained 167 µM glucose and the time course of glucose concentrations was simulated in sixty three stacked fluid layers with an increment of 10 µm per layer. DOI: https://doi.org/10.5256/f1000research.15635.d212549 ^68^.

**Dataset 7. Simulation of steady-state diffusion gradients in a suspension of Ehrlich ascites tumor cells (25% vol/vol) in ascites fluid in the peritoneal cavity.** This simulates conditions under which Erhlich ascites cells were grown in Warburg’s laboratory ^5, 6^ with a maximal diffusion distance of 630 μm from blood vessel into ascites fluid. This simulation resolves a paradox discussed by Kemp and Mendel ^65^. Sixty three layers of ascites fluid with a radius increment of 10 µm per layer were simulated. DOI: https://doi.org/10.5256/f1000research.15635.d212550 ^69^.

## Software availability

**Source code available from:** https://github.com/jhvanbeek/Metabolic-model-DSWE.

**Archived source code at time of publication:** http://dx.doi.org/10.5281/zenodo.1322391 ^76^.

**License:** GNU General Public License v3.0.

## Supplementary materials

**Supplementary Text. Description of the computational model.** This file includes kinetic rate equations and differential equations for metabolite concentration changes with detailed description. In addition, it contains a description of the transport equations for oxygen and metabolites used for simulation of metabolism in tissue with cycling blood flow; also included are descriptions of computational methods, parameter optimization, analysis of prediction uncertainty, and of the experimental data sets used to calibrate and test the model. Finally it contains a description of simulation of cells growing in ascites fluid.

**Supplementary Figure 1. Uncertainty in prediction of the simulation results for tissue with cycling blood flow.** The simulation in Figure 4 was repeated with parameter sets from 45 MCMC runs replicated with independent random seeds. The lines give the median (black) and the estimated 5 and 95% quantiles (red) of the prediction. This reveals the uncertainty in the model prediction of Figure 4. Model simulations are for tissue during blood flow cycling. Eighty percent of the cells had the full tumor glycolytic capacity (left column); 20% of the cell volume had 10% of the full glycolytic capacity (right column). The two top rows show tissue conditions experienced by both cell types. Blood flow was initially constant at 0.264 ml/ml intracellular H _2_O/min. For t>0 a sinus wave with amplitude 0.264 ml/ml/min was superimposed. High ATP consumption, >160 μM/s, was maintained at constant blood flow. When blood flow started cycling at t=0, ATP synthesis from the decline in fructose 1,6-bisphosphate (FBP) and other phosphorylated glycolytic intermediates (bottom row) maintained ATP levels and ATP hydrolysis rates in cells with full tumor glycolytic capacity. However, there was a sharp drop in ATP levels and hydrolysis rate in the cells with reduced glycolytic capacity. The 5% and 95% quantiles show that the predicted response pattern is reproducible for a broad range of parameter sets which reflect the potential experimental variation in the data used for parameter optimization.

**Supplementary Figure 2. Variation in simulation results for the *in vitro* experiments (see Figure 2A).** The time courses were calculated using the final parameter sets from 45 MCMC ensembles, replicated with independent random seeds (same as in Supplementary Figure 1). All 45 results for individual parameter sets were plotted (blue lines). The time courses for the distinct parameters sets correspond within a narrow range.

**Supplementary Figure 3. Simulation with cells at low glycolytic capacity during the start of cycling blood flow.** Simulation as in Figure 4, but with 20% of the cells at 1.5% (rather than 10%) of the full glycolytic capacity. Model simulations of tissue during cycling blood flow: 80% of the cell volume had the full tumor glycolytic capacity; 20% of the cells had 1.5% of the full capacity, representative of many normal body cell types. The two top rows show tissue conditions experienced by both cell types. Blood flow was constant at 0.264 ml/ml intracellular H _2_O/min before t=0. For t>0 a sinus wave with amplitude 0.264 ml/ml intracellular H _2_O/min was superimposed. High ATP consumption, >160 μM/s, was maintained at constant blood flow. Concentrations and fluxes at ~18 μm from the microvessel are given. See the legend of Figure 3 for description of ATP synthesis fluxes. When blood flow started to fluctuate, ATP synthesis from the decline in fructose 1,6-bisphosphate (FBP) and other phosphorylated glycolytic intermediates (red curve) maintained ATP levels and high ATP hydrolysis rates in cells with full tumor glycolytic capacity. By contrast, glycolytic fluxes were low and there was a sharp drop in ATP levels in the cells with reduced glycolytic capacity.

**Supplementary Figure 4. Simulations of tumor metabolism during cycling and stopping blood flow.** In the simulation on the second row and the bottom row all cells had the full tumor glycolytic capacity. In the third through penultimate row ATP concentrations and fluxes are given for cells which had their glycolytic capacity changed to a percentage of the full tumor cell glycolytic capacity, as indicated above the rows. These cells with reduced glycolytic capacity constitute 5% of the total cell volume; the remaining 95% of the cells had the full tumor glycolytic capacity (100%). Results are calculated at ~38 μm from the blood vessel. Blood flow and fluxes of O _2_ and glucose carried into the tissue by the arterial blood are common to all simulations (top row); the other rows each represent a separate simulation. Blood flow was initially constant and started to fluctuate at t=0, including flow stops. All ATP fluxes and blood flow are expressed per unit volume of intracellular H _2_O ATP hydrolysis responded to a fall in ATP concentration with little sensitivity (see Supplementary Text). The ATP synthesis flux is partitioned in oxidative phosphorylation (blue) and two contributions by the tail section of glycolysis, with fructose 1,6-bisphospate (FBP) either directly fed from the head part of glycolysis (direct glycolytic throughput: dashed line) or taken from decreasing FBP stores (FBP buffering: red line). When ATP synthesis depending on phosphorylated glycolytic intermediate stores was uncoupled (bottom row), ATP levels collapsed during flow stops, although glycolytic capacity was 100% in all cells.

**Supplementary Table 1. State variables in the model of tumor cell metabolism.**

**Supplementary Table 2. Metabolic fluxes in the model of tumor cell metabolism.**

**Supplementary Table 3. Parameters in the model of tumor cell metabolism.**
