# Supplementary material for: The dynamic side of the Warburg effect: glycolytic intermediate storage as buffer for fluctuating glucose and O 2 supply in tumor cells
Source: F1000Res. 2018 Dec 28;7:1177. Originally published 2018 Aug 2. [Version 2] doi: 10.12688/f1000research.15635.2 (PMC6352925; doi:10.12688/f1000research.15635.2)
Supplement: Supplementary file 8 [file f1000research-7-18800-s0014.tgz › 7ca19dce-dbd1-45cd-b1b2-43bff59de81b_Revision_Supplementary_Text.docx]

**Supplementary Text**

**for “The Dynamic Side of the Warburg Effect: Glycolytic Intermediate Storage as Buffer for Fluctuating Glucose and O_2_ Supply in Tumor Cells” by JHGM van Beek**

Short Description of the Computational Model

The design of the model is based on two considerations:

1. the availability of data on transient responses to glucose in Ehrlich ascites tumor cells (EATC) on key nodes in metabolism: glucose uptake, fructose 1,6-bisphosphate as main representative of the glycolytic intermediates, lactate production, oxygen uptake and ATP concentration. The scheme of the model in Figure 1 indicates that the model’s level of coarse-graining fits these key nodes.

2. the lack of knowledge on specific enzyme kinetics and regulation of the complete set of enzymes in the glycolytic chain in EATC. The complex mitochondrial metabolism has also not been described in detail for EATC, but can be adequately replaced by a well-known overall kinetic equation. The dependence of ATP hydrolysis on the energetic status of the cell can be represented by a coarse-grained phenomenological description representing the many ATP consuming processes in the cell. The pyruvate-to-lactate reaction by lactate dehydrogenase is well known, and data on the pyruvate node are therefore dispensable.

As a consequence of these considerations glycolysis was conceptually divided in a head and tail section, an approach already taken in early conceptual models of glycolysis, e.g. by Coe and Greenhouse (*1*). Each section is represented by an overall kinetic equation. The approach to model glycolysis in this manuscript retains biochemical knowledge without incorporating all the process details. For instance, the head section of glycolysis is determined by ATP and glucose concentration as known from the kinetics of hexokinase, which is an important controlling step in this section. The rate equations of the glycolytic head and tail sections were not parameterized prior to integrating them in the model. The consideration was that no data are available for the isolated head section or isolated tail section. If the enzymes for both these sections were isolated, their behavior would likely be affected by the isolation procedure and their isolated state. The behavior of head or tail section are derived from the data on the intact cell, where the input and output of the head section is for instance well represented in the data by its measured input (glucose uptake) and output (FBP). The parameters for the equations for head and tail sections are therefore derived by calibration with the measured whole-system responses.

Chance and Hess explained their kinetic data on Ehrlich ascites tumor cells (EATC) with the first computational model of a multi-enzyme system ever published (*2, 3*). Their model is of great historical interest, but incorporated biochemical assumptions which are now considered untenable. A new computational model is developed here which simultaneously describes Warburg’s measurements on steady EATC metabolism (*4*) and kinetic data during the first minutes after reintroducing glucose by Chance (*2, 5, 6*), Coe (*7, 8*) and others (*9, 10*). The approach is similar to recent modeling of yeast glycolysis which aimed to show trade-offs between robustness and efficiency under non-limiting glucose supply (*11*). The new model gives a simplified representation of the EATC metabolic system with the aim to reproduce a broad range of metabolic responses of EATC with reasonable quantitative approximation.

Blood flow in tumor tissue is often low and fluctuating, resulting in cycling hypoxia (*12-14*) and glucose shortages. The present model therefore includes the dependence of glycolysis and mitochondrial ATP production on glucose and oxygen concentrations. Glycolysis is represented by a head section which produces phosphorylated glycolytic intermediates (PGI) from glucose and ATP. The major constituent of PGI is fructose 1,6-bisphosphate (FBP), which is measured in the modeled experiments. In the glycolytic tail section, downstream from FBP, ADP is phosphorylated to ATP (Figure 1). Inhibition of the head section by PGI constitutes an important regulatory feedback loop. Strong negative feedback by glucose 6-phosphate on hexokinase in tumor cells (*15*) and by FBP on the head section of glycolysis in EATC (*16*) is well known. This inhibition shows >10 s time delay (*16, 17*).

Model Equations

Kinetic Rate Equations

J_head_, the flux in the head section of glycolysis where glucose is twice phosphorylated, is quantified as the amount of six-carbon units taken up per liter intracellular water per second (µM/s). The reactants of the head section are glucose and 2 ATP molecules; the products of the head section are fructose 1,6-bisphosphate (FBP) and 2 ADP. The head section consists of glucose transport across the cell membrane and of the hexokinase, glucose 6-phosphate isomerase and phosphofructokinase enzymes, which catalyze the double phosphorylation of hexose to FBP. The rate equation resembles the equation for hexokinase (*18, 19*), an enzyme which exerts major flux control over glycosis in cancer cells (*15*). There is strong feedback inhibition of hexokinase by glucose 6-phosphate (*15*). Feedback inhibition of this enzyme by FBP was also described specifically for EATC (*16, 20*). The third enzyme in the head section, phosphofructokinase (PFK), is inhibited in EATC by phosphoenolpyruvate (PEP), another PGI species (*21*). These inhibition mechanisms of the head section by downstream metabolites are implemented in the present model by modulation of the fraction of the head section which is active, F_active_. This is determined by FBP, representing the PGI pool, via forward and backward rate constants, see Eq. 22. The rate equation therefore describes the effects of glucose and ATP concentrations and feedback inhibition from the PGI pool:

$J_{head}=V_{max,head}\cdot F_{active} \cdot\frac{\frac{ATP}{K_{ATP,head} } \frac{glucose}{K_{glucose,head}}}{1+ \frac{ATP}{\begin{matrix} K_{ATP,head} \\ \end{matrix}} + \frac{glucose}{K_{glucose,head}} +\frac{ATP }{K_{ATP,head} } \cdot\frac{glucose}{K_{glucose,head}} }$ (1)

Variables and parameters in all equations are described in Supplementary Tables 1-3.

In the tail section of glycolysis FBP, inorganic phosphate (P_i_), nicotinamide adenine dinucleotide (NAD) and ADP are used as reactants; pyruvate, ATP and NADH are produced with flux J_tail_. The flux unit here is amount of three-carbon units per liter intracellular water per second (µM/s). The tail section consists of the enzymes glyceraldehyde 3-phosphate dehydrogenase (GAPDH), phosphoglycerate kinase, phosphoglycerate mutase, enolase and pyruvate kinase. Also in this case the complex kinetics of several enzymes in the tail part is represented by an approximation containing a limited number of parameters that can be estimated from the experimental data on the intact system. The model equation for the tail section resembles the kinetics of GAPDH, which presumably is an important site of flux control in the tail part of glycolysis (*22, 23*), in particular in EATC (*24*). A factor in equation 2 reflects that NADH is an inhibitor of GAPDH in EATC (*24*); high NADH levels will also increase reverse flux in GAPDH (*18, 25*). An additional factor depending on ADP reflects its participation in the phosphoglycerate kinase and pyruvate kinase reactions.

The rate equation depends on FBP, ADP, NAD^+^, each represented in the equation by a factor with a Michaelis-Menten type constant to yield saturable enzyme kinetics. The rate is assumed to be saturated with P_i_ (see discussion below). NADH, which is a product of the GAPDH reaction, negatively affects the forward net reaction rate in the tail section moderated by an inhibition constant (*24*). The factor depending on FBP reflects the saturable dependence of the tail part on phosphorylated substrate as well as allosteric activation of the tail part which has been reported for pyruvate kinase (*26, 27*). To represent the potential double effect of FBP, as substrate and as allosteric activator, different forms of the equation below were tried with FBP concentration to a power (Hill coefficient), but this did not lead to better fits to the data. Note in the results that K_FBP,tail_ is estimated to be very low and is exceeded by the FBP concentration very fast. Consequently, FBP’s effect as substrate and activator of the glycolytic tail section appears to be adequately represented by the equation.

$J_{tail}=V_{max, tail} \frac{FBP}{K_{FBP,tail}+FBP} \frac{NAD}{K_{NAD,tail}+NAD} \frac{ADP}{K_{ADP,tail}+ADP}\frac{K_{i,NADH}}{K_{i,NADH,tail}+NADH}$ (2)

The possible influence of reverse fluxes in the glycolytic chain was investigated in two detailed mathematical models and appears to be negligible (see below). In order to reduce the present model as much as possible, the reverse reactions in glycolysis were therefore neglected.

Mitochondrial ATP synthesis by oxidative phosphorylation depends on ADP, P_i_ and O_2_. The reaction is strongly irreversible. The functional relation in Equation 3 is used to represent the dependence on substrate concentrations (*28-30*):

|  |  |
| --- | --- |

$J_{mit}= V_{synmax} \frac{ \frac{ADP}{K_{ADP,mit}} \cdot\frac{P_{i}}{K_{Pi,mit}}}{1+ \frac{ADP}{K_{ADP,mit}}+\frac{P_{i}}{K_{Pi,mit}}+\frac{ADP}{K_{ADP,mit}}\cdot\frac{P_{i}}{K_{Pi,mit}} }\cdot\frac{O_{2}}{K_{O_{2},mit}+ O_{2}}$ (3)

The sensitivity of respiration in EATC to low oxygen concentrations has been determined by Froese (*29*). The K_O2,mit_ was found to be 0.26 μM based on measured O_2_ concentrations in the environment of the cell. This shows that the oxygen concentration gradient between environment and cell interior is very small.

Although P_i_ may play a regulatory role in EATC metabolism if external phosphate concentrations are low, changes in metabolism are saturated at high external phosphate concentrations and regulation is shifted to ADP (*3, 6, 31, 32*). The experiments simulated here were conducted at high extracellular inorganic phosphate concentrations (*7, 33*), associated with high intracellular phosphate concentrations (*34, 35*). Measured intracellular P_i_ levels are well above the Km’s of oxidative phosphorylation (*3, 30*) and glyceraldehyde 3-phosphate dehydrogenase (*32*). Metabolic regulation is therefore saturated with phosphate at these high phosphate concentrations in EATC (*31*). Variation of P_i_ and its effect on metabolic regulation can therefore be neglected, avoiding unnecessary complexity of the model: intracellular inorganic phosphate concentration was therefore fixed at 6000 μM in the model, close to saturating for regulation of oxidative phosphorylation and glycolysis.

Lactate dehydrogenase catalyzes a reversible reaction, pyruvate + NADH ⇌ lactate + NAD, depending on reactant and product concentrations. The rate equation is taken from Lambeth and Kushmerick, taking the reverse reaction and thermodynamic constraints into account (*25*). This allows for lactate uptake for aerobic metabolism and lactate production from aerobic glycolysis :

|  |  |  |
| --- | --- | --- |

$J_{lac}=\frac{V_{max,ldh,for }\frac{pyr}{K_{pyr,ldh}} \frac{NADH}{K_{NADH,ldh}} - \frac{V_{max,ldh,for }}{K_{eq,ldh}}\frac{K_{lac,ldh}K_{NAD,ldh}}{K_{pyr,ldh}K_{NADH,ldh}}\frac{lac}{K_{lac,ldh}} \frac{NAD}{K_{NAD,ldh}} }{1+\frac{pyr}{K_{pyr,ldh}}+ \frac{NADH}{K_{NADH,ldh}} + \frac{pyr}{K_{pyr,ldh}} \frac{NADH}{K_{NADH,ldh}}+\frac{lac}{K_{lac,ldh}} + \frac{NAD}{K_{NAD,ldh}} + \frac{lac}{K_{lac,ldh}} \frac{NAD}{K_{NAD,ldh}}}$ (4)

This equation is of the same form as the random Bi-Bi reversible Michaelis-Menten equation for lactate dehydrogenase in the model of Marin-Hernandez et al. (*18*).

In order to explain the time course of the measured metabolites that carry phosphate groups following glucose addition *in vitro*, a strong decrease of ATP hydrolysis, J_hyd,ATP_, proves essential. This decrease mainly affects processes such as macromolecular synthesis and degradation, ion pumping, etc. (*36*). The decline in J_hyd,ATP_ is clearly revealed by calculating the mass balance of phosphate metabolites directly from the experimental data (see Figure 2A). This decline is assumed to result from decreased availability of energy-delivering adenine nucleotides. The highest correlation of falling ATP hydrolysis is not with ATP or the ratio ATP/ADP, but with the sum AdN = ATP + ADP of double and triple phosphorylated adenine nucleotides. The decrease in AdN in turn corresponds with the sum of accumulated breakdown products of ATP and ADP such as AMP, inosine, adenosine and hypoxanthine (*37, 38*). ATP_initial_ and ADP_initial_ are the initial ATP and ADP concentrations in the fully energized state before the intervention. AdN_initial_ = ATP_initial_ + ADP_initial_. In the model, J_hyd,ATP_ is therefore declining linearly with the adenine nucleotide concentration:

$J_{hyd,ATP}= V_{max,hyd} - k_{hyd,AdN} (AdN_{initial}-AdN) for AdN\geq AdN_{cutoff}$ (5)

In the *in vitro* experiments (Figure 2), AdN reaches a minimum value, AdN_cutoff_ , which is used as a threshold in further simulations (see below). Different cellular processes have a quite different sensitivity to lowering of energy status, ATP and/or AdN levels. Protein synthesis is very sensitive to declining ATP content with a 20% decline causing ~60% decline in ATP hydrolysis (*39*), similar as the energy dependence of ATP hydrolysis determined in the *in vitro* experiments of Figure 2. Indeed, protein synthesis is the most active ATP-consuming process in EATC (*36*). In contrast, the sensitivity of protein degradation is relatively low with a 70% fall in ATP content causing a ≤ 30% decrease in protein breakdown (*39*,*40*). The sensitivity of ion pumps to ATP is between these extremes (*40*).

When AdN falls below AdN_cutoff_ in simulations of low nutrient and O_2_ supply conditions in tissue, V_hyd_ is given by a curve continuous with Eq. 5:

$J_{hyd,ATP}=k_{hyd,AdN,low} AdN+C_{power}AdN^{\gamma} for AdN<AdN_{cutoff}$ (6)

The linear term with k_hyd,AdN,low_ = 0.0098704 s^-1^ defines a sensitivity which is similar as found for protein synthesis at low energy status below AdN­_cutoff_ (*39*). The linear relation at low AdN is joined to the linear relation at high AdN in Eq. 5 via the power term in Eq. 6 which has a high value for γ so that is has a negligible effect on the linear relation at low AdN. The coefficient for the power term, C­_power_, and the power γ are determined such that Eqs. 5 and 6 together form a smooth function which is continuous and differentiable at AdN = AdN_cutoff_.

For the simulations in Figures 3-5 and Supplementary Figure 3 a high k_hyd,AdN_ was chosen for the upper part of the curve in Eq. 5 (Supplementary Table 3), reflecting a strong decrease of J_hyd,ATP_ if AdN falls. This is the value determined in the *in vitro* experiment of Figure 2. Eqs. 5 and 6 together define a “hockeystick” curve similar to the energy dependence of protein synthesis in Fig 8 of Gronostajski et al. (*39*), with slope increasing with AdN and becoming constant above AdN_cutoff_. By using this strong reduction of ATP hydrolysis with AdN, the energy homeostatic mechanism of reduction of ATP hydrolysis is very active in these tissue simulations. Eqs. 5 and 6 can jointly define convex or concave curves similar as found experimentally for various ATP consuming processes (*39*). For the simulations in Supplementary Figure 4 a much lower sensitivity of ATP hydrolysis to energy status was chosen, resembling the energy dependence of protein hydrolysis (see details below).

Eq. 7 represents ADP breakdown to AMP when the energy status decreases (*37,38*). AMP is broken down further to adenosine, inosine, hypoxanthine etc. Two ADP molecules yield AMP in the adenylate kinase reaction (2 ADP ⇌ ATP + AMP), giving a quadratic relation between AMP production and ADP concentration (*41*):

 (7)

In long experiments there is significant resynthesis of ADP, J­_synth,ADP_, accompanied by reuptake of adenosine, inosine, hypoxanthine etc. (*37, 38*), phenomenologically represented by :

 (8)

with AdN_initial­_ set as the target level for resynthesis.

Intracellular storage of carbon metabolites, glc_store_, coming from glucose via the glycolytic intermediate pool and used for synthesis and growth, occurs with rate J_store_ (with as unit amount of 6-carbon equivalents per liter intracellular water per second):

 (9)

Incorporation of glucose derived carbon in intracellular metabolite pools and biomass is appreciable and is required to close the mass balance of measured metabolites (Figure 2A). J_store_ may be assumed to be proportional to the rate of cell mass increase during growth. J_store_ = 10 μM/s corresponds to a growth rate of 0.03 g glucose incorporated in the metabolite pool per gram dry mass of the cell per hour.

|  |  | percent |
| --- | --- | --- |

Algebraic Relations

Net ATP synthesis by glycolysis, J_ATP,glyc_, is the ATP synthesis by the tail section minus the ATP consumption by the head section:

$J_{ATP, glyc}=2J_{tail }-2J_{head}$ (10)

Note that J­­_head_ is given in 6-carbon units and J_tail­­_ in 3-carbon units. This takes the stoichiometry of the overall reaction of the head section into account: glucose + 2 ATP -> fructose 1,6-bisphosphate + 2 ADP, while 2 ATP are synthesized per 3-carbon unit processed in the tail section.

The total amount of NAD + NADH, NAD_tot_, is assumed constant in the model and NADH concentration is calculated from the NAD concentration.

$NADH=NADH_{tot}-NAD$ (11)

Oxygen consumption, J_O2_, is related to the mitochondrial ATP synthesis by the P/O_2_ ratio:

$J_{O_{2}}=\frac{J_{mit}}{ratio_{P/O_{2}}}$ (12)

Three O_2_ molecules correspond stoichiometrically to oxidative metabolism of one lactate molecule. Three O_2_ also correspond to the combination of pyruvate and NADH + H^+^ which is produced in a one-to-one ratio either in the tail section of glycolysis or from lactate. The mitochondria are assumed to consume pyruvate at rate J_pyr,mit_ in stoichiometric ratio to their O_2_ consumption:

$J_{pyr,mit}=\frac{J_{O_{2}}}{3}$ (13)

The mitochondria consume NADH + H^+^ at rate J_nadh,mit_ via redox shuttling(*42*) and produce nicotinamide adenine dinucleotide (NAD):

$J_{nadh,mit}= \frac{J_{O_{2}}}{3}$ (14)

Oxidation of the pyruvate molecule by itself corresponds stoichiometrically to 2 ½ molecule O_2_ and NADH+H^+^ to ½ molecule O_2_. The factor 3 in eqs. 13 and 14, however, reflects the simplifying assumption that the mitochondria metabolize one NADH per pyruvate taken up.

FBP represents PGI in the model; this is efficient because FBP normally forms the vast majority of PGI and model results can also be directly compared to FBP measurements. The total PGI content has a fixed ratio, R_PGI/FBP_, to the FBP content in the model (*6, 7, 33*) :

 (15)

In the mass balance of glucose-derived metabolites the complete PGI content is taken into account.

Differential Equations

Glucose is used by the head section of glycolysis and is assumed to be distributed at equal concentrations in the cytosolic and extracellular (interstitial) volumes of distribution :

$\frac{d\left[glucose\right]}{dt}= \frac{- J_{head}}{V_{ext}+ V_{cyt}}$ (16)

V_cyt_ is cytosolic water volume as fraction of the total intracellular water volume; V_ext_ is the ratio of extracellular water volume to total intracellular water volume. Eq. 16 gives the rate of change of glucose concentration in cytosolic and extracellular water. Note that concentrations are given in mol per liter H_2_O in the respective compartments (for glucose the cytosol and extracellular volume) and metabolic fluxes J are in mol per liter total intracellular H_2_O per s. Expression of flux per volume of total cell water provides a common reference.

The assumption of equal concentrations in cytosolic and extracellular space requires further consideration. The transport capacity of the EATC cell membrane for glucose is high. Saha and Coe (*43*) found two transport systems: a high affinity one with K_m_=100 µM and V_max_ = 467 µM/s and a low affinity one with K_m_=25 mM and V_max_ = 3333 µM/s. To investigate the effect of transport limitation on model performance, the equation for glucose permeation of the cell membrane in EATC was added to the model and parameter optimization repeated, keeping the glucose transport parameters fixed to the measured values. The fit of the model was virtually indiscernible from that in Fig. 2A and the cost function value was even slightly lower. Changes in parameter values were small: the capacity of the glycolytic head section, V_max,head_, was for instance 394 μM/s without the separate glucose transport equations (see Supplementary Table 3), and 376 μM/s with the additional glucose transport equations. However, there was one exception: the K_glucose,head_ which represents the apparent affinity of the head section for glucose fell from 51 μM to 4.9 μM, and the calculated glucose concentrations in the cytosol were decreased relative to the extracellular space. Given the similarity of the results and to keep the model as simple as possible, in the rest of the present article glucose transport was considered to be part of the head section of glycolysis and the estimated parameters for the head section (Eq. 1) consequently include the effect of glucose transport.

Lactate is produced from pyruvate and NADH by the lactate dehydrogenase reaction and distributed in the cytosolic and extracellular space:

$\frac{d\left[lac\right]}{dt}=\frac{ J_{lac}}{V_{ext}+ V_{cyt}}$ (17)

The reaction is reversible and, with J_lac_ negative, lactate and NAD can be converted to pyruvate and NADH and used for mitochondrial metabolism.

Pyruvate is produced by glycolysis and used for lactate production and mitochondrial metabolism:

$\frac{d\left[pyr\right]}{dt}=\frac{J_{tail}- J_{lac}-J_{pyr,mit}}{V_{ext}+V_{cyt}}$ (18)

Lactate and pyruvate are distributed intracellularly and extracellularly at equal concentration to keep the model as simple as possible. Lactate and pyruvate are transported via highly active transporters, which suggests that this is a reasonable approximation: lactate transport across the cell wall of EATC is characterized by a V_max_ of 1866 µM/s with K_m_=4.7 mM and pyruvate transport is characterized by a V_max_ of 1290 µM/s with K_m_=8.5 mM (*44*).

The time-dependent ATP balance is the result of the net glycolytic ATP synthesis rate (see Eq. 10), mitochondrial ATP synthesis (J_mit_) and hydrolysis for cellular processes and growth (J_hyd_,ATP):

$\frac{d\left[ATP\right]}{dt}=\frac{J_{ATP,glyc}+ J_{mit}- J_{hyd,ATP}}{V_{cyt}}$ (19)

ATP, like other phosphorylated metabolites such as ADP, FBP and other PGI, does not cross the cell membrane and is distributed intracellularly only.

In addition to ADP’s involvement in ATP usage and resynthesis, ADP is also broken down at rate J_breakdown,ADP_ to AMP, adenosine, inosine, hypoxanthine, etc., and resynthesized at rate J_synth,ADP_ (*37,38*):

$\frac{d\left[ADP\right]}{dt}=\frac{-J_{ATP,glyc}-J_{mit}+J_{hyd,ATP}- J_{breakdown,ADP}+ J_{synth,ADP}}{V_{cyt}}$ (20)

The fructose 1,6-bisphosphate (FBP) concentration represents the total phosphorylated glycolytic intermediate pool, of which it forms the major part. FBP rather than PGI is used as state variable because it is directly measured in the experimental data sets. FBP is related to PGI by a constant ratio (Eq. 15). FBP’s rate of change is the result of synthesis by the head section of glycolysis, consumption by the tail section of glycolysis and uptake of glycolytic intermediates in the cell in various molecular forms for storage and growth:

 (21)

The balance of the fluxes gives the rate of change of the total PGI pool; division by R_PGI/FBP_ results in the rate of change of FBP. The factor ½ before J_tail_ takes into account that J­­_tail­_ represents flux of 3-carbon units while FBP contains 6 carbon atoms.

The head section of glycolysis is inhibited by signals from the phosphorylated glycolytic intermediate pool (see description of Eq. 1 above), represented by FBP in the model. The active fraction of the head section is denoted by F_active_.

$\frac{dF_{active}}{dt}= k_{b}\left(1-F_{active}\right)- k_{f}\left[FBP\right]F_{active}$ (22)

The second order forward rate constant for inactivation, k­_f_, and the backward rate constant from the inactive state, k_b_, represent the delay of the inhibition and the reactivation time courses of the head section, respectively.

The concentrations of nicotinamide adenine dinucleotide (NAD) and its reduced form (NADH) depend on the balance of the fluxes which oxidize and reduce them:

$\frac{d\left[NAD\right]}{dt}= \frac{ J_{lac}- J_{tail}+J_{nadh,mit}}{V_{cyt}}$ (23)

NAD and NADH in turn affect J_tail_, Eq. 2, and J_lac_, Eq. 4.

The equations above are used to model metabolism in tumor cells *in vitro* and *in vivo*. Below the model is extended with equations for transport of O_2_, glucose, lactate and pyruvate in tissue.

Transport Equations for Diffusion and Blood Flow in Tissue Containing Two Cell Types

Transport by blood flow and diffusion is modeled with a simple geometry: oxygen and glucose are delivered by flow in a blood vessel which is surrounded by a radially symmetric cylinder of tissue where molecules diffuse and are metabolized. Transport and metabolism of lactate and pyruvate are also taken into account. Transport in tissue from blood vessel to the cells is by diffusion. The tissue contains two separate cell types with distinct metabolic characteristics: each cell type has its own distinct intracellular metabolite concentrations and is represented by a separate set of differential Eqs. 19-23 for its metabolism.

Vascular networks in tumor tissue appear chaotic with asymmetric structures and heterogeneous long and short flow pathways (*45*). This leads to shunting of blood flowing via short pathways, diffusional shunting between adjacent arterial and venous sections of the microcirculation, and complex oxygen profiles with broad distributions of tissue oxygen tension (*46*). Rather than simulate any specific complex tumor vascular network geometry, the aim here is to simulate a simple geometry which reveals basic principles of how fluctuating blood flow causes cycling nutrient and oxygen levels. The model addresses how diffusion gradients influence oxygen and nutrient supply and shows what the metabolic response is of tumor cells with the “Warburg effect” glycolytic phenotype under fluctuating nutrient conditions in tissue.

The oxygen concentration immediately adjacent to a small blood vessel in tumor tissue, O_2,perivascular_, is determined in the model by the balance of oxygen carried by the blood into the microvessel at the arterial side, leaving the microvessel via the venous efflux, and diffusing across the vessel wall into the surrounding tissue:

$\frac{dO_{2,perivascular}}{dt}=(F_{blood}(c_{sat,HbO_{2}}(\frac{c_{a,O_{2}}^{n_{Hill}}}{c_{a,O_{2}}^{n_{Hill}}+c_{50,O_{2},art}^{n_{Hill}}}-\frac{c_{v,O_{2}}^{n_{Hill}}}{c_{v,O_{2}}^{n_{Hill}}+c_{50,O_{2},ven}^{n_{Hill}}}))-J_{diff vessel wall,O_{2}})/V_{perivascular}$ (24)

Variables and parameters in the equations are described in Supplementary Tables 1-3.

For the oxygen dissociation curve of hemoglobin an empirical model is used: the Hill equation c^n^/(c^n^+c_50_^n^) with Hill coefficient n=2.6 and c_50_, the concentration at which hemoglobin’s oxygen binding sites are 50% saturated with O_2_ (*47, 48*). The c_50,O2_ is specified separately for arterial and venous blood mainly because of the pH difference. In Eq. 24 c_v,O2_ is set equal to the perivascular O_2_ concentration, which is a simplifying assumption. In tumor tissue a whole range of oxygen saturations in the microvessels is observed (*49*). O_2,perivascular_ represents the oxygen concentration in the outflow region of an “average” microvessel, which is neither in a short shunt pathway for blood flow, nor in a very long intravascular pathway where hemoglobin oxygen saturation becomes virtually zero. The present simplifying approach is useful to demonstrate the metabolic response of tumor cells with high and low glycolytic capacity to fluctuating supply conditions that occur in tumor tissue, but should not be taken to represent an accurate O_2_ concentration profile for a specific tumor tissue including very heterogeneous perfusion and diffusion pathway lengths.

Diffusion across the microvessel wall is given by Fick’s diffusion law, taking the diffusion coefficient D_O2_, the concentration gradient of oxygen at the vessel wall and the area available for diffusion into account:

$J_{diff vessel wall,O2}=D_{O2}L_{cyl}2\pi(-r_{vessel}\left[\frac{dO_{2}}{dr}\right]_{vessel})$ (25)

where r­­_vessel_ is the radius of the blood vessel and r the radial distance into tissue. Note that the diffusion flux J_O2_ is given per unit volume of intracellular water in the tissue area supplied by the vessel. The cylinder length of the considered volume, L_cyl_, which determines the surface area of the blood vessel available for diffusion, is therefore also normalized per unit intracellular water volume. This maintains the principle of a common reference to be able to compare transport and metabolic flux values.

The rate of change of oxygen concentration in tissue is determined by local oxygen diffusion and by the oxygen consumption, J_O2_, of two different cell types:

$\frac{dO_{2}}{dt}=\frac{-f_{cell type 1}J_{O2,1}-f_{cell type 2}J_{O2,2}+J_{diff, O2}}{V_{ext}+V_{cyt}}$ (26)

The two cell types have distinct V_max_ parameters for the glycolytic head and tail sections represented by equations 1 and 2, respectively, resulting in different glycolytic capacities. The two cell types form a fraction f­_cell type 1_ and f­_cell type 2_ of the tissue. V_ext_ represents the extracellular (interstitial) volume given by the ratio to the intracellular water volume in the same tissue layer.

The tissue is represented by a cylindrical geometry. Diffusion is calculated by a finite difference scheme (*50*) in this cylindrical tissue region, divided into N evenly spaced concentric hollow cylinders (cylindrical shells), one fitting inside the other. A cylindrical shell is defined by inner radius r_inner_ and outer radius r_outer_. Shells with increasing r_inner_ and r­_outer_ and fixed width r_outer_ - r_inner­_ fit inside each other. The blood vessel and perivascular space form the innermost cylinder. The net oxygen diffusion, J_diff,O2_, into a cylindrical shell is determined by Fick’s diffusion law for cylindrical geometry and expressed per unit volume of intracellular water contained in the layer. L_cyl_ is the length of the cylinder per unit volume of intracellular water contained in the same layer.

$J_{diff,O2}=D_{O2}L_{cyl}2\pi(r_{outer}\left[\frac{dO_{2}}{dr}\right]_{outer}-r_{inner}\left[\frac{dO_{2}}{dr}\right]_{inner})$ (27)

The rate of change of diffusing metabolites, M, in the space inside and immediately around the blood vessels is given by:

 (28)

Here M represents glc=glucose, lac=lactate or pyr=pyruvate.

The equation for diffusion of metabolite M across the vessel wall, J_diff vessel wall M­_, is the same as Eq. 25 with M replacing O_2_.

The rate of change of the diffusing metabolites in a tissue layer is given by:

$\frac{dM}{dt}=\frac{(-f_{cell type 1}J_{M,1}-f_{cell type 2}J_{M,2}+J_{diff,M})}{(V_{ext}+V_{cyt})} (M=glc,lac,pyr)$ (29)

Here J_M,1_ and J_M,2_ are the uptake rates of the metabolite in the two cell types (production is represented by negative values). Uptake of glucose equals J_head­_ and uptake of lactate –J_lac_­; pyruvate uptake equals J_lac_ + J_mit,pyr_ - J_tail_. In this way Equations 16-18 for rate of change of the glucose, lactate and pyruvate concentrations in a homogeneous cell suspension are replaced by Eq. 29 for the spatial coordinate dependent concentrations of glucose, lactate and pyruvate.

The net diffusion into the tissue layer is given by:

$J_{diff,M}=D_{M}L_{cyl}2\pi(r_{outer}\left[\frac{dM}{dr}\right]_{outer}-r_{inner}\left[\frac{dM}{dr}\right]_{inner})$ (30)

Simulating tumor tissue with fluctuating blood flow

Blood flow in tumor vessels is often fluctuating or intermittent, e.g. (*51-55*). Fluctuating blood flow was simulated by adding a sinusoidal component with amplitude A_flow_ and period T_period_ to a constant offset O_flow_ :

$F_{blood}=O_{flow}-A_{flow}\sin(2\pi t/T_{period}) with F_{blood}\geq0; t>0$ (31)

F_blood_ = O_flow_ for t<0. Blood flow is only allowed in one direction by imposing zero as lower bound on flow. Consequently, a period of zero blood flow results if the amplitude of the sinusoidal function is larger than the offset. Blood flow levels were taken in the range measured for cancer tissue (*56, 57*).

The conditions in Figures 3-5 and Supplementary Figures 1, 3 and 4 lead to low tissue oxygen concentration due to low and fluctuating blood flow which is characteristic of cycling hypoxia. Chronic hypoxia, in contrast, often results from large diffusion distances in tissue (*52*). The maximal diffusion distance in tissue was set to 40 μm from the blood vessel, as found for the supply region from the blood vessel in the experiments on implanted tumors (*56*) which were used as the example condition for the tissue simulations (see main text). For these relatively small distances in tissue, diffusion limitation has often only a modest influence, and hypoxia is mainly of acute nature due to cycling blood flow. It was assumed that there was no diffusion of oxygen across the 40 μm boundary of the tissue region supplied from the microvessel. This is the non-flux boundary condition often used in tissue simulations of diffusional transport.

For Ehrlich ascites tumor cells being grown in the peritoneal cavity, among others in Warburg’s laboratory, a maximal diffusion distance of 630 μm was used for the simulations (see below). In this case severe hypoxia results from diffusion gradients.

In Figures 3-5 and Supplementary Figures 1 and 3 (see Dataset 4) blood flow was constant for t<0 at 0.264 ml blood per ml intracellular H_2_O per min (11 ml/100g tissue/min) upon which an amplitude of 0.264 ml/ml/min was superimposed to simulate sinusoidally fluctuating blood flow. Cell type 1 constituted 80% of the cells and had the full tumor glycolytic capacity as found in EATC (Figure 2); cell type 2, the remaining 20%, had a glycolytic capacity of 10% of the EATC level, i.e. V_max,head_ and V_max,tail_ had 10% of the value found in EATC. In Supplementary Figure 3 cell type 2, forming 20% of the cell volume, had a glycolytic capacity of 1.5% of the EATC level. Results in Figures 3-5 and Supplementary Figures 1 and 3 are for the tissue cylinder segment at 15-20 μm from the blood vessel.

Simulations of tumor cells in tissue during two minute flow stops, with ATP hydrolysis less sensitive to decrease of energy status, are discussed in detail below.

Reasons for Employing a Coarse-grained Rather than Detailed Model of Glycolysis

Many detailed mathematical models for glycolysis, containing equations for all enzymatic steps, exist. The model by Chance and Hess was already published in 1959 (*2*) and 1960 (*3*). Some more recent examples of models of glycolysis in human and animal cells are the model of Lambeth and Kushmerick (*25*) for muscle, the model of Mulquiney and Kuchel for red blood cells (*19*), the models of Marin-Hernandez et al. (*18*) and Shestov et al. (*22, 23*). Van Eunen et al. (*58*) discussed the difficulties encountered in developing a detailed model of yeast glycolysis. Chandra et al. (*11*) and Van Heerden et al. (*59*) analyzed coarse-grained models of yeast glycolysis. These studies provide additional references, and models of glycolysis are not exhaustively reviewed here.

I had initially implemented two separate detailed models of glycolysis: one set of equations developed specifically for cancer cell glycolysis by Marin-Hernandez et al. (*18*) and another set based on the model of Lambeth and Kushmerick (*25*) extended with the equation for hexokinase by Mulquiney and Kuchel (*19*). Both glycolytic models represent all ten enzymatic steps in the glycolytic chain and both models incorporate both the forward and reverse reaction for all the steps. All the reactions take the Haldane relation and thermodynamic constraints into account. The models were extended with the equation for oxidative phosphorylation also used in the present study.

Despite attempts with both detailed models, not all features of the experimental data on Ehrlich ascites cells could be reproduced well with these detailed models. Especially the measured overshoot of fructose 1,6-bisphosphate (FBP) after adding a high concentration of glucose to EATC was not reproduced well with these detailed models, even when additional regulatory loops were added.

Detailed measurements for all separate glycolytic enzymes in the EATC are not available. The detailed models mentioned above contain 55-70 parameters for the glycolytic chain. The coarse-grained model described in this manuscript depends on fewer parameters. Because parameters specific for EATC were not available for a detailed model and because the number of parameters for a specific EATC model was too large to estimate, the present calculations were based on the reduced model given by Equations 1-23. The system level response is well defined by measurements of glucose uptake (input head part of glycolysis), fructose 1,6-bisphosphate (output head part of glycolysis and input tail part of glycolysis), lactate (output tail part of glycolysis) etc. making parametrization of the coarse-grained model feasible. I therefore employed the course-grained model in this study.

Effect of Reverse Fluxes on the Glycolytic Model

The possible influence of reverse fluxes in the glycolytic chain was investigated in the two detailed mathematical models discussed in the previous section. Both glycolytic models represent the ten enzymatic steps in the glycolytic chain and both models incorporate both the forward and backward reaction for all the steps. All the equations take the Haldane relation into account, and constraints based on thermodynamics are obeyed. Simulations with these models showed that at least one reaction in the head as well as the tail section of the glycolytic chain showed reverse fluxes that were extremely small relative to the forward fluxes. In a simulation with the Lambeth-Kushmerick-Mulquiny model, 1 mM pyruvate was given at t=0 to the glycolytic chain with no glucose present: the reverse flux from the tail part of glycolysis formed merely 0.07 μM fructose 1,6-bisphosphate in two minutes at this high concentration of pyruvate; less than 10^-10^ μM glucose was formed in the same time illustrating the negligible reverse fluxes in the model.

Simulating a response to addition of 0.8 mM glucose (similar to Fig. 2, right hand panel) the maximal forward flux for phosphofructokinase was >300 μM/s, while the maximal backward flux for the same enzyme was less than 0.2 μM/s. For hexokinase the relative effect was even stronger: the maximal forward flux was >300 μM/s, while the maximal backward flux was ~0.007 μM/s. These simulations illustrate the biochemical concept that glycolysis is essentially irreversible, especially the enzymatic reactions by hexokinase, phosphofructokinase and pyruvate kinase, see the textbook of Biochemistry by Berg et al. Sixth Edition, p. 460-463 (*60*). In order to reduce the present model as much as possible, the backward reactions in glycolysis were therefore neglected.

Computational Methods

Programs to calculate computational model results and optimize parameters were written in the R computational language (*61*) and are available under an open source license. The system of ordinary differential equations was integrated using the lsoda algorithm implemented in R package deSolve (*62*). This algorithm switches methods adaptively to solve stiff and non-stiff equation systems (*63*). Diffusion fluxes in tissue or ascites fluid were calculated using a finite difference scheme (*50*). The computer code implements diffusion equations in cylindrical geometries with a variable number of shells and builds the concentric shell structure dynamically. For simulations of tissue *in vivo* eight shells of width 5 μm were used. Parameter optimization procedures based on experimental data and analysis of prediction uncertainty are treated below.

Parameter Optimization

The cost function used for the optimization of model parameters was obtained by adding the squares of the differences between calculated model results and measurements in experiments 1-3 (see below), weighted by estimates of experimental error. Measured data points are given in Figure 2A. The steady state ratio of lactate production to oxygen consumption during one hour after addition of a high concentration of glucose from experiment 3 (see below), measured in Warburg’s laboratory (*4*), provided an additional data point. For the one hour challenge with glucose, the FBP concentration was found experimentally to decline to <50% from 5 min onward and attained stable values after 10 minutes (*64, 65*): squared changes of FBP during each min after 10 min and decrease of FBP levels below 50% were added to the cost function. Model parameters were estimated using a Nelder-Mead (simplex) optimization procedure (*66*) by minimizing the cost function. Results of the parameter optimization are given in Supplementary Table 3. With the estimated parameters, the behavior of the ascites tumor cells in experiments 1-3 was reproduced. Figure 2A shows the fit of the model to the data. In the present study, seventeen parameters were estimated by the Nelder-Mead method, based on 83 data points. This is a relatively large number of parameters. In the next section the question is addressed whether predictions based on model simulations with the estimated parameters are sufficiently well-constrained.

Parameter Sensitivities and Analysis of Prediction Uncertainty

Systems biology models often have many poorly known parameters and “sloppy” parameter sensitivity spectra (*67*). This situation can be meaningfully approached with statistical mechanical methods (*68*). It has been shown that predictive models of the behavior of intact biological systems are usually well-constrained if joint parameter sets for systems biology models are obtained by collective fitting. Markov chain Monte Carlo (MCMC) methods to accomplish this take the (nonlinear) dependencies between the estimated parameters into account (*67*). By using parameters sets from collective fits, this approach can even give good predictions if many or even all individual parameter estimates are rather imprecisely known. Well-constrained predictions were for instance obtained after fitting 59 parameters of a model of a complex signaling network to 68 data points (*69*). Such a strategy to deal with “sloppy” parameter sensitivities was shown to be universally applicable to a large number of systems biology models (*67*), and was previously applied in our laboratory to investigate the creatine kinase energy buffering system in heart (*28*) and skeletal (*70*) muscle.

To assess the uncertainty range of likely parameter distributions around the Nelder-Mead optimized point, different parameter combinations for the present model were generated with an MCMC method, taking the experimental noise in the data into account. The starting point for the Markov chain was the optimal parameter set from the Nelder-Mead method and the same cost function was applied as for the optimization. The collection of parameter sets thus obtained reflects the likelihood of the parameter sets given the experimental data and also reflects correlations and nonlinear dependencies amongst the parameters. For the MCMC approach an adaptive Metropolis-within-Gibbs algorithm was applied (*71*), implemented in R package spBayes (*72*). Forty five Markov chains of 14000-16500 samples each were generated with independent random seeds; each MCMC run took 4-5 days, close to the maximum time allowed on the Lisa cluster computer (see Acknowledgements). Taking the last member of each of the 45 MCMC chains yields parameter sets representing the likelihood of the parameter combinations given the measured data. According to the Heidelberg-Welch statistic the chains tended to converge before the end of the runs and the chains were long relative to the spectrum of autocorrelation times determined with R package “coda” (*73*). The interquartile range for the optimized parameters determined from the 45 replicated MCMC files are given in Supplementary Table 3. The quantiles were calculated according to definition 7 in (*74*), which is the default in R. Some parameters were relatively precisely determined based on the data, while the ranges for other parameters were sometimes very broad. Nevertheless, the model response to the experimental interventions of Figure 2 varied little over the 45 parameter sets (Supplementary Figure 2). This demonstrates that model behavior is well constrained, despite the variation in the precision of parameter determination, as previously shown for other systems biology models (*67*).

The 45 parameter sets obtained by MCMC were then used to repeat the simulations of metabolic responses in tissue in Figure 4, revealing the variation of predictions of system behavior based on the spectrum of estimated model parameter sets (*28*). Supplementary Figure 1 shows the median and the 5 and 95% quantiles of the predictions from the MCMC ensemble. It is clear that the prediction of the metabolic response to blood flow oscillations given in Figure 4 is sufficiently constrained: for each of the MCMC parameter sets the FBP buffering of ATP is much weaker in the cells with the 90% reduced glycolytic capacity compared with cells with the full tumor glycolytic capacity.

Calibrating the computational model with experimental data

A data set was assembled consisting of representative experiments to estimate the model parameters for the predictive model of metabolic responses of Ehrlich ascites tumor cells. These data sets from the scientific literature are exemplary, but they are representative of results measured in many laboratories (*1, 2, 4-10, 32, 33, 35, 64, 75-78*). All selected experiments were done at 37 ^o^C on Ehrlich ascites cells which had been grown in mice. During the experiments aerated tumor cell suspensions were diluted in buffer solution. Cells and suspension had been depleted of glucose for some time and were respiring on endogenous substrates such as lactate, which was abundantly present. At t=0 glucose was added to the suspension. Experimental methods were described in detail in the original publications. The following experiments were included in the calibration data set:

Experiment 1. In this experiment 3.5 µmol glucose was added per ml of cell volume to an Ehrlich ascites cell suspension in which glucose was depleted and which was respiring on endogenous substrates, mainly lactate, present in the suspension (*7*). The glucose addition resulted in a start concentration of 92 µM glucose at t=0. Cell volume fraction was 2.2%. Amount of glucose taken up and FBP and ATP concentrations were measured at 0, 10, 20, 30, 45, 60, 90, 120 and 180 seconds after glucose addition and included in the data set. (The lactate measurements reported for this experiment were not included because they were later reported to be imprecise by the same research group (*33*).)

Experiment 2. Here 26.6 µmol glucose was added per ml of cell volume, resulting in a start concentration of 776 µM glucose at a cell volume fraction of 2.9%. Before glucose addition, cells were metabolizing endogenous substrates (*33*) including lactate. Amounts of glucose taken up, FBP and ATP concentrations and amount of lactate produced were measured at 0, 5, 10, 15, 20, 30, 45, 60, 90, 120, 180, 240 and 300 seconds after glucose addition and were included in the data set. Oxygen uptake measurements with an oxygen electrode were also included in the data set. ATP production before glucose addition was ~199 µM/s, based on respiration. The calculated mass balance of the experimental data (see Figure 2) on phosphate metabolites (ATP and FBP), lactate production (1 lactate molecule set equivalent to 1 ATP synthesized) and respiration showed that 15 sec after glucose addition ATP hydrolysis had decreased to around a third of the initial value, and recovered to above 100 µM/s after 4-5 min. Analysis of the data showed that ATP hydrolysis correlated with the sum of ATP and ADP rather than with ATP or the ATP/ADP ratio. ATP hydrolysis was approximately linearly related to the sum of the ATP and ADP concentrations. The calculated balance of glucose taken up and accumulated amounts of FBP, lactate, pyruvate, taking into account pyruvate consumption by the mitochondria, showed that by 300 s one third of the carbon atoms in the sequestered glucose was taken up in intracellular metabolite stores. Intracellular storage of carbon, see Eq. 9, originating from the glucose taken up, is therefore required to obtain a carbon balance in the model agreeing with the experimental data.

Experiment 3. Experiments in Warburg’s laboratory reported by Tiedemann (*4*). The uptake of oxygen and production of lactate during one hour after addition of 11.1 mmol glucose per liter to a 0.43% suspension of Ehrlich ascites tumor cells was measured. In this experiment the cells were suspended in salt-containing buffer without amino acids. Under these conditions protein synthesis in EATC decreases within the first 5-10 min and glucose from the medium is depleted at a reduced rate (*79*). In the model calculations storage of glucose in intracellular metabolite pools was therefore reduced if it exceeded the level obtained after this first period.

Testing the computational model with additional experimental data

Model predictions were compared with five experimental data sets that had not been used in the parameter estimation:

Experiment 4. Experiments where different amounts of glucose were given. Model predictions were compared with the measurements to test the dependence of model results on glucose concentration. EATC respiring on endogenous substrates were given ten different amounts of glucose at t=0 (across five separate experiments) resulting in initial glucose concentrations between 29 and 800 μM. The maximal FBP accumulation was measured (*7*). In the simulations the maximal FBP concentration was predicted for each amount of glucose and compared with the experimental data.

Experiment 5. Fast metabolic responses in the first ten seconds were examined. Start concentrations of 77 and 154 µM glucose were given at t=0 to EATC which respired on endogenous substrates before that time. The FBP accumulated and the lactate produced in 5 and 10 sec were measured (Figure 6 in reference (*33*)). This agrees with the simulations (see Dataset 1, worksheets `Exp 5` and `Accumulation in 5 and 10 sec exp 5`).

Additional support comes from data in Saha and Coe (*43*) (see Table I in that reference) on measured glucose uptake and FBP accumulation after 10 s for a range of added glucose concentrations (0.2-10 mM). Both computer simulations and experimental results showed saturation of the FBP accumulation above 400 µM glucose.

Experiment 6. Anaerobic metabolism measured by Tiedemann in Warburg’s laboratory. As in experiment 3 an initial concentration of 11.1 mM glucose was added to a 0.43% cell suspension at the start of the measurement. Hydrogen cyanide (HCN) had also been added, which inhibits mitochondrial oxygen consumption (*4*). In the simulation oxygen consumption was also inhibited. Oxygen uptake and lactate production with and without HCN were compared between experiment and simulation. Simulation with and without mitochondrial oxygen consumption active are given in Datasets 1 and 2, respectively. Note that this experiment was reported in the same paper as Experiment 3.

Experiment 7. Effect of pyruvate on metabolite accumulation after glucose addition. ATP levels decline appreciably in simulations and measurements when glucose is added, but when 5 mM pyruvate is added at the same time as glucose, the decline of ATP is reduced to merely 0.1% in the simulation, as opposed to 30% when the concentration of pyruvate is low. This is similar as the pattern seen in experiments on ascites tumor cells (*37*). Simulation results are given in Dataset 3.

Experiment 8. Effect of addition of glucose on respiration. O_2_ consumption of EATC during one hour was measured in Warburg’s laboratory, comparing 0 and 11 mM glucose in the medium. The reduction of respiration, known as the Crabtree effect, was assessed (*4*). Note that this was reported in the same paper from Warburg’s laboratory as Experiments 3 and 6.

The relatively small computational model adequately represents these eight experimental data sets selected from the literature. These data sets are representative of results repeatedly measured in many separate laboratories, e.g. (*1, 2, 4-6, 8-10, 16, 17, 20, 31-35, 37, 38, 43, 65, 75-78, 80, 81*). Note for instance how Chance (*6*) reports a similar very high glucose uptake during the first minute after glucose addition followed by a strong decrease as simulated by the present model (Figure 2, Dataset 1). Initial accumulation of FBP is followed by a secondary decrease, and an initial dip in ATP concentration is followed by an increase to a level lower than the initial one (*6*). An initial stimulation of respiration is measured after glucose addition, followed by a decrease below the level measured before glucose (*5*). Despite its simplicity, the present model appears to adequately integrate a broad set of biochemical knowledge and experimental data on the metabolic behavior of ascites tumor cells. The model therefore appears suitable to simulate the metabolic response of ascites tumor cells and other tumor cells in tissue.

Simulation of tumor cells with flow stops and low sensitivity of ATP hydrolysis to energy status

In the simulations of Figures 3-5 and Supplementary Figures 1 and 3, the sensitive mechanism to adapt ATP hydrolysis to energy status plays an important role. To demonstrate how the powerful glycolytic machinery may protect the cell on its own if this ATP hydrolysis reducing mechanism is less effective, a second hypothetical situation in tissue was investigated. The simulations in Supplementary Figure 4 and Dataset 5 focus therefore on the effect of dynamic ATP buffering by the tail section of glycolysis. To accomplish this, adenine nucleotide breakdown was set to be not active in the simulation. The strong reduction of ATP hydrolysis with AdN = ATP+ADP, found in the in vitro experiments (Figure 2A, Eq. 5), was replaced by weak reduction of ATP hydrolysis with decreasing ATP concentration. AdN is therefore replaced by ATP in Eqs. 5 and 6. For the simulation in Supplementary Figure 4 and Dataset 5 k_hyd,ATP_ was set to 0.00833 s^-1^. This value is similar as measured for protein degradation, see Figure 7 in Gronostajski et al. (*39*). The ATP_cutoff_ value was set to 1600 μM, C_power_ = 23.51, γ = 0.166 and k_hyd,AdN,low_ = 0 s^-1^. Eqs. 5 and 6 now define a curve whose slope decreases with ATP, remaining constant above the ATP_cutoff_ value .

The metabolic rate was kept relatively low, V_max,hyd_ = 100 μM/s, and there was no storage of glucose-derived metabolites, corresponding with no growth of the tumor cells. For t<0 blood flow was constant at 0.132 ml blood per ml intracellular H_2_O per min (corresponding to 5.4 ml/100 g wet weight/min). Blood flow started to oscillate sinusoidally at t=0 with period 500 sec and an amplitude of 0.21 ml blood per ml intracellular H_2_O per min (8.7 ml/100 g wet weight/min). Blood flow was constrained to be >0 (top panel, Supplementary Figure 4), which result in flow stops of more than 2 min. The first cell type, comprising 95% of the cells, had the full tumor glycolytic capacity as found in EATC (Figure 2); cell type 2, the remaining 5%, had a glycolytic capacity of 1.5-50% of the EATC value as indicated above the rows in Supplementary Figure 4, which each represent separate simulations. The blood flow level and absence of growth are similar to conditions in old implanted tumors (*56*). The effect of the ATP buffering by FBP and other PGI is still active and its effect can therefore be assessed in isolation from a mechanism of reduction of ATP hydrolysis which is very sensitive to adenine nucleotides.

Although the average blood flow for t>0 was slightly higher than for t<0, ATP levels declined at low and stopped flow, but only in the cells with decreased glycolytic capacity. Results in Supplementary Figure 4 were for the tissue layer at 35-40 μm from the blood vessel. As a result of the small sensitivity of ATP hydrolysis to energy status there was a large decline of ATP when glycolytic capacity was only modestly lower than in tumor cells (Supplementary Figure 4). The simulations show that FBP buffering supported by the full tumor glycolytic capacity will protect the cell also in this case where adaptation of ATP hydrolysis to decreased adenine nucleotide levels is not effective. In contrast, modest reductions in glycolytic capacity lead to decreased ATP concentrations and hydrolysis during the low flow period. When ATP synthesis in the tail section from the FBP buffer is uncoupled such that effectively no ATP is made, ATP levels fall dramatically even when glycolytic capacity is high, but only when blood flow fluctuates (Supplementary Figure 4, bottom row).

Simulation of glucose metabolism of Ehrlich tumor cells in ascites fluid

Warburg and Hiepler (*81*) noted that glucose and oxygen contents were very low in the ascites fluid in which they grew Ehrlich ascites tumor cells (EATC) in the peritoneal cavity of mice. They predicted from *in vitro* measurements that these cells should consume 44 mg glucose per hour if exposed anaerobically to high glucose concentrations *in vivo* in the ~5 ml ascites fluid found in these mice. They surmised that this amount of glucose could not be provided by the host. Indeed, Kemp and Mendel (*82*) injected 5 ml ascites fluid, without EATC, intraperitoneally in mice and measured that at most 14 mg of glucose could diffuse into this fluid per hour. They also measured 5-7 mg/dl (280-390 μM) glucose concentration in ascites fluid containing growing EATC, which is sufficient for glycolytic rates very close to the maximal values found *in vitro*. This constitutes a paradox: the glucose concentration in samples of ascites fluid appears to be sufficient for high rates of glucose consumption by the tumor cells, but the transport capacity for glucose from blood into the ascites fluid is not high enough to sustain these high metabolic rates. To resolve this paradox, Kemp and Mendel suggested that in the presence of proliferating ascites cells *in vivo*, the diffusion gradient for glucose would be kept higher because the cells consume glucose at a high rate. They hypothesized that this would allow sufficient glucose diffusion when EATC metabolize glucose at near-maximal rates. Kemp and Mendel proposed further that the liver of the murine host resynthesizes glucose from the lactate generated by the EATC to sustain the high glucose metabolism in the ascites tumor cells. It is of some historical interest that Warburg endorsed their explanation in a note added retrospectively to the Hiepler and Warburg (*81*) paper of 1952 when this paper was reprinted in a collection volume in 1961(*83*).

Burgess and Sylvén (*84*) also measured a low glucose concentration, around 300 µM in ascites fluid where EATC were growing. They found glucose concentrations of ~145 mg/dl (~8 mM) in normal peritoneal fluid not containing EATC. Note that mixing of glucose-depleted ascites fluid with a small amount of peritoneal fluid from parts of the peritoneal cavity which do not contain glycolytically active EATC might therefore cause average glucose concentrations in sampled ascites fluid above 200 µM.

The hypothesis is now investigated here that diffusion gradients for glucose in the ascites fluid containing tumor cells in the abdomen of mice are large. The peritoneal surface in mice is estimated to be 79 cm^2^ (*85*). For a peritoneal fluid volume of 5 ml (*82*) this means that the peritoneal surface can be thought of as being covered by a fluid layer of ~630 µm on average. Although this is a simplified approximation to a much more heterogeneous situation, diffusion distances are probably large for a substantial part of the ascites fluid. Consequently, glucose concentrations may become limiting for glucose uptake of the cells in a major fraction of the ascites fluid. This is illustrated here by simulation with the model for metabolism (Eqs. 1-23) including diffusion from blood vessels into ascites fluid containing the tumor cells (Eqs. 24-30).

First the experiment of Kemp and Mendel (*82*) to measure the glucose transport capacity into ascites fluid without cells is simulated: 5 ml of cell-free ascitic fluid, containing 3 mg/dl (~167 µM) glucose, was injected into the peritoneal cavity of mice. Due to diffusion, the average ascites glucose concentration increased to 17 mg/dl (944 µM) in 3 min. A simulation of a 630 µm layer of ascites fluid on the peritoneal membrane showed a similar increase in average glucose concentration (Dataset 6). With tumor cells (25% of the volume) in the ascites fluid and the same glucose supply conditions, glucose consumption by the simulated EATC is ~49 µM/s at diffusion distances up to 100 µm from the peritoneal blood vessels. This increases to ~78 µM/s at ~170 µm because the O_2_ concentration has fallen close to zero and anaerobic glycolysis supplies the ATP (Dataset 7). Beyond 300 µm diffusion distance from a blood vessel, glucose and O_2_ concentrations in the ascites fluid are both so low that cellular glucose uptake and ATP production become zero; this applies to the larger part of the simulated ascites fluid volume. At the same time the average glucose concentration is ~119 µM, which is more than twofold the K_m_ value for glucose. The average cellular glucose uptake, ~13 µM/s, in the ascites tumor cells in the simulation of the situation *in vivo* is much lower than for Warburg’s *in vitro* measurements (*81*) which yielded ~55-68 µM/s under anaerobic conditions and ~35 µM/s under aerobic conditions (*4*). The average uptake of 13 µM/s corresponds with ~9 mg per hour per 5 ml which is within the diffusion capacity reported by Kemp and Mendel, see above (*82*). Cellular glucose uptake in the ascites fluid is probably very heterogeneous because of the large diffusion gradients. Perfusion heterogeneity in the peritoneum may cause additional variation. According to the simulation results, a substantial fraction of cells at diffusion distances 130-300 µm from blood vessels are exposed to a combination of O­_2_ < 0.1 µM with glucose still present, which results in anaerobic glycolysis.

The tumor cells in the ascites fluid are expected to change position relative to the blood vessels in the peritoneum because of intestinal peristalsis, muscle contraction and body movement (*86*). Injected dye spreads through the peritoneal cavity of mice in 2-4 min (*87*). The time required to establish a new diffusion gradient after movements of cells relative to the peritoneal blood vessels was simulated in two ways. In the first simulation approach, the concentration of metabolites was initially set constant at t=0 throughout the fluid volume at the average value and the relaxation of concentration towards the steady-state diffusion gradient was simulated for t>0. According to these simulations O_2_ concentrations typically adjust upward in 1-2 s with initial rate up to 50 μM/s, while downward changes take longer with initial rate <10 μM/s. Increases of glucose concentration take ~20 s at initial rates up to 500 μM/s while downward changes take >1 min at initial rate ≤15 μM/s. Downward changes in concentration in deeper layers further removed from blood vessels affect the upper layers with delay and diminished amplitude, causing two phases in the response.

The second simulation approach to investigate the speed of adaptation of concentrations to displacement of fluid started from the steady-state diffusion gradient (found in Dataset 7) and fluid was moved at t=0 from 200-400 μm from the blood vessel to 0-200 μm and at the same time fluid from 0-200 μm was moved to 200-400 μm. Oxygen concentration in ascites fluid moving close to a peritoneal blood vessel increases within 2 s from <0.1 μM to above 90 μM and glucose concentration from 150 to 4850 μM. A downward change in oxygen concentration in fluid moving away from the vascular oxygen source is virtually complete in 2 s, but the decline of a high glucose concentration takes much longer. In this second type of simulation the concentration changes were secondarily affected by changes in metabolism throughout the ascites fluid. Cells moving quickly closer to a blood vessel from a distance >200 μm, largely depleted of FBP, show a metabolic response which is in some respects similar as in Figure 2, with strong initial glucose uptake, FBP accumulation and transient overshoot of oxygen consumption.

In short, simulations suggest that a large fraction of the ascites fluid contains very low O_2_ and glucose concentrations. The movement of fluid and cells may therefore cause large fluctuations in nutrient supply. In this way the ascites fluid in the intraperitoneal cavity forms an environment for ascites tumor cells where a nutrient and energy buffering system to survive periods of nutritional shortage may provide a large competitive and selective advantage to the cells.

**References**

1. E. L. Coe, W. V. V. Greenhouse, Possible regulatory interactions between compartmentalized glycolytic systems during initiation of glycolysis in ascites tumor cells. *Biochim Biophys Acta* **329**, 171-182 (1973).

2. B. Chance, B. Hess, Spectroscopic evidence of metabolic control. *Science* **129**, 700-708 (1959).

3. B. Chance, D. Garfinkel, J. Higgins, B. Hess, A solution for the equations representing interaction between glycolysis and respiration in ascites tumor cells. *Journal of Biological Chemistry* **235**, 2426-2439 (1960).

4. H. Tiedemann, Über den Stoffwechsel des Ascites-Tumors der Maus. *Zeitschrift fiir die gesamte experimentelle Medizin* **119**, 272-279 (1952).

5. B. Chance, B. Hess, Metabolic control mechanisms III. Kinetics of oxygen utilization in ascites tumor cells. *Journal of Biological Chemistry* **234**, 2416-2420 (1959).

6. B. Hess, B. Chance, Metabolic control mechanisms. VI. Chemical events after glucose addition to ascites tumor cells. *Journal of Biological Chemistry* **236**, 239-246 (1961).

7. E. L. Coe, K. H. Ibsen, M. Dixon, R. W. McKee, Glycolysis of small amounts of glucose by Ehrlich ascites carcinoma cells. *Cancer Research* **26**, 276-281 (1966).

8. E. L. Coe, Correlation of glycolytic and respiratory events after addition of a small amount of glucose to Ehrlich ascites carcinoma. *Cancer Research* **26**, 269-275 (1966).

9. K. K. Lonberg, A direct study of intracellular glycolysis in Ehrlich's ascites tumor. *Biochimica et Biophysica Acta* **35**, 464-472 (1959).

10. G. Wilhelm, J. Schulz, E. Hoffmann, pH-Abhängigkeit von Glykolyse und Atmung in Ehrlich-Ascitestumorzellen. *Acta Biologica et Medica Germanica* **29**, 1-16 (1972).

11. F. A. Chandra, G. Buzi, J. C. Doyle, Glycolytic oscillations and limits on robust efficiency. *Science* **333**, 187-192 (2011).

12. M. W. Dewhirst, Y. Cao, B. Moeller, Cycling hypoxia and free radicals regulate angiogenesis and radiotherapy response. *Nat Rev Cancer* **8**, 425-437 (2008).

13. L. I. Cardenas-Navia, D. Mace, R. A. Richardson, D. F. Wilson, S. Shan, M. W. Dewhirst, The pervasive presence of fluctuating oxygenation in tumors. *Cancer Res* **68**, 5812-5819 (2008).

14. H. Kimura, R. D. Braun, E. T. Ong, R. Hsu, T. W. Secomb, D. Papahadjopoulos, K. Hong, M. W. Dewhirst, Fluctuations in red cell flux in tumor microvessels can lead to transient hypoxia and reoxygenation in tumor parenchyma. *Cancer Research* **56**, 5522-5528 (1996).

15. A. Marin Hernandez, S. Rodriguez-Enriquez, P. A. Vital-Gonzalez, F. L. Flores-Rodriguez, M. Macias-Silva, M. Sosa-Garrocho, R. Moreno-Sanchez, Determining and understanding the control of glycolysis in fast-growth tumor cells. Flux control by an over-expressed but strongly product-inhibited hexokinase. *FEBS J* **273**, 1975-1988 (2006).

16. K. A. Gumaa, P. McLean, Sequential control of hexokinase in ascites cells. *Biochemical and Biophysical Research Communications* **35**, 824-831 (1969).

17. D. P. Kosow, I. A. Rose, Origin of the delayed feedback control of glucose utilization in ascites tumor cells. *Biochem Biophys Res Commun* **48**, 376-383 (1972).

18. A. Marin-Hernandez, J. C. Gallardo-Perez, S. Rodriguez-Enriquez, R. Encalada, R. Moreno-Sanchez, E. Saavedra, Modeling cancer glycolysis. *Biochim Biophys Acta* **1807**, 755-767 (2011).

19. P. J. Mulquiney, P. W. Kuchel, Model of 2,3-bisphosphoglycerate metabolism in the human erythrocyte based on detailed enzyme kinetic equations: equations and parameter refinement. *Biochem J* **342**, 581-596 (1999).

20. K. A. Gumaa, P. McLean, The pentose phosphate pathway of glucose metabolism. Enzyme profiles and transient and steady-state content of intermediates of alternative pathways of glucose metabolism in Krebs ascites cells. *Biochem J* **115**, 1009-1029 (1969).

21. L. Bosca, J. J. Aragon, A. Sols, Specific activation by fructose-2,6-bisphosphate and inhibition by P-enolpyruvate of ascites tumor phosphofructokinase. *Biochemical and Biophysical Research Communications* **106**, 486-491 (1982).

22. A. A. Shestov, X. Liu, Z. Ser, A. A. Cluntun, Y. P. Hung, L. Huang, D. Kim, A. Le, G. Yellen, J. G. Albeck, J. W. Locasale, Quantitative determinants of aerobic glycolysis identify flux through the enzyme GAPDH as a limiting step. *Elife* **3**, (2014).

23. M. V. Liberti, Z. Dai, S. E. Wardell, J. A. Baccile, X. Liu, X. Gao, R. Baldi, M. Mehrmohamadi, M. O. Johnson, N. S. Madhukar, A. A. Shestov, I. I. C. Chio, O. Elemento, J. C. Rathmell, F. C. Schroeder, D. P. McDonnell, J. W. Locasale, A Predictive Model for Selective Targeting of the Warburg Effect through GAPDH Inhibition with a Natural Product. *Cell Metab* **26**, 648-659 e648 (2017).

24. S. Bagui, M. Ray, S. Ray, Glyceraldehyde-3-phosphate dehydrogenase from Ehrlich ascites carcinoma cells. Its possible role in the high glycolysis of malignant cells. *Eur J Biochem* **262**, 386-395 (1999).

25. M. J. Lambeth, M. J. Kushmerick, A computational model for glycogenolysis in skeletal muscle. *Annals of Biomedical Engineering* **30**, 808-827 (2002).

26. M. S. Jurica, A. Mesecar, P. R. Heath, W. Shi, T. Nowak, B. L. Stoddard, The allosteric regulation of pyruvate kinase by fructose-1,6-bisphosphate. *Structure* **6**, 195-210 (1998).

27. H. R. Christofk, M. G. Vander Heiden, N. Wu, J. M. Asara, L. C. Cantley, Pyruvate kinase M2 is a phosphotyrosine-binding protein. *Nature* **452**, 181-186 (2008).

28. H. Hettling, J. H. van Beek, Analyzing the functional properties of the creatine kinase system with multiscale 'sloppy' modeling. *PLoS Comput Biol* **7**, e1002130 (2011).

29. G. Froese, The respiration of ascites tumor cells at low oxygen concentration. *Biochimica en Biophysica Acta* **57**, 509-519 (1962).

30. C. D. Stoner, H. D. Sirak, Steady-state kinetics of the overall oxidative phosphorylation reaction in heart mitochondria. *Journal of Bioenergetics and Biomembranes* **11**, 113-146 (1979).

31. K. H. Ibsen, E. L. Coe, R. W. McKee, Some factors influencing respiration and glycolysis in Ehrlich ascites tumor cells. *Cancer Research* **20**, 1399-1407 (1960).

32. R. Wu, E. Racker, Regulatory Mechanisms in Carbohydrate Metabolism: IV Pasteur effect and Crabtree effect in ascites tumor cells. *Journal of Biological Chemistry* **234**, 1036-1041 (1959).

33. I.-L. Lee, R. C. Strunk, E. L. Coe, Coordination among rate-limiting steps of glycolysis and respiration in intact ascites tumor cells. *Journal of Biological Chemistry* **242**, 2021-2028 (1967).

34. E. L. Coe, I.-Y. Lee, Phosphorylation of 2-D-deoxyglucose and associated inorganic phosphate uptake in ascites tumor cells. *Biochemistry* **8**, 685-693 (1969).

35. R. Wu, E. Racker, Regulatory mechanisms in glycolysis of ascites tumor cells: III. Limiting factors in glycolysis of ascites tumor cells. *Journal of Biological Chemistry* **234**, 1029-1035 (1959).

36. M. Müller, W. Siems, F. Buttgereit, R. Dumdey, S. M. Rapoport, Quantification of ATP-producing and consuming processes of Ehrlich ascites tumor cells. *Eur J Biochem* **161**, 701-705 (1986).

37. G. Glaser, H. Giloh, J. Kasir, M. Gross, J. Mager, On the mechanism of the glucose-induced ATP catabolism in ascites tumour cells and its reversal by pyruvate. *Biochem J* **192**, 793-800 (1980).

38. K. Overgaard-Hansen, Metabolic regulation of the adenine nucleotide pool. I. Studies on the transient exhaustion of the adenine nucleotides by glucose in Ehrlich ascites tumor cells. *Biochim Biophys Acta* **104**, 330-347 (1965).

39. R. M. Gronostajski, A. B. Pardee, A. L. Goldberg, The ATP dependence of the degradation of short- and long-lived proteins in growing fibroblasts. *The Journal of Biological Chemistry* **260**, 3344-3349 (1985).

40. S. P. Soltoff, L. J. Mandel, Active ion transport in the renal proximal tubule. III. The ATP dependence of the Na pump. *Journal of General Physiology* **84**, 643-662 (1984).

41. K. Kroll, D. Kinzie, L. A. Gustafson, Open system kinetics of myocardial phosphorenergetics during coronary underperfusion. *American Journal of Physiology Heart Circulatory Physiology* **272**, H2563-H2576 (1997).

42. P. Borst, The aerobic oxidation of reduced diphosphopyridine nucleotide formed by glycolysis in Ehrlich ascites-tumour cells. *Biochimica en Biophysica Acta* **57**, 270-282 (1962).

43. J. Saha, E. L. Coe, Evidence indicating the existence of two modes of glucose uptake in Ehrlich ascites tumor cells. *Biochem Biophys Res Commun* **26**, 441-446 (1967).

44. T. L. Spencer, A. L. Lehninger, L-Lactate transport in Ehrlich ascites-tumour cells. *Biochem J* **154**, 405-414 (1976).

45. A. R. Pries, M. Hopfner, F. le Noble, M. W. Dewhirst, T. W. Secomb, The shunt problem: control of functional shunting in normal and tumour vasculature. *Nat Rev Cancer* **10**, 587-593 (2010).

46. T. W. Secomb, R. Hsu, E. Y. H. Park, M. W. Dewhirst, Green's function methods for analysis of oxygen delivery to tissue by microvascular networks. *Ann Biomed Eng* **32**, 1519-1529 (2004).

47. O. Siggaard-Andersen, *The Acid-base Status of the Blood*. (Munksgaard, Copenhagen, ed. Fourth Edition, 1976).

48. C.-F. Cartheuser, Standard and pH-affected hemoglobin-O2 binding curves of Sprague-Dawley rats under normal and shifted P50 conditions. *Comp Biochem Physiol* **106A**, 775-782 (1993).

49. P. Vaupel, R. Manz, W. Müller-Klieser, W. A. Grunewald, Intracapillary HbO2 saturation in malignant tumors during normoxia and hyperoxia. *Microvascular Research* **17**, 181-191 (1979).

50. J. Crank, *The Mathematics of Diffusion*. (Clarendon Press, Oxford, ed. Second Edition., 1975), pp. 414.

51. R. C. Mesquita, S. W. Han, J. Miller, S. S. Schenkel, A. Pole, T. V. Esipova, S. A. Vinogradov, M. E. Putt, A. G. Yodh, T. M. Busch, Tumor blood flow differs between mouse strains: consequences for vasoresponse to photodynamic therapy. *PLoS One* **7**, e37322 (2012).

52. M. W. Dewhirst, H. Kimura, S. W. E. Rehmus, R. D. Braun, D. Papahadjopoulos, K. Hong, T. W. Secomb, Microvascular studies on the origins of perfusion-limited hypoxia. *British Journal of Cancer* **74**, S247-S251 (1996).

53. D. T. Fisher, J. B. Muhitch, M. Kim, K. C. Doyen, P. N. Bogner, S. S. Evans, J. J. Skitzki, Intraoperative intravital microscopy permits the study of human tumour vessels. *Nat Commun* **7**, 10684 (2016).

54. M. W. Dewhirst, Relationships between cycling hypoxia, HIF-1, angiogenesis and oxidative stress. *Radiat Res* **172**, 653-665 (2009).

55. K. H. Pigott, S. A. Hill, D. J. Chaplin, M. I. Saunders, Microregional fluctuations in perfusion within human tumours detected using laser Doppler flowmetry. *Radiation Therapy and Oncology* **40**, 45-50 (1996).

56. P. Vaupel, H. Günther, J. Grote, Atemgaswechsel und Glucosestoffwechsel von tumoren (DS-Carcinosarkom) in vivo. I. Experimentelle Untersuchungen der versorgungsbestimmenden parameter. *Z ges exp Med* **156**, 283-294 (1971).

57. P. Vaupel, Hypoxia in neoplastic tissue. *Microvascular Research* **13**, 399-408 (1977).

58. K. van Eunen, J. A. Kiewiet, H. V. Westerhoff, B. M. Bakker, Testing biochemistry revisited: how in vivo metabolism can be understood from in vitro enzyme kinetics. *PLoS Comput Biol* **8**, e1002483 (2012).

59. J. H. van Heerden, M. T. Wortel, F. J. Bruggeman, J. J. Heijnen, Y. J. Bollen, R. Planque, J. Hulshof, T. G. O'Toole, S. A. Wahl, B. Teusink, Lost in transition: start-up of glycolysis yields subpopulations of nongrowing cells. *Science* **343**, 1245114 (2014).

60. J. M. Berg, J. L. Tymoczko, L. Stryer, *Biochemistry*. (W.H. Freeman and Company, ed. Sixth, 2007).

61. http://www.r-project.org.

62. K. Soetaert, T. Petzoldt, R. Setzer, Solving differential equations in R: package deSolve. *Journal of Statistical Software* **33**, 1-25 (2010).

63. L. Petzold, Automatic selection of methods for solving stiff and nonstiff systems of ordinary differential equations. *SIAM Journal on Scientic and Statistical Computing* **4**, 136-148 (1983).

64. G. Wilhelm, J. Schulz, E. Hofmann, pH-dependence of aerobic glycolysis in Ehrlich ascites tumour cells. *FEBS Letters* **17**, 158-162 (1971).

65. K. H. Ibsen, E. L. Coe, R. W. McKee, Interrelationships of metabolic pathways in the Ehrlich ascites carcinoma cells. I. Glycolysis and respiration (Crabtree effect). *Biochimica en Biophysica Acta* **30**, 384-400 (1958).

66. J. Nelder, R. Mead, A simplex method for function minimization. *The Computer Journal* **7**, 308-313 (1965).

67. R. N. Gutenkunst, J. J. Waterfall, F. P. Casey, K. S. Brown, C. R. Myers, J. P. Sethna, Universally sloppy parameter sensitivities in systems biology models. *PLoS Comput Biol* **3**, 1871-1878 (2007).

68. K. S. Brown, J. P. Sethna, Statistical mechanical approaches to models with many poorly known parameters. *Physical Review E* **68**, (2003).

69. K. S. Brown, C. C. Hill, G. A. Calero, C. R. Myers, K. H. Lee, J. P. Sethna, R. A. Cerione, The statistical mechanics of complex signaling networks: nerve growth factor signaling. *Phys Biol* **1**, 184-195 (2004).

70. J. H. van Beek, F. Supandi, A. K. Gavai, A. A. de Graaf, T. W. Binsl, H. Hettling, Simulating the physiology of athletes during endurance sports events: modelling human energy conversion and metabolism. *Philos Trans A Math Phys Eng Sci* **369**, 4295-4315 (2011).

71. J. S. Rosenthal, AMCMC: An R interface for adaptive MCMC. *Computational Statistics and Data Analysis* **51**, 5467-5470 (2007).

72. A. O. Finley, S. Banerjee, A. E. Gelfand, spBayes for large univariate and multivariate point-referenced spatio-temporal models. *Journal of Statistical Software* **63**, 1-28 (2015).

73. M. Plummer, N. Best, K. Cowles, K. Vines, CODA: Convergence Diagnosis and Output Analysis for MCMC *R News* **6**, (2006).

74. R. J. Hyndman, Y. Fan, Sample quantiles in Statistical Packages. *The American Statistician* **50**, 361-365 (1996).

75. B. Hess, B. Chance, Phosphorylation efficiency of the intact cell. I. Glucose-oxygen titrations in ascites tumor cells. *234*, 3031-3035 (1959).

76. E. L. Coe, Correlations between adenine nucleotide levels and the velocities of rate-determining steps in the glycolysis and respiration of intact Ehrlich ascites carcinoma cells. *Biochimica et Biophysica Acta* **118**, 495-511 (1966).

77. E. L. Coe, Transient isolation of the hexokinase reaction from the glycolytic sequence on initiation of glycolysis in ascites tumor cells. *Biochem Biophys Res Commun* **38**, 1105-1112 (1970).

78. I.-Y. Lee, E. L. Coe, Theoretical phosphorylation rates after addition of a small amount of glucose to intact ascites tumor cells. *Biochim Biophys Acta* **131**, 441-452 (1967).

79. T. R. Live, E. Kaminskas, Changes in adenylate energy charge in Ehrlich ascites tumor cells deprived of serum, glucose, or amino acids. *Journal of Biological Chemistry* **250**, 1786-1789 (1975).

80. O. Warburg, On the origin of cancer cells. *Science* **123**, 309-314 (1956).

81. O. Warburg, Z. Hiepler, Versuche mit Ascites-Tumorzellen. *Z Naturforschung* **7b**, 193-194 (1952).

82. A. Kemp, B. Mendel, How does the Ehrlich ascites tumour obtain its energy for growth? *Nature* **180**, 131-132 (1957).

83. O. Warburg, *New Methods of Cell Physiology*. (Interscience Publishers New York - a division of John Wiley, 1962), pp. 644.

84. E. A. Burgess, B. Sylvén, Changes in glucose and lactate content of ascites fluid and blood plasma during growth and decay of the ELD ascites tumour. *Br J Cancer* **16**, 298-305 (1962).

85. A. Rippe, C. Rippe, K. Sward, B. Rippe, Disproportionally low clearance of macromolecules from the plasma to the peritoneal cavity in a mouse model of peritoneal dialysis. *Nephrol Dial Transplant* **22**, 88-95 (2007).

86. G. Klein, Some recent studies on the production and growth characteristics of ascites tumors. *Zeitschrift für Krebsforschung* **61**, 99-119 (1956).

87. L. Révész, G. Klein, Quantitative studies on the multiplication of neoplastic cells in vivo. II. Growth curves of three ascites lymphomas. *J Nat Cancer Inst* **15**, 253-273 (1954).
