# Supplementary material for: The dynamic side of the Warburg effect: glycolytic intermediate storage as buffer for fluctuating glucose and O 2 supply in tumor cells
Source: F1000Res. 2018 Dec 28;7:1177. Originally published 2018 Aug 2. [Version 2] doi: 10.12688/f1000research.15635.2 (PMC6352925; doi:10.12688/f1000research.15635.2)
Supplement: Supplementary file 9 [file f1000research-7-18800-s0007.tgz › f2bd17d0-3d01-4acc-ae3f-e89876bdcf34.pdf]

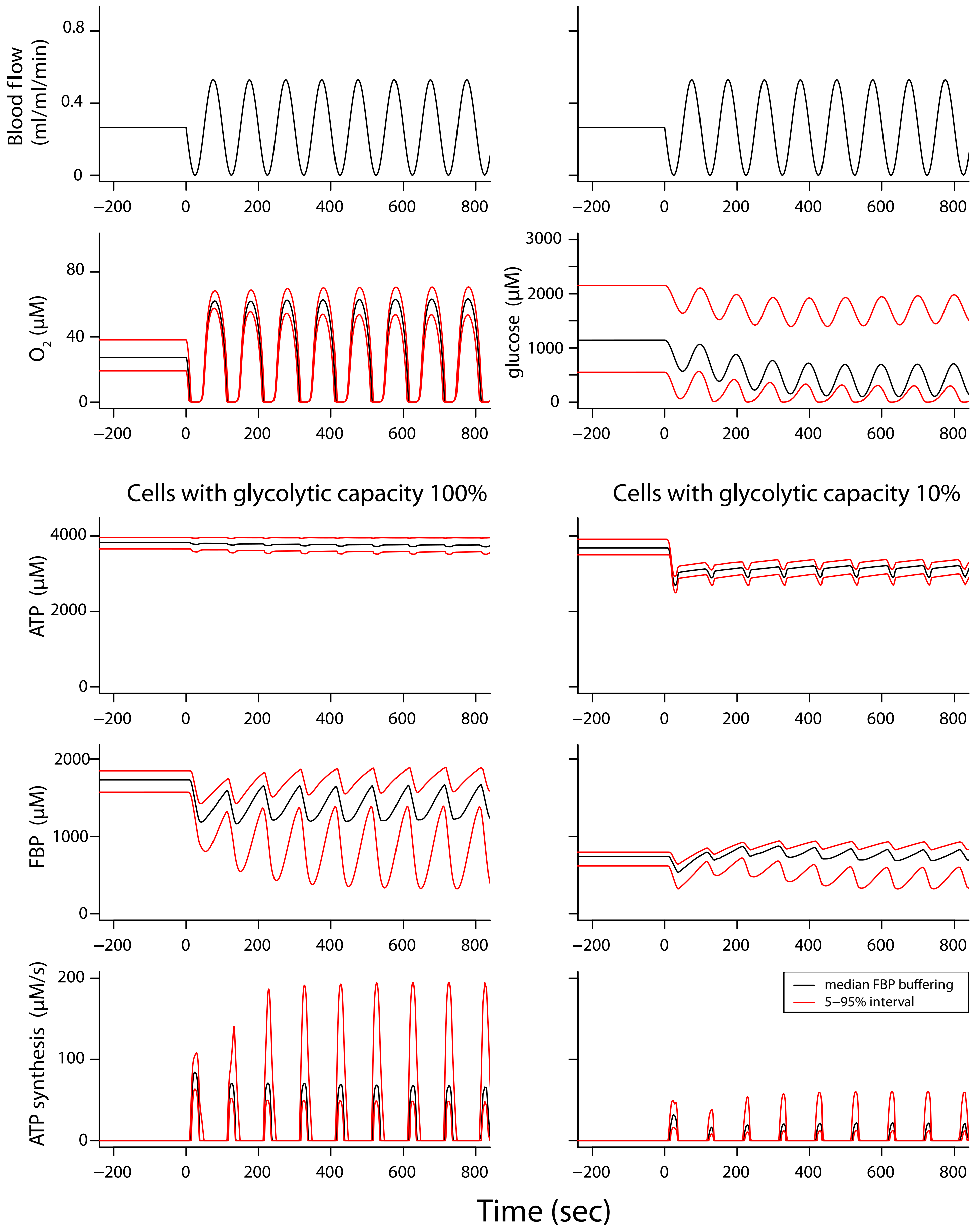

Blood flow (ml/ml/min)
0.8
0.4
0
−200
0
200
400
600
800
O_2 (μM)
80
40
glucose (μM)
3000
2000
1000
Cells with glycolytic capacity 100%
Cells with glycolytic capacity 10%
ATP (μM)
4000
2000
FBP (μM)
1000
ATP synthesis (μM/s)
200
100
median FBP buffering
5–95% interval
Time (sec)
