# Supplementary figures and images for: The dynamic side of the Warburg effect: glycolytic intermediate storage as buffer for fluctuating glucose and O 2 supply in tumor cells
Source: F1000Res. 2018 Dec 28;7:1177. Originally published 2018 Aug 2. [Version 2] doi: 10.12688/f1000research.15635.2 (PMC6352925; doi:10.12688/f1000research.15635.2)

92  $\mu\text{M}$  glucose added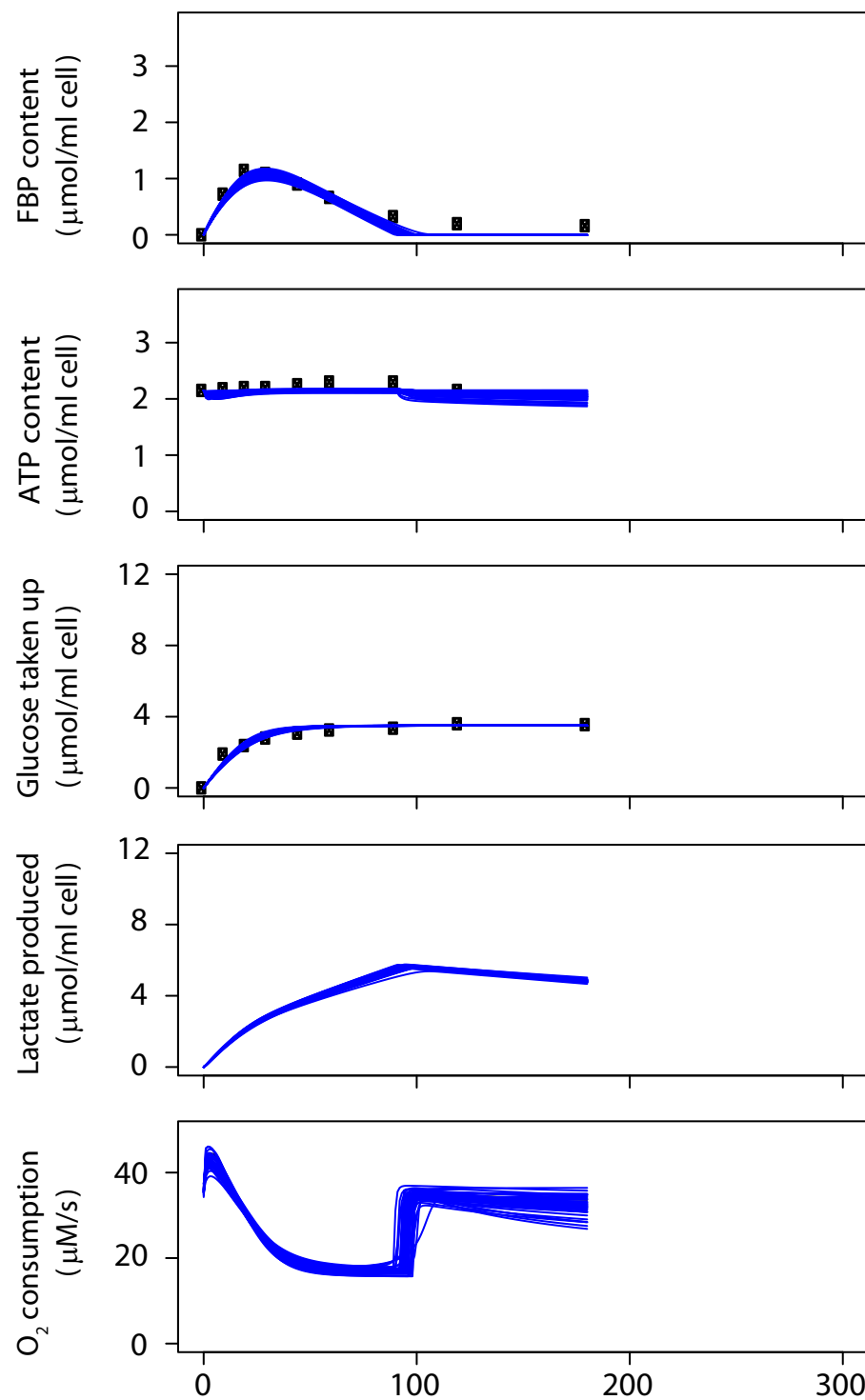776  $\mu\text{M}$  glucose added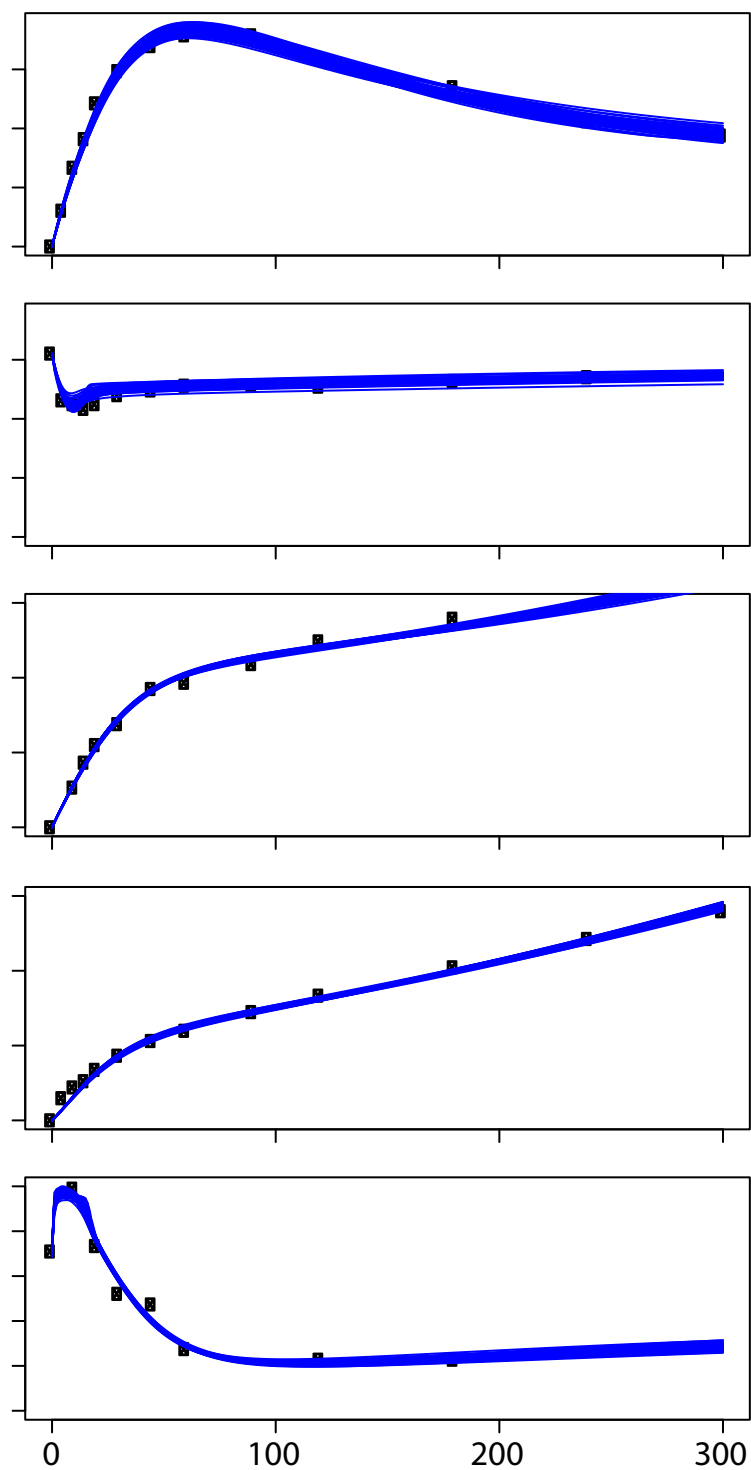

Time (sec)

Supplement: Supplementary file 10 [file f1000research-7-18800-s0008.tgz › da07357d-3de9-4b21-bfe2-d03c0cfb5cf8.pdf]

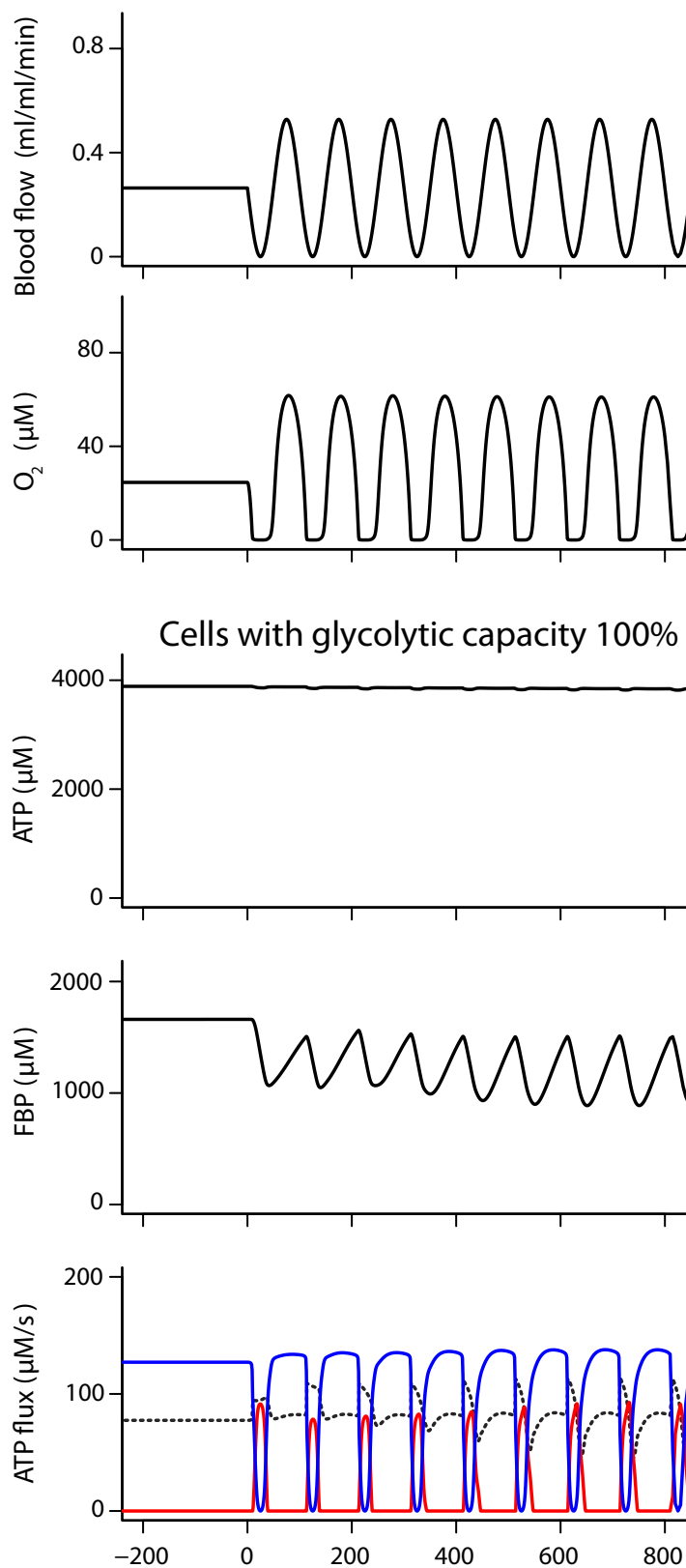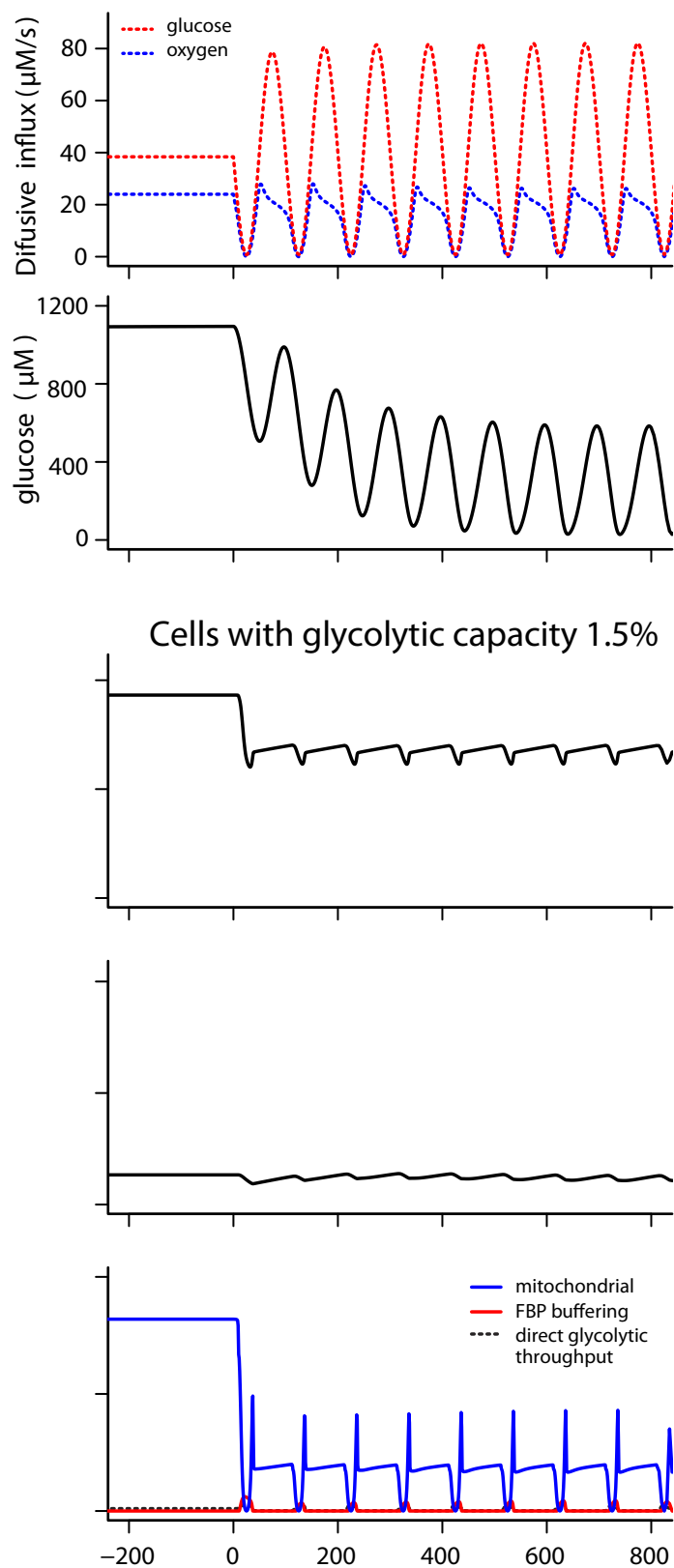

Time (sec)

Supplement: Supplementary file 11 [file f1000research-7-18800-s0009.tgz › 28f83cde-854c-440a-8839-59ed04c9ae08.pdf]

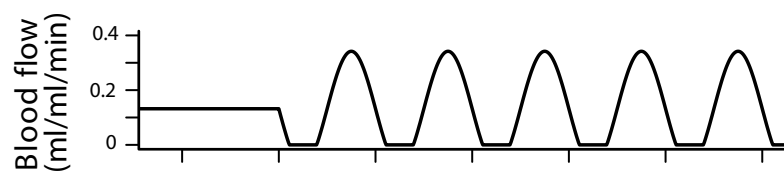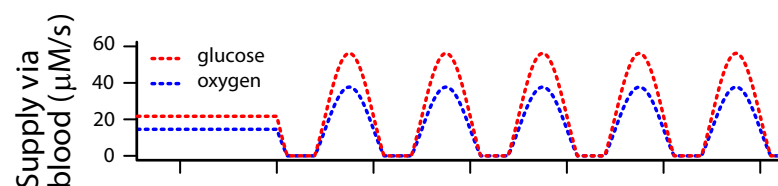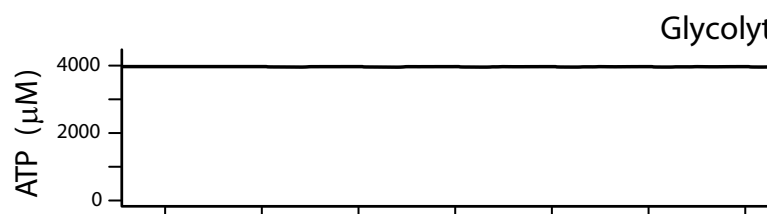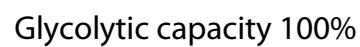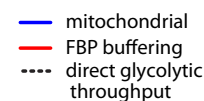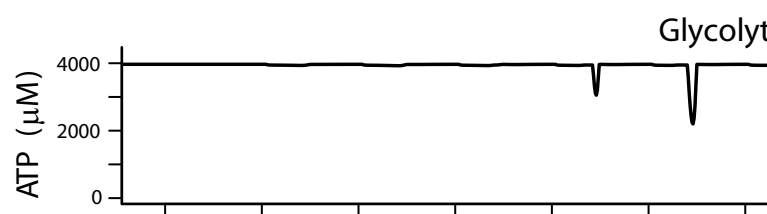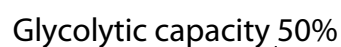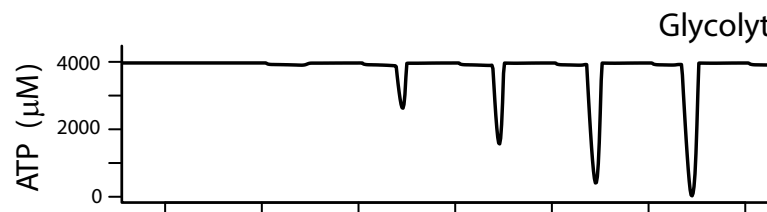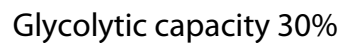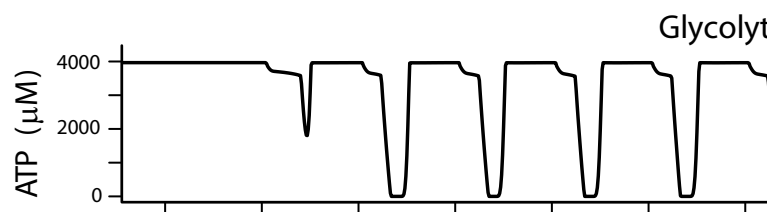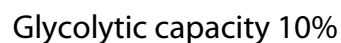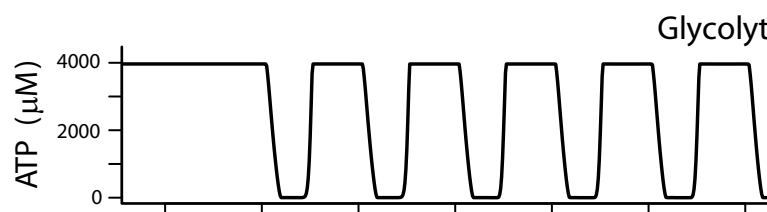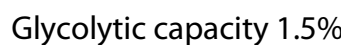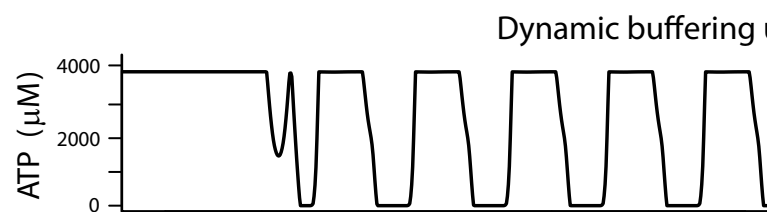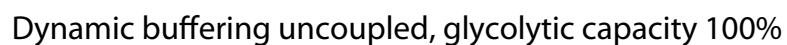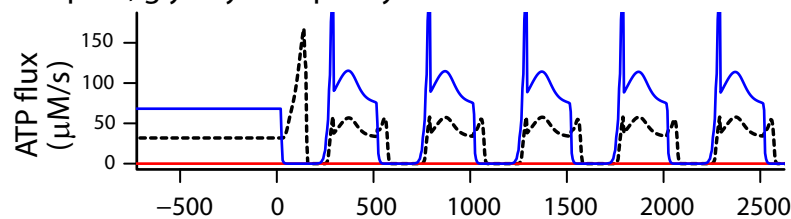

Supplement: Supplementary file 12 [file f1000research-7-18800-s0010.tgz › 3345e070-5929-4310-b67a-3c5a7e2d8573.pdf]
