# Supplementary material for: The dynamic side of the Warburg effect: glycolytic intermediate storage as buffer for fluctuating glucose and O 2 supply in tumor cells
Source: F1000Res. 2018 Dec 28;7:1177. Originally published 2018 Aug 2. [Version 2] doi: 10.12688/f1000research.15635.2 (PMC6352925; doi:10.12688/f1000research.15635.2)
Supplement: Supplementary file 13 [file f1000research-7-18800-s0011.tgz › afa3ca25-da53-4d1c-b2e9-aa0147cbed9d.docx]

**Supplementary Table 1. State variables in the model of tumor cell metabolism.**

| **Variable** | **Description** | **Unit** | **Remarks** |
| --- | --- | --- | --- |
| Metabolic model | | | |
| glucose | glucose concentration | µmol/liter | distributed in cytosol and extracellular compartment, amount per liter H_2_O |
| lac | lactate concentration | µmol/liter | cytosol and extracellular |
| pyr | pyruvate concentration | µmol/liter | cytosol and extracellular |
| ATP | adenosine triphosphate concentration | µmol/liter | cytosol |
| ADP | adenosine diphosphate concentration | µmol/liter | cytosol |
| FBP | fructose 1,6-bisphosphate concentration | µmol/liter | cytosol |
| F_active_ | active fraction of the head section of glycolysis | fraction | cytosol |
| NAD | nicotinamide adenine dinucleotide (oxidized) | µmol/liter | cytosol |
| NADH | nicotinamide adenine dinucleotide (reduced) | µmol/liter | cytosol |
| O_2_ | oxygen | µmol/liter | cytosol and extracellular |
| PGI | phosphorylated glycolytic intermediates | µmol/liter | cytosol, PGI=R_PGI/FBP_∙FBP |
| Tissue O_2_ transport model | | | |
| O_2,perivascular_ | oxygen concentration near blood vessel | µmol/liter |  |
| c_a,O2_ | arterial oxygen concentration | µmol/liter | free oxygen, excluding oxygen bound to hemoglobin |
| c_v,O2_ | venous oxygen concentration | µmol/liter | free oxygen |
| M_perivascular_ | concentration metabolite M near blood vessel | µmol/liter | M = glucose, lactate or pyruvate |
| c_M,a_ | arterial concentration of metabolite | µmol/liter | M = glucose, lactate or pyruvate |
| c_M,v_ | venous concentration of metabolite M | µmol/liter | M = glucose, lactate or pyruvate |
| M | concentration of metabolite M in tissue | µmol/liter | M = glucose, lactate or pyruvate |
