# Supplementary material for: The dynamic side of the Warburg effect: glycolytic intermediate storage as buffer for fluctuating glucose and O 2 supply in tumor cells
Source: F1000Res. 2018 Dec 28;7:1177. Originally published 2018 Aug 2. [Version 2] doi: 10.12688/f1000research.15635.2 (PMC6352925; doi:10.12688/f1000research.15635.2)
Supplement: Supplementary file 14 [file f1000research-7-18800-s0012.tgz › 3bb8fc66-56ba-4058-b27a-3a01b3687859.docx]

**Supplementary Table 2. Metabolic fluxes in the model of tumor cell metabolism.**

| **Flux** | **Description** | **Unit** | **Remarks** |
| --- | --- | --- | --- |
| Metabolic model – biochemical conversion | | | |
| J_head_ | flux in head section of glycolysis | µmol 6-carbon units/liter total intracellular H_2_O/s | glycolytic flux in section from glucose to FBP |
| J_tail_ | flux in tail section of glycolysis | µmol 3-carbon units/liter total intracellular H_2_O/s | glycolytic flux in section from FBP to pyruvate |
| J_mit_ | mitochondrial ATP synthesis | µmol /liter total intracellular H_2_O/s |  |
| J_lac_ | lactate dehydrogenase flux | µmol /liter total intracellular H_2_O/s | pyruvate to lactate conversion |
| J_hyd_ | ATP hydrolysis | µmol /liter total intracellular H_2_O/s | by all cellular processes that use ATP except the head section of glycolysis |
| J_breakdown,ADP_ | breakdown of ADP | µmol /liter total intracellular H_2_O/s | ADP to AMP and subsequently to adenosine, inosine, hypoxanthine etc. (*1, 2*) |
| J_synth,ADP_ | ADP synthesis | µmol /liter total intracellular H_2_O/s | adenine nucleotide synthesis from adenosine, inosine, hypoxanthine etc. and de novo |
| J_store_ | metabolite storage taken from glycolytic intermediates | µmol 6-carbon units/liter total intracellular H_2_O/s | carbon-containing metabolites (e.g. nucleosides, glycogen, serine, etc.) |
| J_pyr,mit_ | pyruvate metabolism by mitochondria | µmol /liter total intracellular H_2_O/s | pyruvate consumed by mitochondrial oxidative metabolism |
| J_nadh,mit_ | NADH used by mitochondria | µmol /liter total intracellular H_2_O/s | NADH taken up for oxidative phospho-rylation via reducing equivalent shuttles |
| J_ATP,glyc_ | net glycolytic ATP synthesis | µmol /liter total intracellular H_2_O/s | $V_{ATP, glyc}=2V_{tail }-2V_{head}$ |
| Tissue model – diffusive fluxes | | | |
| J_diff,O2_ | diffusion of oxygen per liter intracellular H_2_O | µmol / liter intracellular H_2_O / s | net diffusion into tissue layer |
| J_diff,M_ | diffusion of metabolite M per liter intracellular H_2_O | µmol / liter intracellular H_2_O / s | M = glucose, lactate or pyruvate |

**References**

1. G. Glaser, H. Giloh, J. Kasir, M. Gross, J. Mager, On the mechanism of the glucose-induced ATP catabolism in ascites tumour cells and its reversal by pyruvate. *Biochem J* **192**, 793-800 (1980).

2. K. Overgaard-Hansen, Metabolic regulation of the adenine nucleotide pool. I. Studies on the transient exhaustion of the adenine nucleotides by glucose in Ehrlich ascites tumor cells. *Biochim Biophys Acta* **104**, 330-347 (1965).
