# Supplementary material for: The dynamic side of the Warburg effect: glycolytic intermediate storage as buffer for fluctuating glucose and O 2 supply in tumor cells
Source: F1000Res. 2018 Dec 28;7:1177. Originally published 2018 Aug 2. [Version 2] doi: 10.12688/f1000research.15635.2 (PMC6352925; doi:10.12688/f1000research.15635.2)
Supplement: Supplementary file 15 [file f1000research-7-18800-s0013.tgz › 3809938c-1766-4e94-b3b6-11adfe5738df.docx]

**Supplementary Table 3. Parameters in the model of tumor cell metabolism.**

| **Parameter** | **Description** | **Value** | **Unit** | **Remarks** |
| --- | --- | --- | --- | --- |
| Metabolic Model | | | | |
| V_cyt_ | volume cytosolic H_2_O per total intracellular H_2_O | 0.9 | liter/liter intracellular H_2_O | references (in parentheses):  (*1*) |
| V_mat_ | volume mitochondrial H_2_O per total intracellular H_2_O | 0.1 | liter/liter intracellular H_2_O | (*1*) |
| V_ext_ | volume extracellular H_2_O per total intracellular H_2_O |  | liter/liter intracellular H_2_O | depends on experimental conditions |
| Head section glycolysis | | | | |
| K_ATP,head_ | Michaelis constant, K_m,_ of head section glycolysis for ATP | 1000 | µmol/liter | (*2-4*) |
| K_glucose,head_ | K_m_ of head for glucose | 51.1 (10.8) | µmol/liter | (*) optimized  (interquartile range: IQR); compare with (*2-4*) |
| V_max,head_ | maximal forward rate of enzyme, V_max_ | 394.3 (13.5) | µM/s | (*) optimized value  (IQR) |
| Tail section glycolysis | | | | |
| K_FBP,tail_ | K_m_ for FBP | 0.24 (1.06) | µmol/liter | (*) optimized (IQR); compare (*2-4*) |
| K_NAD,tail_ | K_m_ for NAD | 33.5 (36.2) | µmol/liter | (*) optimized (IQR); compare (*2-4*) |
| K_ADP,tail_ | K_m_ for ADP | 220.4 (128.7) | µmol/liter | (*) optimized (IQR); compare (*2-4*) |
| K_i,NADH,tail_ | inhibitory constant for NADH | 2.77 (6.6) | µmol/liter | (*) optimized (IQR); compare (*2-4*) |
| V_max,tail_ | Vmax of tail section | 1708 (438) | µM/s | (*) optimized (IQR) |
| Mitochondrial ATP synthesis - oxidative phosphorylation | | | | |
| K_ADP,mit_ | K_m_ for ADP | 22.1 (41.8) | µmol/liter | (*) optimized (IQR); compare (*5-9*) |
| K_Pi,mit_ | K_m_ for P_i_ | 800 | µmol/liter | (*5-9*) |
| K_O2,mit_ | K_m_ for O_2_ | 0.26 | µmol/liter | (*10*) |
| V_synmax_ | maximal rate of mitochondrial ATP synthesis | 306.8 (14.7) | µM/s | (*) optimized (IQR) |
| Lactate dehydrogenase reaction | | | | |
| K_pyr,ldh_ | K­_m_ for pyruvate | 335 | µmol/liter | (*3*) |
| K_NADH,ldh_ | K_m_ for NADH | 2 | µmol/liter | (*3*) |
| K_lac,ldh_ | K_m_ for lactate | 17000 | µmol/liter | (*3*) |
| K_NAD,ldh_ | K_m­_ for NAD | 849 | µmol/liter | (*3*) |
| K_eq,ldh_ | equilibrium constant for the lactate dehydrogenase reaction | 16198 | dimensionless | (*3*) |
| V_max,ldh,for_ | maximal rate lactate dehydrogenase reaction: forward from pyruvate to lactate | 4939 | µM/s | (*11, 12*) |
| ATP hydrolysis for cellular processes (except phosphorylation of glucose) | | | | |
| k_hyd,AdN_ | reduction of glycolysis per unit decrease in sum of ATP and ADP concentrations | 0.166 (0.030) | s^-1^ | (*) optimized (IQR);  in vitro (Fig. 2) and tissue (Figs. 3, 4, S1-S3)  S=Supplementary Figure |
| V_max,hyd_ | ATP hydrolysis under basal conditions | 199.0 (3.8) | µM/s | (*) optimized (IQR); simulation Fig. 2, Figs. 3, 4, S1-S3. |
| ATP hydrolysis at low adenine nucleotide levels, Eq. 6 (for tissue simulations) | | | | |
| AdN_cutoff_ | AdN cutoff value; boundary between Eqs. 5 and 6 | 3189 | µM | simulations Figs. 3, 4 and S3 |
| k_hyd,AdN,low_ | linear part Eq. 6 | 0.0098704 | 1/s | simulations Figs. 3, 4 and S1-S3 |
| C_power_ | coefficient power law | 9.73 10^-47^ |  | simulations Figs. 3, 4 and S1-S3 |
| γ | power coefficient in Eq. 6 | 13.58 |  | simulations Figs. 3, 4 and S1-S3 |
| ADP breakdown and synthesis | | | | |
| k_breakdown,ADP_ | rate constant for breakdown of ADP | 0.000224  (8.3 10^-5^) | µM^-1^.s^-1^ | (*) optimized  (IQR) |
| k_synthesis,ADP_ | rate constant for synthesis of ADP | 0.001261 (0.00028) | s^-1^ | (*) optimized  (IQR) |
| Storage carbon metabolites from glycolytic intermediates | | | | |
| k_store_ | rate constant for storage of carbon metabolites | 0.005202 (0.00043) | s^-1^ | (*) optimized (IQR) |
| Parameters algebraic equations | | | | |
| NAD_tot_ | total nicotinamide adenine dinucleotide content | 200 | µM | (*13, 14*) |
| ratio_P/O2_ | ratio of ATP synthesized per O_2_ consumed | 5.6 | dimensionless | (*15-17*) |
| R_PGI/FBP_ | ratio of total phosphorylated glycolytic intermediates content to fructose 1,6-bisphosphate content | 1.243 (0.047) | dimensionless | (*) optimized (IQR);  total phosphorylated glycolytic intermediates varying with fixed ratio to FBP;  other stores of metabolites increase in time (Eq. 9) and are not proportional to FBP. Cf. (*13, 16, 18-24*) |
| Inhibition of head section of glycolysis | | | | |
| k_f_ | second order forward rate constant for inhibition by glycolytic intermediates | 1.01∙10^-5­^  (7.5 10^-7^) | µM^-1^s^-1^ | (*) optimized (IQR) |
| k_b_ | backward rate constant for disinhibition of head section | 0.00315 (0.00034) | s^-1^ | (*) optimized (IQR) |
|  | | | | |
| Transport equations of tissue model | | | | |
| Geometric parameters | | | | |
| L_cyl_ | length of cylindrical shell per unit volume of intracellular H_2_O |  | cm / liter | calculated from tissue radius, intracellular water content and interstitial space |
| r­_inner_ | inner radius of cylindrical shell |  | cm |  |
| r_outer_ | outer radius of cylindrical shell |  | cm |  |
| V_perivascular_ | volume of blood vessel and immediately adjacent tissue per volume of intracellular H_2_O | 0.04 | liter / liter | (*25*) |
| r_perivascular_ | radius of the perivascular space | 9.5 | µm | (*26*); compatible with V_perivascular_ |
| volume fraction cells | volume consisting of cells as fraction of total tissue volume | 0.50 | liter / liter | tumor tissue  (*27, 28*) |
| Cell types | | | | |
| f_cell type 1_ | fraction of total cell volume that consists of cells with full tumor glycolytic capacity |  |  |  |
| f_cell type 2_ | fraction of total cell volume that consists of cells with reduced glycolytic capacity |  |  |  |
| Diffusion coefficients | | | | |
| D_O2_ | diffusion coefficient of oxygen | 1.75∙10^-5^ | cm^2^ s^-1^ | (*26, 29, 30*) |
| D_glc_ | diffusion coefficient of glucose | 0.5∙10^-5^ | cm^2^ s^-1^ | (*26, 30, 31*) |
| D_lac_ | diffusion coefficient of lactate | 0.9∙10^-5^ | cm^2^ s^-1^ | (*26, 32*) |
| D_pyr_ | diffusion coefficient of pyruvate | 0.9∙10^-5^ | cm^2^ s^-1^ | (*26, 32*) |
| Blood flow and concentrations | | | | |
| F_blood_ | blood flow per liter intracellular H_2_O | 0.0044 | liter blood / liter intracellular H_2_O / s | (*25*) |
| c­_a,glucose_ | arterial glucose concentration | 9858 | µmol/L | for implanted tumor experiments in rats (*33*) |
| c_a,lactate_ | arterial lactate concentration | 3108 | µmol/L | (*33*) |
| c_a,pyruvate_ | arterial pyruvate concentration | 144 | µmol/L | (*33*) |
| c_a,O2_ | arterial unbound oxygen concentration | 120.8 | µmol/L | (*33*) |
| Oxygen dissociation curve blood | | | | |
| c_sat,HbO2_ | concentration of oxygen carried by saturated hemoglobin | 7350 | µM | (*33*) |
| n_Hill_ | Hill coefficient | 2.6 | dimensionless | (*34*) |
| c_50,O2_ | O_2_ concentration at which hemoglobin is 50% saturated with O_2_ | 52 (arterial)  60 (venous) | µM | hemoglobin half saturation value rat blood(*34*):  p_50_ = 39.3 mmHg at pH = 7.34 (arterial) (*33*); p50 = 45.5 mmHg at pH = 7.24 (venous) ;  O_2_ solubility = 1.33 µM/mmHg (*35*) |
| (*) these parameters are optimized on data from experiment 1-3, see description in Supplementary Material: Calibrating the computational model with experimental data. Interquartile range (IQR) determined by replicated Markov chain Monte Carlo is given for each optimized parameter. | | | | |

**References**

1. B. Chance, D. Garfinkel, J. Higgins, B. Hess, A solution for the equations representing interaction between glycolysis and respiration in ascites tumor cells. *Journal of Biological Chemistry* **235**, 2426-2439 (1960).

2. P. J. Mulquiney, P. W. Kuchel, Model of 2,3-bisphosphoglycerate metabolism in the human erythrocyte based on detailed enzyme kinetic equations: equations and parameter refinement. *Biochem J* **342**, 581-596 (1999).

3. M. J. Lambeth, M. J. Kushmerick, A computational model for glycogenolysis in skeletal muscle. *Annals of Biomedical Engineering* **30**, 808-827 (2002).

4. A. Marin-Hernandez, J. C. Gallardo-Perez, S. Rodriguez-Enriquez, R. Encalada, R. Moreno-Sanchez, E. Saavedra, Modeling cancer glycolysis. *Biochim Biophys Acta* **1807**, 755-767 (2011).

5. H. Hettling, J. H. van Beek, Analyzing the functional properties of the creatine kinase system with multiscale 'sloppy' modeling. *PLoS Comput Biol* **7**, e1002130 (2011).

6. J. H. van Beek, Adenine nucleotide-creatine-phosphate module in myocardial metabolic system explains fast phase of dynamic regulation of oxidative phosphorylation. *Am J Physiol Cell Physiol* **293**, C815-829 (2007).

7. O. Kongas, T. L. Yuen, M. J. Wagner, J. H. Van Beek, K. Krab, High K(m) of oxidative phosphorylation for ADP in skinned muscle fibers: where does it stem from? *Am J Physiol Cell Physiol* **283**, C743-751 (2002).

8. C. D. Stoner, H. D. Sirak, Steady-state kinetics of the overall oxidative phosphorylation reaction in heart mitochondria. *Journal of Bioenergetics and Biomembranes* **11**, 113-146 (1979).

9. F. W. Heineman, R. S. Balaban, Phosphorus-31 nuclear magnetic resonance analysis of transient changes of canine myocardial metabolism in vivo. *J Clin Invest* **85**, 843-852 (1990).

10. G. Froese, The respiration of ascites tumor cells at low oxygen concentration. *Biochimica en Biophysica Acta* **57**, 509-519 (1962).

11. R. Wu, E. Racker, Regulatory mechanisms in glycolysis of ascites tumor cells: III. Limiting factors in glycolysis of ascites tumor cells. *Journal of Biological Chemistry* **234**, 1029-1035 (1959).

12. K. Brand, K. Deckner, J. Musil, Enzyme pattern of pentose phosphate pathway in ascites tumor cells and the effect of nucleoside triphosphates on its enzyme activities. *Hoppe-Seyler's Z. Physiol. Chem.* **351**, 213-220 (1970).

13. E. L. Coe, Correlations between adenine nucleotide levels and the velocities of rate-determining steps in the glycolysis and respiration of intact Ehrlich ascites carcinoma cells. *Biochimica et Biophysica Acta* **118**, 495-511 (1966).

14. K. H. Ibsen, E. L. Coe, R. W. McKee, Interrelationships of metabolic pathways in the Ehrlich ascites carcinoma cells. I. Glycolysis and respiration (Crabtree effect). *Biochimica en Biophysica Acta* **30**, 384-400 (1958).

15. E. L. Coe, I.-Y. Lee, Phosphorylation of 2-D-deoxyglucose and associated inorganic phosphate uptake in ascites tumor cells. *Biochemistry* **8**, 685-693 (1969).

16. E. L. Coe, Correlation of glycolytic and respiratory events after addition of a small amount of glucose to Ehrlich ascites carcinoma. *Cancer Research* **26**, 269-275 (1966).

17. B. Hess, B. Chance, Phosphorylation efficiency of the intact cell. I. Glucose-oxygen titrations in ascites tumor cells. *234*, 3031-3035 (1959).

18. K. A. Gumaa, P. McLean, The pentose phosphate pathway of glucose metabolism. Enzyme profiles and transient and steady-state content of intermediates of alternative pathways of glucose metabolism in Krebs ascites cells. *Biochem J* **115**, 1009-1029 (1969).

19. K. K. Lonberg, A direct study of intracellular glycolysis in Ehrlich's ascites tumor. *Biochimica et Biophysica Acta* **35**, 464-472 (1959).

20. B. Hess, B. Chance, Metabolic control mechanisms. VI. Chemical events after glucose addition to ascites tumor cells. *Journal of Biological Chemistry* **236**, 239-246 (1961).

21. K. A. Gumaa, P. McLean, Sequential control of hexokinase in ascites cells. *Biochemical and Biophysical Research Communications* **35**, 824-831 (1969).

22. G. Wilhelm, J. Schulz, E. Hoffmann, pH-Abhängigkeit von Glykolyse und Atmung in Ehrlich-Ascitestumorzellen. *Acta Biologica et Medica Germanica* **29**, 1-16 (1972).

23. E. L. Coe, K. H. Ibsen, M. Dixon, R. W. McKee, Glycolysis of small amounts of glucose by Ehrlich ascites carcinoma cells. *Cancer Research* **26**, 276-281 (1966).

24. I.-L. Lee, R. C. Strunk, E. L. Coe, Coordination among rate-limiting steps of glycolysis and respiration in intact ascites tumor cells. *Journal of Biological Chemistry* **242**, 2021-2028 (1967).

25. P. Vaupel, Hypoxia in neoplastic tissue. *Microvascular Research* **13**, 399-408 (1977).

26. I. F. Tannock, The relation between cell proliferation and the vascular system in a transplanted mouse mammary tumour. *British Journal of Cancer* **22**, 258- (1968).

27. H. Wiig, M. A. Swartz, Interstitial fluid and lymph formation and transport: physiological regulation and roles in inflammation and cancer. *Physiol Rev* **92**, 1005-1060 (2012).

28. P. M. Gullino, F. H. Grantham, S. H. Smith, The interstitial water space of tumors. *Cancer Research* **25**, 727-731 (1965).

29. J. Grote, R. Susskind, P. Vaupel, Oxygen diffusivity in tumor tissue (DS-carcinosarcoma) under temperature conditions within the range 20-40^0^C. *Pflugers Arch* **372**, 37-42 (1977).

30. M. Robertson-Tessi, R. J. Gillies, R. A. Gatenby, A. R. Anderson, Impact of metabolic heterogeneity on tumor growth, invasion, and treatment outcomes. *Cancer Res* **75**, 1567-1579 (2015).

31. K. Groebe, S. Erz, W. Mueller-Klieser, Glucose diffusion coefficients determined from concentration profiles in Emt6 tumor spheroids incubated in radioactively labeled L-glucose. *Advances in Experimental Medicine and Biology* **361**, 619-625 (1994).

32. X. Zhang, C.-G. Li, C.-H. Ye, M.-L. Liu, Determination of molecular self-diffusion coefficient using multiple spin-echo NMR spectroscopy with removal of convection and background gradient artifacts. *Analytical Chemistry* **73**, 3528-3534 (2001).

33. P. Vaupel, H. Günther, J. Grote, Atemgaswechsel und Glucosestoffwechsel von tumoren (DS-Carcinosarkom) in vivo. I. Experimentelle Untersuchungen der versorgungsbestimmenden parameter. *Z ges exp Med* **156**, 283-294 (1971).

34. C.-F. Cartheuser, Standard and pH-affected hemoglobin-O2 binding curves of Sprague-Dawley rats under normal and shifted P50 conditions. *Comp Biochem Physiol* **106A**, 775-782 (1993).

35. O. Siggaard-Andersen, *The Acid-base Status of the Blood*. (Munksgaard, Copenhagen, ed. Fourth Edition, 1976).
