# Supplementary material for: The dynamic side of the Warburg effect: glycolytic intermediate storage as buffer for fluctuating glucose and O 2 supply in tumor cells
Source: F1000Res. 2018 Dec 28;7:1177. Originally published 2018 Aug 2. [Version 2] doi: 10.12688/f1000research.15635.2 (PMC6352925; doi:10.12688/f1000research.15635.2)
Supplement: Peer review history

# Peer review: Referee response for version 2

**DOI:** 10.5256/f1000research.18800.r42232

**Published:** 2019-01-28

**Authors:** Ziwei Dai

**Affiliations:** 1 Duke University School of Medicine, Durham, NC, USA

The author has successfully addressed my comments.

I have read this submission. I believe that I have an appropriate level of expertise to confirm that it is of an acceptable scientific standard.

# Peer review: Referee response for version 1

**DOI:** 10.5256/f1000research.17060.r37981

**Published:** 2018-09-14

**Authors:** Ziwei Dai

**Affiliations:** 1 Duke University School of Medicine, Durham, NC, USA

The manuscript ‘The dynamic side of the Warburg effect: glycolytic intermediates as buffer for fluctuating glucose and O2 supply in tumor cells’ presents a coarse-grained kinetic model of glycolysis. The model, despite being extremely simplified, is able to reproduce several experimental datasets in Ehrlich ascites tumor cells after parameter calibration. The author then applies this model to predict dynamic behaviors of tumor cells under fluctuating blood flow. I like the idea of using simplified models to interpret complex biological phenomena but would like to see more discussions about the rationale of using this model instead of other models with full details of every reaction involved in glycolysis. There is space to improve presentation of the results as well. Specific comments are listed below for the author’s reference.

1. Some important interactions that may affect FBP dynamics are missing in the model. For instance, it is known that FBP is an allosteric activator of pyruvate kinase (Jurica et al ^1^; Christofk et al ^2^). I expect that this feedback will attenuate the FBP buffering mechanism proposed in this study since lower glycolysis (i.e. 'tail' in this model) is activated by high concentration of FBP thus enhancing its consumption. Moreover, the two sections (‘head’ and ‘tail’) of glycolysis are both considered to be irreversible, thus neglecting the effects of thermodynamics on glycolytic flux. This may also lead to overestimation of FBP concentration as well because FBP is not allowed to be converted back to glucose.
2. The main goal of developing this coarse-grained model is not clear to me. Besides the model developed by Chance and Hess, there are numerous mathematical models for glycolysis, most are much more detailed than the model presented in this study. The author claims that this model replaces the model of Chance and Hess – I feel this inappropriate since this statement doesn’t give any credit to all other glycolysis models.
3. Most, if not all, results are presented as curves from the simulation with little information about the take-home messages. It is thus very difficult to read the key findings directly from the figures. For instance, it is my understanding that one of the two most important hypotheses drawn based on the simulation is that cells with high glycolytic capacity (which mimics ‘Warburgian’ cancer cells) consume glucose much more quickly than cells with lower glycolytic capacity after switching from low glucose to high glucose condition, thus being more competitive under conditions with frequent nutrient deprivation. To emphasize this point, I would recommend using one figure directly comparing glucose uptake fluxes in cells with different glycolytic capacities instead of the 10 figure panels currently included in Fig 4.  

I have read this submission. I believe that I have an appropriate level of expertise to confirm that it is of an acceptable scientific standard, however I have significant reservations, as outlined above.

## References

[1] JuricaMSMesecarAHeathPJShiWNowakTStoddardBL: The allosteric regulation of pyruvate kinase by fructose-1,6-bisphosphate. *Structure*.1998;6(2) :195-210 951941010.1016/s0969-2126(98)00021-5

[2] ChristofkHRVander HeidenMGWuNAsaraJMCantleyLC: Pyruvate kinase M2 is a phosphotyrosine-binding protein. *Nature*.2008;452(7184) : 10.1038/nature06667 181-6 10.1038/nature06667 18337815

# Response

**Published:** 2018-12-10

**Authors:** Hans van Beek

**Affiliations:** Department of Experimental Vascular Medicine, Academic Medical Center of the University of Amsterdam, The Netherlands

Answer to Reviewer Ziwei Dai

*… There is space to improve presentation of the results as well. Specific comments are listed below for the author’s reference.*

 Thank you for your constructive criticism. Your point is well taken and I followed your suggestions. Your remarks were very helpful to improve the presentation, as specified below.

*Some important interactions that may affect FBP dynamics are missing in the model. For instance, it is known that FBP is an allosteric activator of pyruvate kinase (Jurica et al ; Christofk et al ). I expect that this feedback will attenuate the FBP buffering mechanism proposed in this study since lower glycolysis (i.e. 'tail' in this model) is activated by high concentration of FBP thus enhancing its consumption.*

 Equation 2 of the model implies that the flux in the tail section increases with FBP. This reflects not only that FBP is a substrate for the tail end of glycolysis (via aldolase), but also reflects a potential activating role of FBP on pyruvate kinase. To examine multiple roles of FBP, I tested various forms of the mathematical relation between FBP and flux in the tail section, among others by using powers of FBP (Hill coefficient), but these attempts did not improve simulations. Note that the tail flux turns out to be sensitive to extremely low levels of FBP, with K _FBP,tail_ estimated to be 0.24 μM, a level that is reached almost immediately after reintroduction of glucose in the experiment of Fig. 2A (see Dataset 1). This agrees with activation of pyruvate kinase by FBP in the micromolar range ( *1*). This also agrees with the very fast response of lactate production immediately after reintroduction of glucose, found experimentally and reproduced by the model (Figures 2A and 2B). The activating effect of FBP on tail flux is therefore taken into account in the model. I have added discussion of this point and the references you gave to Jurica and Christofk to the main text  (Methods, seventh paragraph) and Supplementary Text (paragraph around Eq. 2).

*Moreover, the two sections (‘head’ and ‘tail’) of glycolysis are both considered to be irreversible, thus neglecting the effects of thermodynamics on glycolytic flux. This may also lead to overestimation of FBP concentration as well because FBP is not allowed to be converted back to glucose.*

 Your fellow reviewer, Dr Dash gave a similar comment. I have therefore addressed this in a joint answer which is also included in the answer to Dr Dash:

 Glycolysis is considered by biochemists to be essentially irreversible, in particular the hexokinase, phosphofructokinase and pyruvate kinase reactions (see for instance the textbook by Berg, Tymocko, Stryer. Biochemistry, Sixth Edition, 2007, e.g. page 460, ref. ( *2*)). In addition the glyceraldehyde 3-phosphate dehydrogenase reaction may also be an important limiting step in cells showing the Warburg effect ( *3, 4*). To produce glucose in the reverse direction (fructose 1,6-bisphosphate to glucose) the enzyme glucose 6-phosphatase is required to bypass the irreversible hexokinase reaction. Glucose 6-phosphatase’s activity in Ehrlich ascites cells is low ( *5*), while its activity in the liver, which can produce glucose, is high. The tail section of glycolysis is also considered to be virtually irreversible and two additional enzymes (pyruvate carboxylase and phoshoenolpyruvate carboxykinase) are required to enable conversion of pyruvate in the direction of glucose, bypassing pyruvate kinase (see ( *2*), p. 460-463).

   I had implemented two existing detailed models of the glycolytic chain and used these to explore the reversibility of the reactions. The first model was based on the model of Lambeth and Kushmerick ( *6*), extended with the equation for hexokinase given by Mulquiney and Kuchel ( *7*). The second model was based on the model of Marin-Hernandez et al. ( *8*) for cancer cells. Both models represent the ten enzymatic steps in the glycolytic chain and incorporates both the forward and reverse reactions for all the steps. All these equations take the Haldane relation into account, and constraints based on thermodynamics are obeyed. Simulations with both models showed that at least one reaction in the head as well as the tail section of the glycolytic chain showed reverse fluxes that were extremely small relative to the forward fluxes. The results are discussed in more detail in the revised Supplementary Text, section titled “Effect of Reverse Fluxes on the Glycolytic Model”.

   These simulations illustrate the biochemical concept that glycolysis is essentially irreversible. In order to reduce the model as much as possible, the reverse reactions in glycolysis were therefore neglected. However, the lactate dehydrogenase reaction is included in fully reversible form and lactate is taken up for mitochondrial metabolism if glucose is depleted in the simulations. Mitochondrial oxidative phosphorylation coupled to oxygen consumption is also modeled as being essentially irreversible, as generally accepted by biochemists. However, these considerations point to a limitation: the model is not suitable for determining the magnitude of small reversed fluxes in the glycolytic chain under conditions where glycolytic products (pyruvate, NADH, ATP) are high and glucose concentration is very low. Reverse fluxes may be revealed by isotope exchange experiments. These considerations are now included in the Methods section of the main paper (8th paragraph) and the Supplementary Text (section title “Effect of Reverse Fluxes on the Glycolytic Model”).

*The main goal of developing this coarse-grained model is not clear to me. Besides the model developed by Chance and Hess, there are numerous mathematical models for glycolysis, most are much more detailed than the model presented in this study. The author claims that this model replaces the model of Chance and Hess – I feel this inappropriate since this statement doesn’t give any credit to all other glycolysis models.*

 There are indeed many mathematical models for glycolysis. I referred to several of these models in the initial submission: the model by Chance and Hess dating back to 1959 ( *9, 10*), the model of Lambeth and Kushmerick ( *6*) for muscle, the model of Mulquiney and Kuchel ( *7*) for red blood cells and the model of Marin-Hernandez et al. ( *8*) for cancer cells were all referenced. Further the model studies of Van Eunen et al. ( *11*), Chandra et al. ( *12*) and Van Heerden et al. ( *13*) on yeast metabolism were referenced. I now have added additional references to model analyses by Shestov et al. ( *3*) and Liberti et al. ( *4*). Although this is not a comprehensive list, I reckon these references (and the references cited therein) probably give sufficient background on mathematical models of glycolysis.

   I had in fact originally implemented two separate detailed models of glycolysis: one developed specifically for cancer cell metabolism by Marin-Hernandez et al. ( *8*) and another one based on the model of Lambeth and Kushmerick ( *6*), extended with the equation for hexokinase by Mulquiney and Kuchel ( *7*). Both glycolytic models represent all the ten enzymatic steps in the glycolytic chain and incorporate both the forward and reverse reaction for all these steps, taking the Haldane relation and thermodynamic constraints into account. Despite simulation attempts with both detailed models, not all features of the experimental response of Ehrlich ascites cells could be reproduced well. The measured overshoot of fructose 1,6-bisphosphate (FBP) after adding a high concentration of glucose to EATC was for instance not reproduced with these detailed models, even when additional regulatory loops were added.

   The coarse-grained model described in this manuscript performed much better and depends on far fewer unknown parameters than the detailed models which contain about 55 and 70 parameters, respectively, for the glycolytic chain alone. Detailed measurements for all separate glycolytic enzymes in the EATC are not available. In contrast, the system level response is well defined by measurements of key nodes in the system: glucose uptake (input head part of glycolysis), fructose 1,6-bisphosphate (output head part of glycolysis and input tail part of glycolysis), lactate (output tail part of glycolysis), respiration, ATP etc. making parametrization of the coarse-grained model feasible. I therefore employed the course-grained model for the purposes of this study. Because a broad range of experimental results *in vitro* are well represented, the model can subsequently be used to explore the role of metabolism *in vivo* during fluctuating nutrient and oxygen supply.

   I understand that the term ‘replace’ in relation to the model of Chance and Hess may be felt to be inappropriate.  The Chance-Hess model is ingenious and of huge historical interest, because it probably is the first digital model of a biochemical reaction system ever published. This is emphasized in the main text (Introduction, first paragraph) and Supplementary Text (section “Short Description of the Computational Model”). Their goal was to explain the metabolic response of the same Ehrlich ascites tumor cells that were investigated in the present study. The phrase containing ‘replace’ has now been removed from the manuscript. What was meant is simply that a new model was used that represents the metabolic responses of EATC to glucose and oxygen levels well and does not contain biochemically untenable assumption. I had in fact also implemented the original model of glycolysis and oxidative phosphorylation by Chance and Hess developed to explain their data on Ehrlich ascites tumor cells, which appeared unsuitable for accurate description of the experimental data of Figure 2A. One key assumption in the Chance-Hess model was that newly synthesized ATP is retained in the mitochondria and is not quickly available for cytosolic processes. Their mitochondrial ATP synthesis mechanism did not comply with the chemiosmotic hypothesis. These assumptions in the Chance-Hess model are unfortunately incompatible with present biochemical knowledge. Nevertheless, the model of Chance and Hess was of course a very meritorious historical step in the computational analysis of biochemical systems. You will now find these issues discussed more extensively in the main text (Introduction; Methods, first paragraph and third paragraph) and Supplementary Material (first page and new section “Reasons for Employing a Coarse-grained Rather than Detailed Model of Glycolysis”).

*Most, if not all, results are presented as curves from the simulation with little information about the take-home messages. It is thus very difficult to read the key findings directly from the figures. For instance, it is my understanding that one of the two most important hypotheses drawn based on the simulation is that cells with high glycolytic capacity (which mimics ‘Warburgian’ cancer cells) consume glucose much more quickly than cells with lower glycolytic capacity after switching from low glucose to high glucose condition, thus being more competitive under conditions with frequent nutrient deprivation. To emphasize this point, I would recommend using one figure directly comparing glucose uptake fluxes in cells with different glycolytic capacities instead of the 10 figure panels currently included in Fig 4.*

 I followed your excellent suggestion and added a figure comparing the glucose uptake fluxes in the two cell types (new Figure 5) to illustrate this important message. I considered it helpful to retain the original Figure 4 to show effects on FBP levels and ATP synthesis, among others because it illustrates another important take-home message regarding the role of the ATP buffering mechanism in maintaining energy homeostasis. I also added extensive clarifications about the take home messages at many points in the text of the Results section.

 REFERENCES

 1.            H. R. Christofk, M. G. Vander Heiden, N. Wu, J. M. Asara, L. C. Cantley, Pyruvate kinase M2 is a phosphotyrosine-binding protein. *Nature* **452**, 181-186 (2008).

 2.            J. M. Berg, J. L. Tymoczko, L. Stryer, *Biochemistry*.  (W.H. Freeman and Company, ed. Sixth, 2007).

 3.            A. A. Shestov, X. Liu, Z. Ser, A. A. Cluntun, Y. P. Hung, L. Huang, D. Kim, A. Le, G. Yellen, J. G. Albeck, J. W. Locasale, Quantitative determinants of aerobic glycolysis identify flux through the enzyme GAPDH as a limiting step. *Elife* **3**,  (2014).

 4.            M. V. Liberti, Z. Dai, S. E. Wardell, J. A. Baccile, X. Liu, X. Gao, R. Baldi, M. Mehrmohamadi, M. O. Johnson, N. S. Madhukar, A. A. Shestov, I. I. C. Chio, O. Elemento, J. C. Rathmell, F. C. Schroeder, D. P. McDonnell, J. W. Locasale, A Predictive Model for Selective Targeting of the Warburg Effect through GAPDH Inhibition with a Natural Product. *Cell Metab* **26**, 648-659 e648 (2017).

 5.            W. J. Nelson, S. J. Nelson, P. Traub, Comparison of Ehrlich ascites tumour and mouse liver cells by analytical subcellular fractionation combined with a sensitive computational method for data analysis. *Hoppe-Seyler's Z. Physiol. Chem.* **362**, 903-918 (1981).

 6.            M. J. Lambeth, M. J. Kushmerick, A computational model for glycogenolysis in skeletal muscle. *Annals of Biomedical Engineering* **30**, 808-827 (2002).

 7.            P. J. Mulquiney, P. W. Kuchel, Model of 2,3-bisphosphoglycerate metabolism in the human erythrocyte based on detailed enzyme kinetic equations: equations and parameter refinement. *Biochem J* **342**, 581-596 (1999).

 8.            A. Marin-Hernandez, J. C. Gallardo-Perez, S. Rodriguez-Enriquez, R. Encalada, R. Moreno-Sanchez, E. Saavedra, Modeling cancer glycolysis. *Biochim Biophys Acta* **1807**, 755-767 (2011).

 9.            B. Chance, B. Hess, Spectroscopic evidence of metabolic control. *Science* **129**, 700-708 (1959).

 10.         B. Chance, D. Garfinkel, J. Higgins, B. Hess, A solution for the equations representing interaction between glycolysis and respiration in ascites tumor cells. *Journal of Biological Chemistry* **235**, 2426-2439 (1960).

 11.         K. van Eunen, J. A. Kiewiet, H. V. Westerhoff, B. M. Bakker, Testing biochemistry revisited: how in vivo metabolism can be understood from in vitro enzyme kinetics. *PLoS Comput Biol* **8**, e1002483 (2012).

 12.         F. A. Chandra, G. Buzi, J. C. Doyle, Glycolytic oscillations and limits on robust efficiency. *Science* **333**, 187-192 (2011).

 13.         J. H. van Heerden, M. T. Wortel, F. J. Bruggeman, J. J. Heijnen, Y. J. Bollen, R. Planque, J. Hulshof, T. G. O'Toole, S. A. Wahl, B. Teusink, Lost in transition: start-up of glycolysis yields subpopulations of nongrowing cells. *Science* **343**, 1245114 (2014).

# Peer review: Referee response for version 1

**DOI:** 10.5256/f1000research.17060.r36758

**Published:** 2018-09-11

**Authors:** Ranjan K. Dash

**Affiliations:** 1 Department of Biomedical Engineering, Medical College of Wisconsin and Marquette University, Milwaukee, WI, 53226, USA

This is an interesting original research article based on computational modeling to understand several questions related to cancer cell metabolism, specifically on the Warburg effects and related phenomena (high glucose uptake and lactate production by cancer cells despite sufficient oxygen supply; also very high transient glucose uptake one order of magnitude faster than the high steady state glucose uptake). In doing this, the author developed a new computational model integrating simplified "phenomenological" models of key lumped reactions in glycolysis, mitochondrial ATP synthesis coupled to oxygen consumption (oxidative phosphorylation; OxPhos), and cytosolic ATP consumption. I am not an expert in cancer cell metabolism, so I do not know how much experimental data are available for testing and calibrating the mathematical model. I trust the author has carefully considered all the important experimental data that are available for the calibration of the model. For example, I see the author has considered some key experimental data sets showing transients in several important variables in the mathematical model governing cellular metabolism in cancer cells (Figure 2). This is surely an important data sets for model calibration, as it depicts the transients of fructose biphosphate (FBP) content, ATP content, glucose uptake, lactate production, and oxygen consumption for two different glucose stimulation conditions in cancer cells (low glucose and high glucose conditions). These data show distinct characteristics in these important variables for these two perturbation conditions. Based on the calibrated model, the author used model simulations to gather several interesting insights into the cancer cell metabolism. Finally, the author has very well recognized the limitations of the model. I still wanted to point out few further limitations of the modeling.

 As I mentioned above, the modeling of the lumped reaction processes in this integrated model are highly phenomenological. The author considered two lumped reactions of the whole glycolysis process (termed as "head" and "tail" portion of the glycolysis), one reaction representing OxPhos, and one reaction for ATP consumption. I wonder what is the philosophy behind the formulated flux expressions for these lumped reactions. I see the author has not given any thermodynamic consideration in the modeling of these lumped reactions (e.g. reversible reaction fluxes satisfying the Haldane constraint, relating kinetic parameters to the Gibbs free energy of the lumped reactions). The author has also not included the stoichiometry of the biochemical species in the modeled reaction fluxes. For example, the modeled lumped reaction fluxes do not include square terms considering two ATP and ADP are involved in the lumped phosphorylation-dephosphorylation reactions. Also I am wondering if the author has individually parameterized these reactions fluxes prior to integration and testing the integrated model with the dynamic data in Figure 2.

 Besides these few modeling limitations, the author has done a great job in developing, calibrating and testing the model, and coming up with interesting conclusions regarding the operation of this complex metabolic system in cancer cell. I am not sure how these findings can be experimentally tested, but I would invite the author to put some of his thoughts on further testing experimentally these interesting model predictions. Besides, I have few other suggestions:

 1. I would suggest the author to show the model simulated reaction fluxes along with the model fittings in Figure 2.

 2. I would suggest the author using the acronym EATC throughout after it is defined.

 3. I did not see how the effect of blood flow is integrated into the model. I thought the model is for isolated cell experiments in a cuvette.

 4. The whole experimental system (cells in buffer) is considered as a single compartment. I am wondering how the model prediction would alter if one considers compartmentation (e.g. extracellular, cytosol, and mitochondria as separate compartments with transport of species in and out of the compartments).

I have read this submission. I believe that I have an appropriate level of expertise to confirm that it is of an acceptable scientific standard.

# Response

**Published:** 2018-12-10

**Authors:** Hans van Beek

**Affiliations:** Department of Experimental Vascular Medicine, Academic Medical Center of the University of Amsterdam, The Netherlands

Answer to reviewer Dash:

 Thank you very much for your constructive criticism. I addressed all of your suggestions and questions, despite your approval of the manuscript without reservations. I think this led to considerable improvement of the manuscript. Details are given below.

*… the modeling of the lumped reaction processes in this integrated model are highly phenomenological. The author considered two lumped reactions of the whole glycolysis process (termed as "head" and "tail" portion of the glycolysis), one reaction representing OxPhos, and one reaction for ATP consumption. I wonder what is the philosophy behind the formulated flux expressions for these lumped reactions.*

 The philosophy behind the flux expressions for the head and tail sections of glycolysis is explained in  considerably greater detail now in the main text and Supplementary Text.  This led to rewriting of the Methods section in the main text, in particular the first eight paragraphs, as well as of the Supplementary Text, in particular the first two and a half pages.

   The reasons for modeling lumped reaction processes rather than detailed enzymatic equations are addressed in the first and third paragraphs of the Methods section of the main text, in the first two paragraphs of the Supplementary Text and in response to a question by your fellow reviewer, Dr Dai.  In addition, a full section was added to the Supplementary Text, entitled “Reasons for Employing a Coarse-grained Rather than Detailed Model of Glycolysis”.

   Here some of the main considerations are given regarding the equations for the head and tail section of glycolysis. The flux equation for the lumped reaction of the head section is made to depend on the concentration of the substrates which are used in this section: ATP and glucose. The equation is similar as used for hexokinase by Marin-Hernandez et al. ( *1*) and by Mulquiney and Kuchel ( *2*). This seems a relevant equation because hexokinase is the entry point of the head section and an important control point of rate limitation in this section ( *3*), and this is the point where glucose and ATP are processed by the same enzyme. (ATP is of course also involved in the subsequent phosphofructokinase reaction.)

   Product inhibition of hexokinase is important in Ehrlich ascites tumor cells ( *4*). In Mulquiney and Kuchel’s model inhibitors of hexokinase bind to the glucose-bound form of the enzyme and binding is assumed there to be instantaneously in equilibrium. In the present model, inhibition is independent of the substrate-binding state and is not instantaneous, leading to slow inactivation (see main text, fifth paragraph of Methods and in the Supplementary Text above Eq. 1). Whether this simplified description of the head section is satisfactory was in the end judged by its description of the data: the fit to the input and output of the head section, glucose uptake and fructose 1,6-bisphosphate, appears good.

 The tail section of glycolysis was represented by an equation which depends on the input reactants of the tail section: FBP, ADP and NAD ^+^. Each is represented in the equation by a factor with a Michaelis-Menten constant. NADH, which is a product of the GAPDH reaction, not only negatively affects the forward net reaction rate in enzyme kinetic equations by increasing reverse flux in GAPDH ( *1, 5*), but NADH is also an inhibitor of GAPDH in EATC ( *6*). These negative effects of high NADH levels are represented by a factor with an inhibition constant. The factor depending on FBP not only reflects the saturable dependence of the tail part on phosphorylated substrate, but also allosteric activation of the pyruvate kinase enzyme in the tail section ( *7, 8*). Therefore the complex kinetics of the enzymes in the tail part is represented by an approximation containing a limited number of parameters that are estimated from the experimental data on the intact system. The equation roughly reflects the kinetics of glyceraldehyde 3-phosphate dehydrogenase, which is an important limiting step in the tail part of glycolysis ( *9*). The effect of ADP as a substrate for the reactions in the glycolytic tail section (phosphoglycerate kinase and pyruvate kinase) is also reflected by a factor with a Michaelis-Menten type constant.

   Discussion of the equations for ATP consumption is given in the Supplementary Text, covering about one page around Equations 5 and 6. The equation for oxidative phosphorylation was described and used previously by others and myself: see discussion in Van Beek ( *10*) and Hettling and Van Beek ( *11*) where a detailed model of oxidative phosphorylation described by Vendelin et al. ( *12*) and Korzeniewski ( *13*) is also discussed.  These considerations have now been extended in the main text and the Supplementary Text.

*I see the author has not given any thermodynamic consideration in the modeling of these lumped reactions (e.g. reversible reaction fluxes satisfying the Haldane constraint, relating kinetic parameters to the Gibbs free energy of the lumped reactions).*

 Your fellow reviewer, Dr Dai, gave a similar comment. I have therefore included the following answer also in the answer to her.

 Glycolysis is considered by biochemists to be essentially irreversible, in particular  the hexokinase, phosphofructokinase and pyruvate kinase reactions (see for instance the textbook by Berg, Tymocko, Stryer. Biochemistry, Sixth Edition, 2007, e.g. page 460, ref. ( *14*)). In addition the glyceraldehyde 3-phosphate dehydrogenase reaction may also be an important limiting step in cells showing the Warburg effect ( *9, 15*). To produce glucose in the reverse direction (fructose 1,6-bisphosphate to glucose) the enzyme glucose 6-phosphatase is required to bypass the irreversible hexokinase reaction. Glucose 6-phosphatase’s activity in Ehrlich ascites cells is low ( *16*), while its activity in the liver, which can produce glucose, is high. The tail section of glycolysis is also considered to be virtually irreversible and two additional enzymes (pyruvate carboxylase and phoshoenolpyruvate carboxykinase) are required to enable conversion of pyruvate in the direction of glucose, bypassing pyruvate kinase (see ( *14*), p. 460-463).

   I had implemented two existing detailed models of the glycolytic chain and used these to explore the reversibility of the reactions. The first model was based on the model of Lambeth and Kushmerick ( *5*), extended with the equation for hexokinase given by Mulquiney and Kuchel ( *2*). The second model was based on the model of Marin-Hernandez et al. ( *1*) for cancer cells. Both models represent the ten enzymatic steps in the glycolytic chain and incorporate both the forward and reverse reactions for all the steps. All these equations take the Haldane relation into account, and constraints based on thermodynamics are obeyed. Simulations with both models showed that at least one reaction in the head as well as the tail section of the glycolytic chain showed reverse fluxes that were extremely small relative to the forward fluxes. The results are discussed in more detail in the revised Supplementary Text, section titled “Effect of Reverse Fluxes on the Glycolytic Model”.

   These simulations illustrate the biochemical concept that glycolysis is essentially irreversible. In order to reduce the model as much as possible, the reverse reactions in glycolysis were therefore neglected. However, the lactate dehydrogenase reaction is included in fully reversible form and lactate is taken up for mitochondrial metabolism if glucose is depleted in the simulations. Mitochondrial oxidative phosphorylation coupled to oxygen consumption is also modeled as being essentially irreversible, as generally accepted by biochemists. However, these considerations point to a limitation: the model is not suitable for determining the magnitude of small reversed fluxes in the glycolytic chain under conditions where glycolytic products (pyruvate, NADH, ATP) are high and glucose concentration is very low. Reverse fluxes may be revealed by isotope exchange experiments. These considerations are now included in the Methods section of the main paper (8th paragraph) and the Supplementary Text (section title “Effect of Reverse Fluxes on the Glycolytic Model”).

*The author has also not included the stoichiometry of the biochemical species in the modeled reaction fluxes. For example, the modeled lumped reaction fluxes do not include square terms considering two ATP and ADP are involved in the lumped phosphorylation-dephosphorylation reactions. Also I am wondering if the author has individually parameterized these reactions fluxes prior to integration and testing the integrated model with the dynamic data in Figure 2.*

 The kinetic rate equations are not based on mass action kinetics, but represent saturable enzyme kinetics with Michaelis-Menten type constants. When 2 ATP are consumed to phosphorylate glucose to fructose 1,6-bisphosphate, this occurs in two separate enzyme steps in the head section of the glycolytic chain which both show saturable kinetics. Therefore the rate equation was modeled with saturable kinetics without square terms, resembling the kinetics of hexokinase, a major control point of rate limitation in the glycolytic head section in cancer cells ( *3*). These considerations have now been more extensively explained in the revised manuscript. However, note that the stoichiometry of the biochemical reactions was accounted for accurately in the differential equations that describe the mass balances of the species.

   For instance, the stoichiometry of the lumped head section reaction is           

                       glucose + 2 ATP -> fructose 1,6-bisphosphate + 2 ADP

 (for clarity the reaction is given without electric charges and H ^+^ ions). The stoichiometric coefficient 2 is accounted for in algebraic Eq. 10 (Supplementary Text), and the total stoichiometry of the reaction is embedded in differential equations 16, 19, 20 and 21. This also applies to other reactions.

   I had not individually parameterized the rate equations prior to integrating them in the model. The consideration was that no data are available for the isolated head section or isolated tail section. If the enzymes for both these sections were isolated, their behavior would likely be affected by the isolation procedure and their isolated state. A further consideration was that the behavior of head or tail section would be best represented by the data on the intact cell, where the input and output of the head section is for instance well represented in the data by its input (glucose uptake) and output (FBP).  This is discussed in the second paragraph in the Supplementary Text.

*I am not sure how these findings can be experimentally tested, but I would invite the author to put some of his thoughts on further testing experimentally these interesting model predictions.*

 Here follow some ideas on further testing of the model of metabolic responses of Ehrlich ascites cells *in vitro*:

- the model predicts that a major part of the glucose taken up in an experiment as in Fig. 2 is stored in the cell. The time course, target molecules and amounts of storage could be investigated using labeled glucose (e.g. with carbon-13) and the time course of incorporation of label in pyruvate, lactate, glycolytic intermediates and side reactions of glycolysis (e.g. serine synthesis, pentose phosphate pathway) may be investigated

- a prediction of the model is that there is no initial stimulation of respiration after addition of a high concentration of glucose if a high concentration of pyruvate is simultaneously present; the predicted time courses of lactate production and respiration during one hour can be measured.

 -  the time course of metabolites not yet used for parameter estimation may be measured. An interesting candidate is NADH, whose time course might be measured by its autofluorescence. ATP and/or ADP concentrations can be measured by fluorescent probes or NMR spectroscopy. Glucose may be measured using a fluorescent probe.

 -  the effect of partial inhibition or lowered expression of the head or tail portion of glycolysis on the time course of glucose uptake, FBP accumulation and lactate production can be simulated and tested experimentally.

- the model predicts that feedback from glycolytic intermediate pools limits the initially very high glucose uptake and FBP accumulation: this may be investigated by searching for chemicals which block the inhibition. Binding of the chemicals may be used to identify potential binding sites. The levels of glycolytic intermediates may be manipulated to identify potential candidates for feedback inhibition. Enzymes isolated from the Ehrlich ascites tumor cells may be isolated to test for their sensitivity to inhibition by glycolytic intermediates. This also applies to hexokinase bound to mitochondrial membranes.
- the time course of disinhibition after glucose and glycolytic intermediate depletion is still relatively imprecisely determined: this may be tested and fine-tuned by altering duration and extent of glucose depletion (zero glucose or slightly higher).
- genetic modification of genes may reveal key controllers of the dynamic regulation of glycolysis in the ascites tumor cells
- measurement of the rate of phosphoryl group transfer by using NMR saturation transfer or ^18^O labeling measurements

 

 Possible tests of the prediction of responses of tumor cells *in vivo* are:

- perfuse a cancer tissue with a pump, imposing fluctuating blood flow to be compared with constant blood flow. The preparation used by Vaupel ( *17*) may be well suited because it has a distinct blood vessel supply and homogeneous cell population. Measure NADH fluorescence and tissue oxygen concentration continuously. Measure metabolite concentrations as function of the phase of the blood flow cycle. If sufficient cell mass is available: measure phosphoryl group transfer rate by saturation transfer NMR. Repeat this after partial inhibition of glycolysis, possibly at the level of glyceraldehyde 3-phosphate dehydrogenase. If different cell types are present: measure NADH fluorescence and metabolites with fluorescent labels in individual cells. Measure markers of cell death or apoptosis after a period of fluctuating blood flow in comparison with a period of constant blood flow.
- remove glucose from the perfusate but retain lactate. The prediction is this will be adequate to sustain tumor cell metabolism with constant flow, but it will cause an energy crisis during cycling blood flow.
- measure growth and proliferation of tumor tissue with and without inhibition of the feedback loop in case a suitable inhibitor has been identified.
- set up a system for perfusion of a cell culture containing different cell types with different glycolytic capacities. Perfuse with constant oxygen and glucose supply and with fluctuating oxygen and glucose supply. Measure markers of cell damage after a period of constant supply and after a period of fluctuating supply. Measure intracellular concentrations with fluorescent probes. Such probes are for instance available for ATP and glucose.

 

 These suggestions for experimental testing have now been added to the main text (Discussion, second paragraph for testing metabolic model *in vitro*, and ninth paragraph for testing predictions for tissue *in vivo*).

*Besides, I have few other suggestions:*

*1. I would suggest the author to show the model simulated reaction fluxes along with the model fittings in Figure 2.*

 I followed this excellent suggestion. I have added a figure 2B with the simulated reaction fluxes applying to the responses in the previous Fig. 2, now designated Fig. 2A.

*2. I would suggest the author using the acronym EATC throughout after it is defined.*

 I have followed your suggestion in the revised manuscript.

*3. I did not see how the effect of blood flow is integrated into the model. I thought the model is for isolated cell experiments in a cuvette.*

 In the original submission the description of equations for blood flow and diffusion was only given in the Results, with all details of the blood flow and diffusion equations in the supplementary material, equations 24-31. Description of the extended model of cell metabolism with transport equations in tissue is now explicitly included in the Methods section of the revised manuscript with reference to the details given in the Supplementary Text.

   In the abstract the sentence “The model, extended with equations for glucose and O _2_ transport in tissue, is subsequently used to predict metabolism in tumor cells during fluctuations of tissue blood flow resulting in cycling hypoxia.” was rephrased: “The model is subsequently extended with equations for glucose and O2 transport to predict the role of metabolism during fluctuations of blood flow in tumor tissue.”

*4. The whole experimental system (cells in buffer) is considered as a single compartment. I am wondering how the model prediction would alter if one considers compartmentation (e.g. extracellular, cytosol, and mitochondria as separate compartments with transport of species in and out of the compartments).*

 Three compartments actually exist in the present model: extracellular, cytosolic and mitochondrial. All glycolytic intermediates are confined to the cytosolic compartment and their concentration changes are calculated based on the cytosolic volume. Fructose 1,6-bisphosphate and other phosphorylated glycolytic intermediates do not cross the cell membrane or the inner mitochondrial membrane, as usually assumed in biochemistry. ADP enters the mitochondrial compartment in exchange for ATP synthesized in the mitochondria. Their rate of change in the cytosol is calculated based on the cytosolic volume.

   Oxygen, glucose, lactate and pyruvate are assumed to have the same concentration in the extracellular and cytosolic compartment and their concentration is calculated based on the sum of extracellular and cytosolic volumes. This is of course a simplification of the real situation. The cell membrane causes little impediment for oxygen: the apparent Michaelis-Menten constant for oxygen, K _O2,mit_, was determined by Froese ( *18*)  to be 0.26 μM based on the O _2_ concentration in the environment of the cell. This shows that the oxygen concentration gradient between environment and cell interior is very small.

   Transport of glucose into the cell is part of the lumped equation for the head section of glycolysis, as is now emphasized in the revised manuscript. The transport of glucose across the cell membrane in EATC was measured by Saha and Coe ( *19*). They found two transport systems: a high affinity one with K _m_=100 µM and V _max_ = 467 µM/s and a low affinity one with K _m_=25 mM and V _max_ = 3333 µM/s. I tested the effect of adding the glucose transport equation with these parameters to the model, which means that glucose transport was separate from the head section. The parameters of the model were then estimated again by the same procedure, keeping the glucose parameters fixed at the value measured by Saha and Coe. The fit of the model to the data was virtually indiscernible from that in Fig. 2A and the cost function value was even slightly lower. Changes in parameter values were small: for instance, the V _max,head_ was 394 μM/s without the separate glucose transport equations, and 376 μM/s with the additional glucose transport equations. However, there was one exception: the K _glucose,head _which represents the apparent affinity of the head section for glucose fell from 51 μM to 4.9 μM, and the calculated glucose concentration in the cytosol was decreased.  Note that in the model in the present manuscript the contribution of glucose transport is integral part of the head section of glycolysis and parameters are estimated for the behavior of this entire head section, taking measured glucose uptake and FBP accumulation into account, as is now emphasized in the revised text.

   Lactate and pyruvate are transported via highly active transporters: lactate transport across the cell wall of EATC is characterized by a Vmax of 1866 µM/s with Km=4.7 mM and pyruvate transport is characterized by a Vmax of 1290 µM/s with Km=8.5 mM ( *20*).

   Although the finite permeability of the cell membrane causes concentration differences and some delay between the extracellular and cytosolic compartment, the arguments given above suggest that these effects are limited. To keep the model as simple as possible, the transport processes across the cell membrane were therefore not explicitly included in the model. These considerations are now added to Supplementary Text, in the paragraphs following Equations 16 and 18.

 REFERENCES

 1.            A. Marin-Hernandez, J. C. Gallardo-Perez, S. Rodriguez-Enriquez, R. Encalada, R. Moreno-Sanchez, E. Saavedra, Modeling cancer glycolysis. *Biochim Biophys Acta* **1807**, 755-767 (2011).

 2.            P. J. Mulquiney, P. W. Kuchel, Model of 2,3-bisphosphoglycerate metabolism in the human erythrocyte based on detailed enzyme kinetic equations: equations and parameter refinement. *Biochem J* **342**, 581-596 (1999).

 3.            A. Marin Hernandez, S. Rodriguez-Enriquez, P. A. Vital-Gonzalez, F. L. Flores-Rodriguez, M. Macias-Silva, M. Sosa-Garrocho, R. Moreno-Sanchez, Determining and understanding the control of glycolysis in fast-growth tumor cells. Flux control by an over-expressed but strongly product-inhibited hexokinase. *FEBS J* **273**, 1975-1988 (2006).

 4.            D. P. Kosow, I. A. Rose, Origin of the delayed feedback control of glucose utilization in ascites tumor cells. *Biochem Biophys Res Commun* **48**, 376-383 (1972).

 5.            M. J. Lambeth, M. J. Kushmerick, A computational model for glycogenolysis in skeletal muscle. *Annals of Biomedical Engineering* **30**, 808-827 (2002).

 6.            S. Bagui, M. Ray, S. Ray, Glyceraldehyde-3-phosphate dehydrogenase from Ehrlich ascites carcinoma cells. Its possible role in the high glycolysis of malignant cells. *Eur J Biochem* **262**, 386-395 (1999).

 7.            M. S. Jurica, A. Mesecar, P. R. Heath, W. Shi, T. Nowak, B. L. Stoddard, The allosteric regulation of pyruvate kinase by fructose-1,6-bisphosphate. *Structure* **6**, 195-210 (1998).

 8.            H. R. Christofk, M. G. Vander Heiden, N. Wu, J. M. Asara, L. C. Cantley, Pyruvate kinase M2 is a phosphotyrosine-binding protein. *Nature* **452**, 181-186 (2008).

 9.            A. A. Shestov, X. Liu, Z. Ser, A. A. Cluntun, Y. P. Hung, L. Huang, D. Kim, A. Le, G. Yellen, J. G. Albeck, J. W. Locasale, Quantitative determinants of aerobic glycolysis identify flux through the enzyme GAPDH as a limiting step. *Elife* **3**,  (2014).

 10.         J. H. van Beek, Adenine nucleotide-creatine-phosphate module in myocardial metabolic system explains fast phase of dynamic regulation of oxidative phosphorylation. *Am J Physiol Cell Physiol* **293**, C815-829 (2007).

 11.         H. Hettling, J. H. van Beek, Analyzing the functional properties of the creatine kinase system with multiscale 'sloppy' modeling. *PLoS Comput Biol* **7**, e1002130 (2011).

 12.         M. Vendelin, O. Kongas, V. Saks, Regulation of mitochondrial respiration in heart cells analyzed by reaction-diffusion model of energy transfer. *Am J Physiol Cell Physiol* **278**, C747-C764 (2000).

 13.         B. Korzeniewski, Regulation of ATP supply during muscle contraction: theoretical studies. *Biochem J* **330**, 1189-1195 (1998).

 14.         J. M. Berg, J. L. Tymoczko, L. Stryer, *Biochemistry*.  (W.H. Freeman and Company, ed. Sixth, 2007).

 15.         M. V. Liberti, Z. Dai, S. E. Wardell, J. A. Baccile, X. Liu, X. Gao, R. Baldi, M. Mehrmohamadi, M. O. Johnson, N. S. Madhukar, A. A. Shestov, I. I. C. Chio, O. Elemento, J. C. Rathmell, F. C. Schroeder, D. P. McDonnell, J. W. Locasale, A Predictive Model for Selective Targeting of the Warburg Effect through GAPDH Inhibition with a Natural Product. *Cell Metab* **26**, 648-659 e648 (2017).

 16.         W. J. Nelson, S. J. Nelson, P. Traub, Comparison of Ehrlich ascites tumour and mouse liver cells by analytical subcellular fractionation combined with a sensitive computational method for data analysis. *Hoppe-Seyler's Z. Physiol. Chem.* **362**, 903-918 (1981).

 17.         P. Vaupel, H. Günther, J. Grote, Atemgaswechsel und Glucosestoffwechsel von tumoren (DS-Carcinosarkom) in vivo. I. Experimentelle Untersuchungen der versorgungsbestimmenden parameter. *Z ges exp Med* **156**, 283-294 (1971).

 18.         G. Froese, The respiration of ascites tumor cells at low oxygen concentration. *Biochimica en Biophysica Acta* **57**, 509-519 (1962).

 19.         J. Saha, E. L. Coe, Evidence indicating the existence of two modes of glucose uptake in Ehrlich ascites tumor cells. *Biochem Biophys Res Commun* **26**, 441-446 (1967).

 20.         T. L. Spencer, A. L. Lehninger, L-Lactate transport in Ehrlich ascites-tumour cells. *Biochem J* **154**, 405-414 (1976).
